# Directing group-assisted C–H functionalization of 2-arylbenzo[*f*]isoquinoline: a robust route to decumbenine B analogues with photophysical applications

**DOI:** 10.1039/d5ra09256f

**Published:** 2026-01-05

**Authors:** Sampa Mondal, Sudipta Ghara, Sk Abulkalam Azad, Paritosh Barik, Prasenjit Sen, Nayim Sepay, Prasanta Patra, Subrata Mahanta, Laksmikanta Adak, Shubhankar Samanta

**Affiliations:** a Department of Chemistry EB 2, Sector I, Salt Lake Kolkata-700064 West Bengal India chemshubha@gmail.com; b Department of Chemistry, Indian Institute of Engineering Science and Technology Shibpur, Botanic Garden Howrah 711103 West Bengal India; c Department of Chemistry, National Institute of Technology Jamshedpur India; d Department of Chemistry, Lady Brabourne College Kolkata-700017 West Bengal India; e Department of Chemistry, Jhargram Raj College Jhargram India

## Abstract

An efficient transition metal-catalysed regioselective short synthetic route to Decumbenine B analogues is prepared from the green precursor 2-arylbenzo[*f*]isoquinoline (obtained *via* urea-promoted neat reaction). The single precursor capable of introducing –CH_2_OH, –X(–Br/–Cl), –SePh groups *via* Ru/Pd/Cu-catalyzed C–H functionalization. Theoretical and experimental investigations confirmed that Pd(ii) and Cu(ii) preferentially interact with the isoquinoline precursor to drive halogenation and selenation, respectively, while Ru(ii) effectively catalyzes the hydroxymethylation reaction. The protocol is extended to newly synthesized fluorescent molecules that exhibit aggregation-induced emission (AIE) and toxic compound sensing properties. Consequently, this approach assists us in accessing a diverse array of naturally occurring analogues and facilitates more efficient and effective biological studies with minimal effort.

## Abstract 2

A regioselective C–H functionalizations [–CH_2_OH, –Br/Cl and –SePh)] *via* Ru/Pd/Cu catalyst has been developed for the synthesis of Decumbenine B analogues. DFT studies for metal selection and toxic-compound sensing are attraction of this protocol.

## Graphical abstract

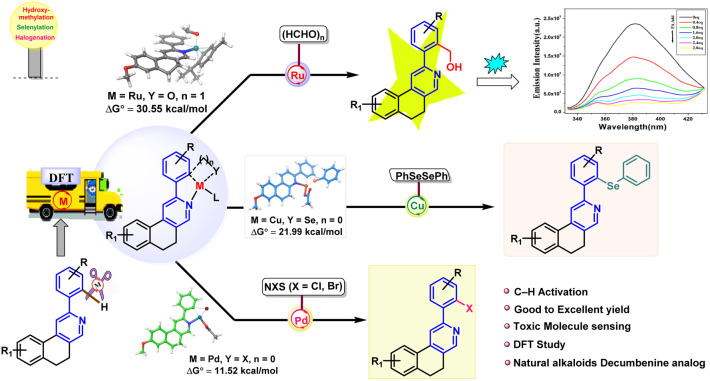

## Introduction

**Fig. 1:**
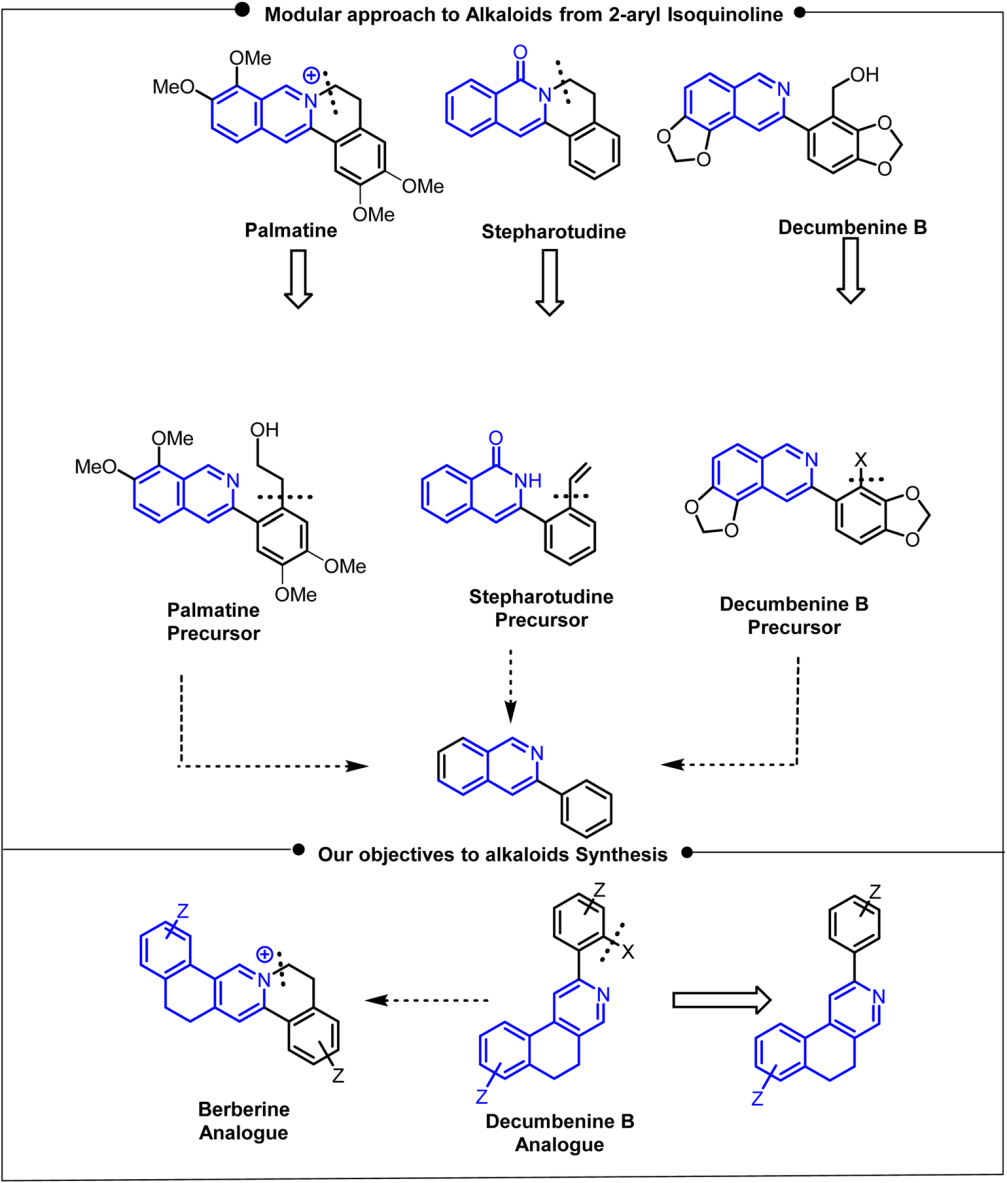
Objective of the developed protocol for alkaloid analogue synthesis.

The development of synthetic pathways for physiologically active alkaloid analogues is a key component of organic synthesis, which not only combines chemistry with nature but also provides novel opportunities in the fields of pharmacology and biomedicine.^1^ These bio-relevant molecules are continuously employed in both *in vivo* and *in vitro* studies, including those on cancer, Alzheimer's disease, malaria, HIV, depression, amnesia, and COVID-19, a more recent scenario.^2–5^ However, functionalization is a crucial step for reducing toxicity or enhancing cell permeability; consequently, transition-metal-catalyzed C–H functionalization has emerged as one of the most recent and effective approaches. It is employed through the combination of directing groups with coordinating atoms, particularly O, N, S, C, and P along with metal atoms, such as Rh, Pd, and Ir, where mostly 5/6-membered heterocycle intermediates are formed.^6^ Among various scaffolds, the 2-arylisoquinoline skeleton (Fig. 1) is an important synthetic precursor for directing group-assisted C–H functionalization, offering a rapid route to the synthesis of drugs, functional materials, and natural products.^7^ The main structural framework of this parent isoquinoline nucleus has an N-atom, which readily coordinates the metal ions with the proximal aromatic C–H bond, resulting in the formation of an organometallic intermediate; hence, the functionalization takes place.

**Fig. 2:**
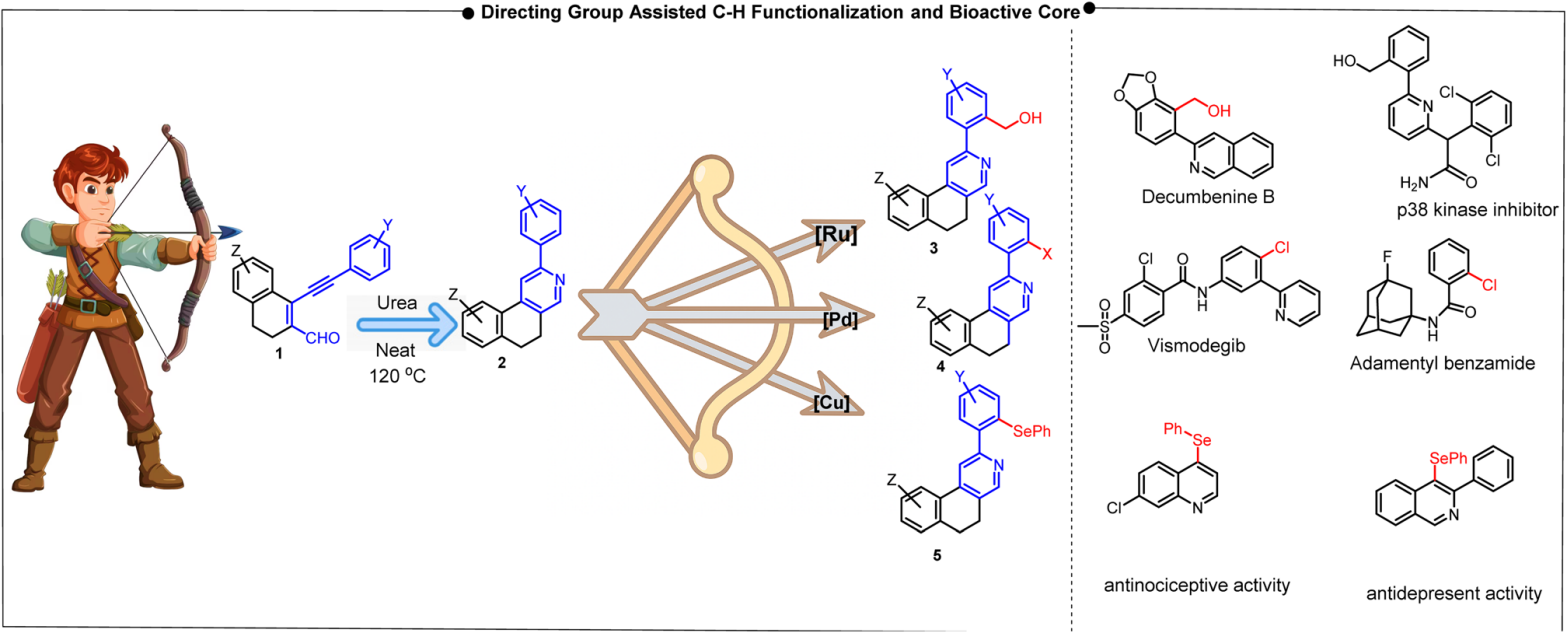
Outline of directing group-assisted C–H functionalizations.

Consequently, in the literature observations, it is found that the isoquinoline-fused alkaloids, such as Berberine,^8^ Palmatine, and Stepharotudine, and functionalized natural alkaloids, such as Decumbenine B analogue, can be obtained from a 2-arylisoquinoline precursor.^7^ These compounds exhibit a broad spectrum of biological activities, including antiviral, anticancer, antibacterial, antihypertensive, antidiabetic, and other therapeutic effects. However, alkaloids such as Berberine and Decumbenine B exhibit side effects, including gastrointestinal issues, allergic reactions, and bilirubin displacement. This limitation can be overcome through substrate modification, maintaining the fundamental molecular framework.^9^ Specifically, 2-arylisoquinoline acts as a crucial precursor for the synthesis of the Decumbenine B analogue through hydroxymethylation *via* C–H activation. Further modification through benzene-ring fusion affords benzo[*f*]isoquinoline, thereby providing a novel scaffold for C–H activation. The bending nature of the new directing group of the benzo[*f*]isoquinoline backbone facilitates functionalization, which increases the yields of the Decumbenine B analogue and reduces the reaction time. A strategic retrosynthetic analysis of the isoquinoline-fused alkaloids berberine and stepharotudine reveals a key intermediate that can be obtained *via* C–H functionalization (Fig. 1).^10^ Furthermore, hydroxymethylation, halogenation, and selenylation of 2-arylisoquinoline may help to prepare these alkaloid precursors,^11–16^ where –CH_2_OH or –X(–Cl/–Br/–I, and –SePh) groups can easily be achieved *via* chelating-assisted transition metal-catalyzed reactions, such as ruthenium^17,20,21^ and palladium,^11,22^ which lowers the activation energy and facilitates functionalization. It has been noticed that different bioactive compounds have these essential functionalities –CH_2_OH/–X(–Cl/–Br/–I or –SePh) to exhibit pharmacological activities (Fig. 2).^17–19^ Our group developed a single synthetic precursor (2) that was subsequently transformed into various functionalized 2-arylisoquinolines using transition metal-catalyzed (Ru, Pd and Cu) C–H activation processes to obtain biologically relevant compounds or building blocks.

In continuation of our efforts toward heterocyclic synthesis under sustainable conditions, we commenced our reaction from the substrate 6-methoxy-1-(phenylethynyl)-3,4-dihydronaphthalene-2-carbaldehyde using a urea-promoted reaction, which easily afforded 2-arylbenzo[*f*]isoquinoline in excellent yields.^23–28^ Hence, the fused isoquinoline can be called a green precursor. This fused isoquinoline scaffold has the inherent functionality to activate the proximal site of the aromatic C–H bond at the 2-position, thereby enabling the efficient formation of various substituted products in good yields (Fig. 2).

## Results and discussion

To establish the optimal reaction conditions for hydroxymethylation *via* C–H activation of 2-arylbenzo(*f*)isoquinoline, we performed a series of experiments with different reaction parameters. In our initial study, 8-methoxy-2-phenyl-5,6-dihydrobenzo[*f*]isoquinoline (2a) was selected as a model substrate for hydroxy methylation *via* C–H activation. The reaction was carried out using paraformaldehyde (3 equiv.), ZnBr_2_ (0.5 equiv.) and AcOK (0.5 equiv.) in 2 mL of DCE, with [Ru(*p*-cymene)Cl_2_]_2_ (10 mol%) as the catalyst under a nitrogen atmosphere at 120 °C for 3 hours (Table 1, entry 1), affording the fluorescent active hydroxymethylated analogue (3a) in 85% yield. When Pd(ii) was used in place of Ru(ii), satisfactory results were not obtained for the directed –CH_2_OH incorporation (entries 2 and 3). The presence of the ligand triphenylphosphine along with Pd(ii) was inactive towards *ortho* C–H bond activation (entry 6). Only a 5% product of 3a was obtained in the combination of Pd and Cu dual catalysts (entry 4). Although NMP was used as an alternative solvent for Ru(ii)-catalyzed C–H activation, the yield of the desired product was very low (entry 9). This protocol was also found to be ineffective when DMSO, DMF, or CH_3_CN was employed as the solvent with the Ru(ii) catalyst (entries 7, 8 and 10).

**Table 1:** Optimization of the chelating-assisted hydroxymethylation^a^

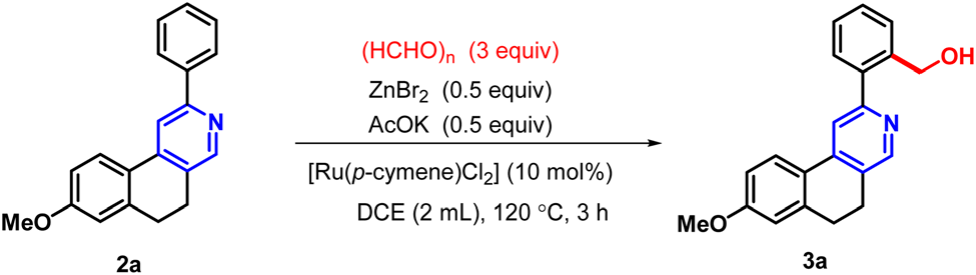		
Entry	Variation from the standard condition	Yield (%)
1	No variation	85
2	Using [Pd(PPh_3_)_2_Cl_2_] instead of [Ru(*p*-cymene)Cl_2_]	0
3	Using Pd(OAc)_2_ instead of [Ru(*p*-cymene)Cl_2_]	0
4	Using [Pd(OAc)_2_/Cu(OAc)_2_] instead of [Ru(*p*-cymene)Cl_2_]	5
5	Using Cu(OAc)_2_ instead of [Ru(*p*-cymene)Cl_2_]	0
6	Using Pd(OAc)_2_/PPh_3_ instead of [Ru(*p*-cymene)Cl_2_]	0
7	Using DMF instead of DCE	0
8	Using DMSO instead of DCE	0
9	Using NMP instead of DCE	18
10	Using CH_3_CN instead of DCE	0
11	Using RuCl_2_ instead of [Ru(*p*-cymene)Cl_2_]	65

a Reagent and conditions: substrate (0.1 mmol), catalyst (10 mol%), (HCHO)_*n*_ (90.1 mg), KOAc (0.05 mmol), ZnBr_2_ (0.05 mmol), solvent (2 mL).

After optimizing the reaction conditions (entry 1, Table 1), we explored C–H functionalization (Table 2) with the variation of different 2-arylbenzo(*f*)isoquinolines in the presence of paraformaldehyde to obtain a series of Decumbenine B analogues, which were used in biological studies. An electron-donating group on the naphthyl ring, such as a methoxy (–OMe) group, provided the desired product with up to 98% yield (3k). C–H activation was well tolerated by electron-rich and electron-withdrawing groups, such as a methoxy (–OMe) group at the 7-position of the naphthyl ring and fluoro (–F) substituents on the 2-aryl moiety, leading to the formation of compound 3e with 72% yield. Interestingly, C–H activation is facilitated when both components of the directing group are electron-rich, such as the –OMe group at the 6-position of the naphthyl ring and –Me groups in the 2-aryl part, affording 3g in 80% yield. The unsymmetrical aryl group directed the activation toward the less hindered position, affording the hydroxymethyl-substituted derivative with a good yield of 71%, 3f. However, a moderate yield of 3c was obtained for the unsubstituted 2-phenyl-5,6-dihydrobenzo[*f*]isoquinoline, and the same yield proportion of 3i was achieved for the corresponding -Br-substituted derivative.

**Table 2:** Decumbenine B analogue synthesis *via* C–H activation^a^

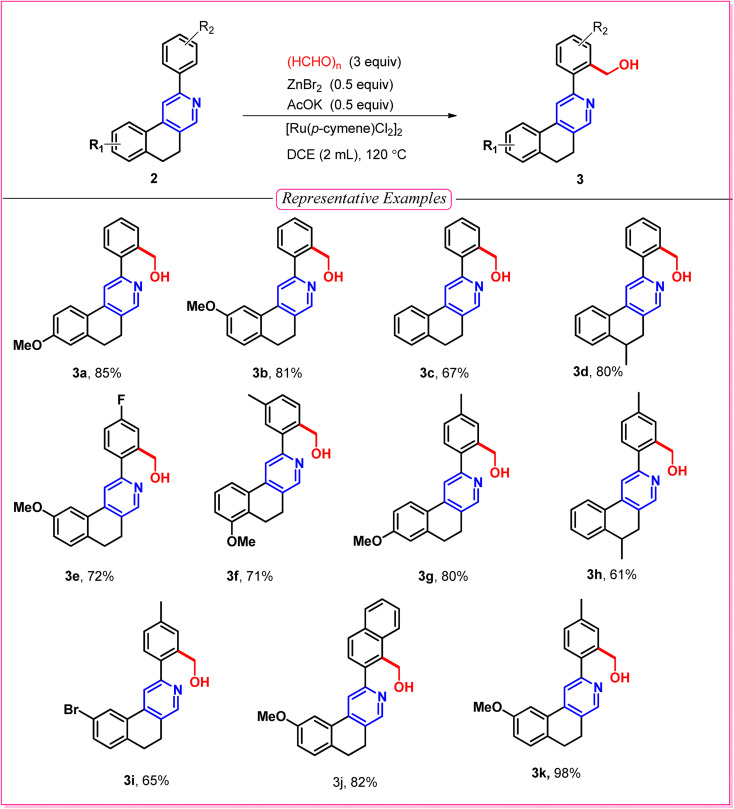

a Reagent and conditions: substrate (0.1 mmol), [Ru(*p*-cymene)Cl_2_]_2_ (10 mol%), (HCHO)_*n*_ (3 equiv.), KOAc (0.05 mmol), ZnBr_2_ (0.05 mmol), DCE (2 mL).

After hydroxymethylation, we want to explore the directing group-assisted C–H halogenation reaction in 2-arylbenzo[*f*]isoquinoline (2), as the corresponding products containing halogen atoms from 2 substrates could have greater capability for further functionalization *via* different types of cross-coupling reactions. We are pleased to report the successful establishment of the optimized reaction conditions for the C–H halogenation of 8-methoxy-2-phenyl-5,6-dihydrobenzo[*f*]isoquinoline (2a). The optimal protocol employs Pd(OAc)_2_ (10 mol%) and NBS (1 equiv.) in acetonitrile at 100 °C. This protocol was further applied to various substituted 2-arylbenzo[*f*]isoquinoline, selectively affording monobromination products (4a–4e). After bromination, we explored the chlorination reaction using NCS (1 equiv.) and Pd(OAc)_2_ (10 mol%) in acetonitrile and obtained effective yields of 4f and 4g. The optimized conditions were also applied to 9-bromo-2-phenyl-5,6-dihydrobenzo[*f*]isoquinoline for chlorination in the presence of *N*-chlorosuccinimide, and effective yields were obtained for difunctionalization product 4h. The difunctionalization can be explained by lowering the stability of the chelating Pd(ii) complex with the electron-withdrawing –Br substituent, reslting in poor regioselectivity (Table 3).

**Table 3:** Decumbenine B analogue synthesis *via* C–H activation^a^

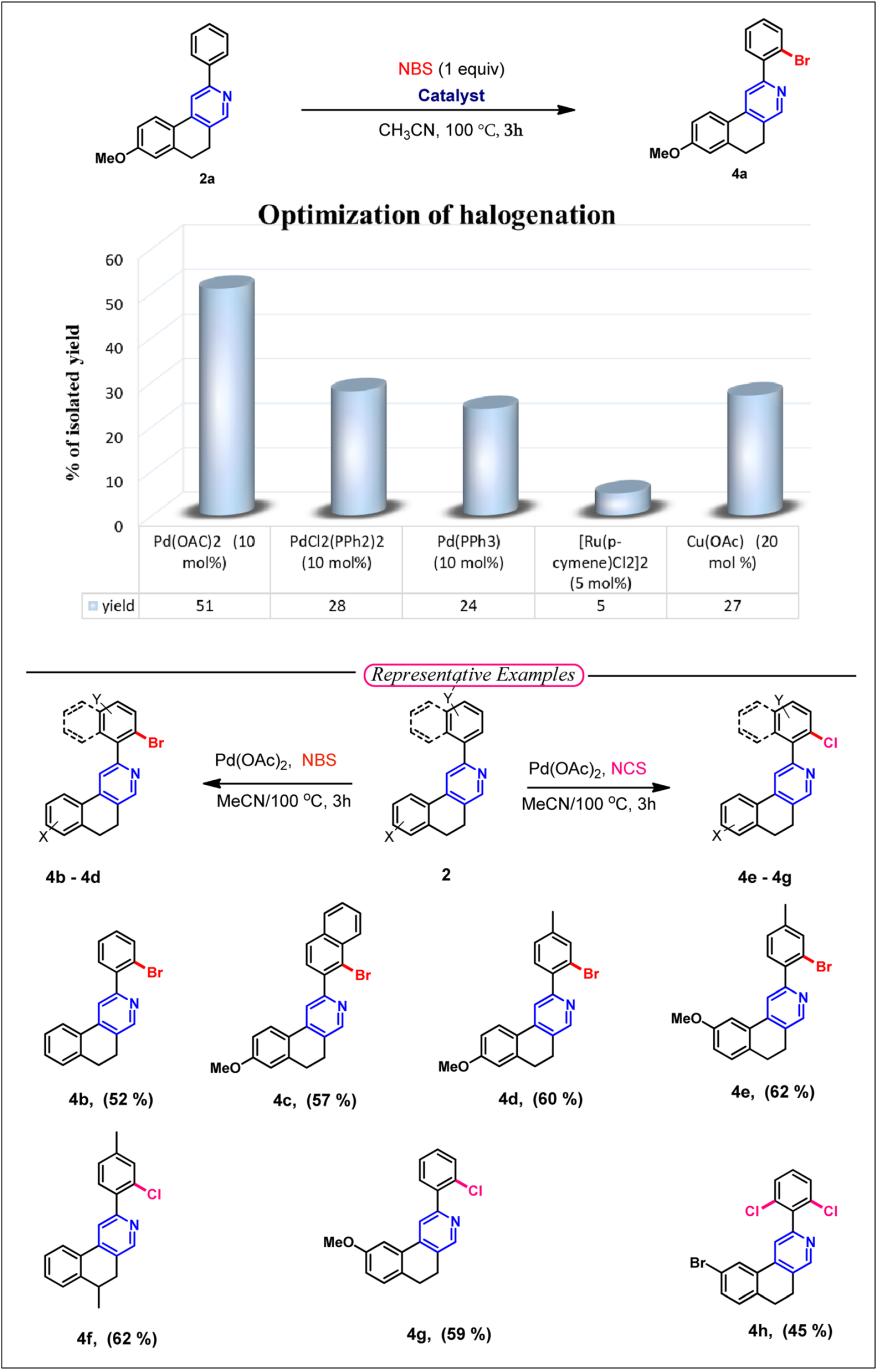

a Reagent and conditions: substrate (0.1 mmol), NBS (0.1 mmol) or NCS (0.1 mmol), Pd(OAc)_2_ (10 mol%), CH_3_CN (2 mL).

**Scheme 1:**
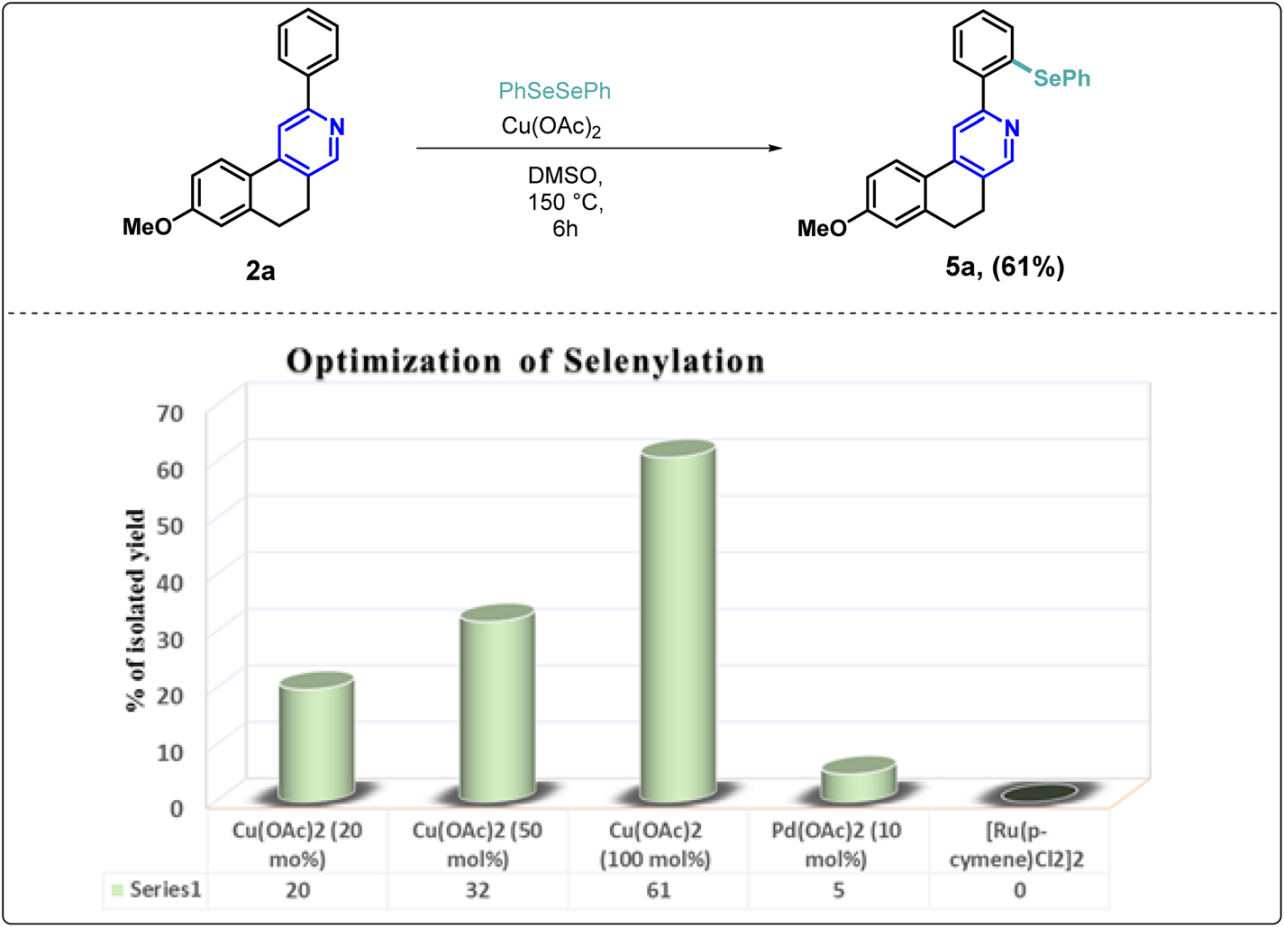
Functional group-directed regio-selective selenylation.

In medicinal chemistry and drug development, molecules that embrace carbon-selenium-carbon connectivity, known as organoselenides, have gained significant attention due to their broad spectrum in therapeutic activities.^29^ Consequently, we tested the selenylation of our synthesized scaffolds (2a) *via* direct C–H activation. In the literature, we observe that palladium, ruthenium, rhodium, and copper catalysts are used for the selenylation reaction.^30–38^ Furthermore, it has been found that the yield proportion increased when the amount of the catalyst increased up to 100 mol% in the reaction mixture. Herein, we optimized the reaction condition using different metal catalysts and determined that copper(ii) acetate (1 equiv.) in DMSO (1 mL) provided the standardized conditions for C–H selenylation to the substrate (2a) (Scheme 1).

**Scheme 2:**
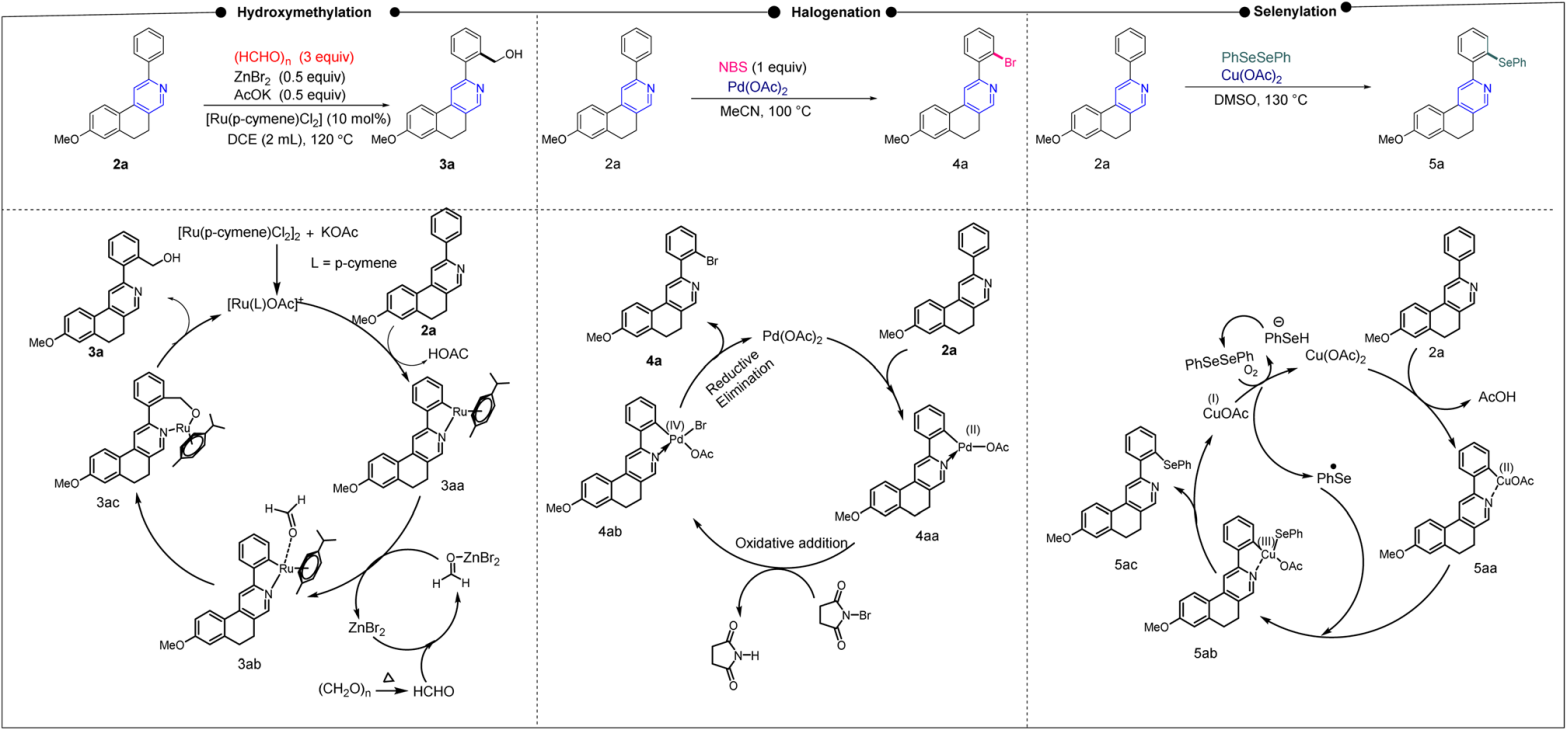
Plausible mechanism of transition metal-catalyzed C–H functionalization.

**Fig. 3:**
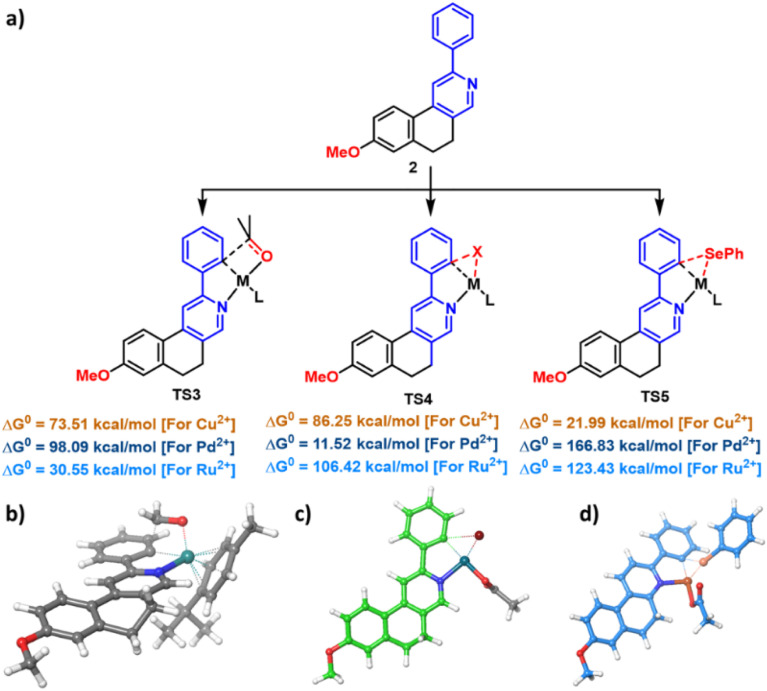
Energetically favourable transition state in a computational mechanistic study. (a) A schematic presentation of the energy of three probable transition states of the reaction under investigation with the metal ions of Cu, Pd, and Ru. The most energy-minimised transition state structure of (b) Ru(ii) ion in hydroxymethylation reaction, (c) Pd(ii) in bromination reaction, and (d) Cu(ii) in selenylation reaction.

After establishing diverse C–H functionalizations of the 2-arylbenzo[*f*]isoquinolines, we investigated the reaction mechanism (Scheme 2) for our synthesized scaffolds based on a literature report, which was confirmed by density functional theory (DFT) calculations. In the hydroxymethylation reaction, Ru(ii) activates *ortho* to the 2-aryl part of isoquinoline by coordinating Ru(ii) with the N-atom, followed by coordination of the formyl group, and subsequently the hydroxymethylation, leading to the desired product (3a). The same substrate undergoes a halogenation reaction in the presence of Pd(ii), where the activation of the 2-aryl ring occurs in the same fashion as that of Ru(ii); however, the oxidative addition, followed by the reductive elimination, leads to the desired halogenation product. A similar *ortho* C–H bond of 2-arylbenzo[*f*]isoquinoline was activated by copper(ii) for the selenylation reaction. In this case, Cu(ii) is oxidized by the PhSe radical; then, reductive elimination affords the targeted product along with the copper(i) complex, which undergoes areal oxidation to the copper(ii) complex and maintains the catalytic cycle. After establishing the mechanism *via* a series of controlled experiments, we emphasize the reaction pathways through density functional theory, as this study offers the best opportunity to understand the reaction dynamics (Fig. 3).

Density functional theory (DFT)^44^ is employed to investigate the metal selectivity in the regioselective halogenation, hydroxymethylation, and selenylation reactions (Fig. 3). It is calculated using Gaussian 09 with the mPW1PW91 functional. Light atoms (H, C, O, Se, and N) employed the D95V basis, while Cu, Pd, and Ru were treated with LANL2DZ to include relativistic effects. Structures were optimized in singlet and triplet states, and frequency analyses verified all geometries as true minima, with no imaginary modes detected.^39–41^ Specifically, the transition state (TS) for regioselective hydroxymethylation across various metal complexes, including Cu, Pd, and Ru, was calculated. This reaction proceeds through a four-membered transition state (TS3), in which the oxygen of formaldehyde coordinates with the metal ion, and the M−C bond facilitates a nucleophilic attack on the carbon of formaldehyde. Among the metal complexes studied, the Ru-complex exhibits the lowest free energy barrier for reaching the hydroxymethylation transition state, with a Δ*G*^‡^ value of 30.55 kcal mol^−1^ (Fig. 3a). In contrast, the Cu-complex has a significantly higher Δ*G*^‡^ value of 73.51 kcal mol^−1^, and the Pd-complex is higher at 98.09 kcal mol^−1^. These elevated free energy changes for the transition state formation in the Cu and Pd complexes indicate disfavorability toward achieving the transition state.

Ruthenium, particularly in its Ru-*p*-Cymene, adopts a pseudo-octahedral geometry that provides greater spatial flexibility for substrate coordination and transition state stabilisation (Fig. 3b). This flexibility allows formaldehyde to align optimally for nucleophilic attack, minimising steric hindrance and enabling a lower activation barrier. In contrast, Cu(ii) and Pd(ii) typically favour square planar geometries, which restrict the approach of formaldehyde and hinder the formation of the four-membered TS3 ring. This geometric constraint leads to significantly higher energy barriers, making the transition state energetically unfavourable. Similar findings have been reported in theoretical studies of regioselective C–H functionalization, where square planar geometries often limit reactivity due to poor orbital overlap and steric congestion. Moreover, the role of *p*-Cymene in stabilising transition states and enhancing regioselectivity is highlighted in Ru-catalysed annulation reactions.

In halogenation and selenylation reactions, a nucleophilic attack occurs on the metal-coordinated halogen, such as bromide, and on the selenium atom. Both reactions can involve a three-membered transition state (TS). Density functional theory (DFT) results reveal that the palladium (Pd) ion exhibits a relatively low activation energy, with a Gibbs free energy of activation (Δ*G*^‡^) of 11.52 kcal mol^−1^ for transition state TS4 during bromination (Fig. 3a).

For the palladium complex, TS4 adopts a co-planar geometry in which all reacting atoms align with the square planar arrangement characteristic of the metal. In contrast, the Cu and Ru complexes display different behaviours. In the case of the Cu complex, TS4 is destabilized by an unstable Cu–C bond, which has a significant bond length of 2.88 Å, leading to a non-planar configuration. This distortion results in a substantially higher energy transition state, with an activation energy of 86.25 kcal mol^−1^. Meanwhile, the Ru complex experiences destabilization in a tight transition state due to its non-planar structure, resulting in a much higher activation energy of 106.42 kcal mol^−1^.

In the context of the selenylation reaction outlined in Fig. 3a, the transition state (TS5) involving the three-membered structure with a phenyl ring attached to Se and coordinated to a Ru complex is characterized by a high energy barrier, with a Δ*G*^‡^ of 123.43 kcal mol^−1^. For the Pd complex, the square planar geometry of the metal centre poses challenges during the reaction, particularly when attempting to accommodate the bulky Se-phenyl group. This rigidity contributes to the elevated activation energy of Δ*G*^‡^ = 166.83 kcal mol^−1^. In contrast, the Cu complex exhibits greater flexibility in its geometry. It can transition from a square planar to a tetrahedral geometry, effectively minimising the steric hindrance associated with bulky SePh. This adaptability requires a significantly lower activation energy of Δ*G*^‡^ = 21.99 kcal mol^−1^ for the selenylation reaction.

**Fig. 4:**
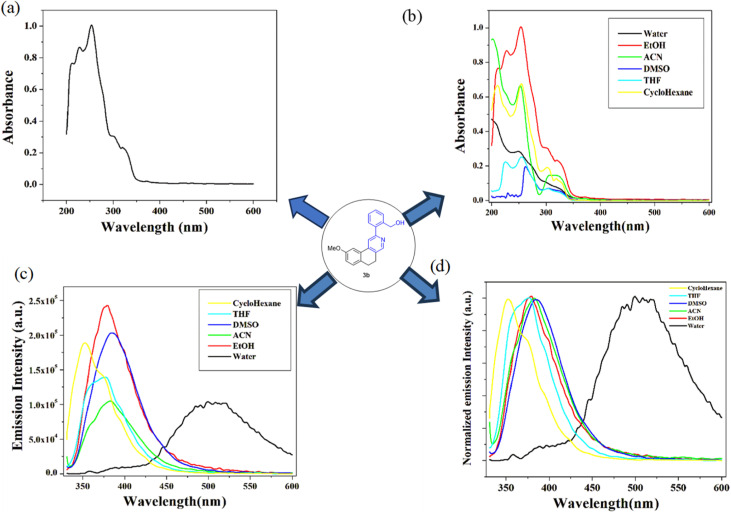
Steady state absorption spectra of (3b) (a) in EtOH solution and (b) in different solvents (1.85 × 10^−5^ M). Steady state emission spectra of (3b) (2 × 10^−6^ M) (c) in different solvents and (d) normalized emission spectra of the compound in different solvents.

Once detailed synthetic studies were completed, we shifted the focus of our research to the photophysical studies of our synthesized compounds owing to their fluorescent nature. In a recent report in our laboratory, it was found that the –OMe-substituted isoquinoline exhibited good quantum yield; hence, we selected the substrate hydroxymethyl derivative of 2b for the photophysical study.^28^ The new fluorescent probe (3b) showed a significant solvatochromic effect, and the highest emission intensity was noticed in acetonitrile (ACN) solution. Accordingly, in this study, we investigated the steady state absorption–emission properties of the synthesized compound in different solvents of varying polarity and measured their quantum yields. The absorption spectra in different solvents of varying polarity show absorption in the range of 240–350 nm, with absorption maxima (*λ*_max_) in the range of 250–255 nm [Fig. 4b]. However, in almost all the solvents, there are two additional absorption maxima in the ranges of 300–305 nm and 320–325 nm [Fig. 4a and b].

These three different absorption maxima can be attributed to electronic transitions from the ground state (*S*_0_) to higher excited states (*S*_n_). The absorption maxima at the 320–325 nm range, which has the highest wavelength, can be attributed to the lowest energy transition, *i.e.*, *S*_0_ to *S*_1_. Similarly, the absorption maxima at 300 to 305 nm may correspond to *S*_0_ to *S*_2_, and the one at 250 to 255 nm corresponds to *S*_0_ to *S*_3_. Upon excitation, the synthesised compound (3b) in different solvents showed emission in the range of 360–400 nm, with the highest emission intensity among the studied solvents in ACN solution (*Ф* = 0.22) (Table 4).i
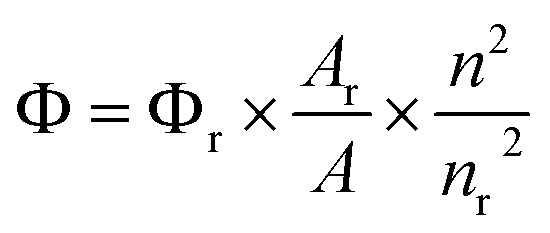
In eqn (i), ref. (42) *Φ* is the quantum yield of the sample, *A* is the absorbance at the excitation wavelength, *I* is the integrated intensity of the emission spectra, *n* is the refractive index, and the reference fluorophore with a known quantum yield (*Φ*_r_) (α-naphthol is taken as a reference for quantum yield calculations)^42^ is denoted by the subscript *r*.

**Table 4:** Steady state spectroscopic parameters of 3b obtained from band maxima of absorption (*λ*_abs_) and emission (*λ*_em_) spectra and quantum yield. Fluorescence quantum yield is measured with respect to the literature standards of 3b (ref. 42)

Solvent	*λ*_abs_[nm]	*λ*_em_[nm]	Molar extinction coefficient (*ϵ*) [mol^−1^ cm^−1^]	Quantum yield
Water	320	505	4108	0.0148
ACN	320	382	7837	0.2215
EtOH	301, 320	380	12 702	0.1729
THF	320	374	2756	0.0039
Cyclohexane	301, 320	353	6756	0.0119
DMSO	320	383	3189	0.0063

To calculate the quantum yield, we use the relative method. In this study, α-naphthol is chosen as the reference due to its well-established photophysical properties and reliable quantum yield values. We use equation (i), which provides a quantitative measure of the efficiency of photon emission relative to photon absorption, where the excitation wavelength is 320 nm. Based on our analysis compound in the ACN solution, sample (3b) exhibited the highest quantum yield among the tested samples. This indicates that the sample in the ACN has the most efficient radiative decay pathway, which suggests its potential suitability for applications requiring high fluorescence efficiency. From Fig. 4, it is observed that the emission spectra of the compound are red shifted as the polarity of the solvent increases. This shift suggests a solvatochromic effect, in which the molecular environment influences the electronic transitions of the system by the solvent polarity. The increased solvent polarity of the solvent likely stabilizes the excited state more than the ground state, which leads to a redshift in the emission spectrum. This behavior is commonly observed in molecules with significant charge redistribution upon excitation, which indicates strong solvent–solute interactions.

**Fig. 5:**
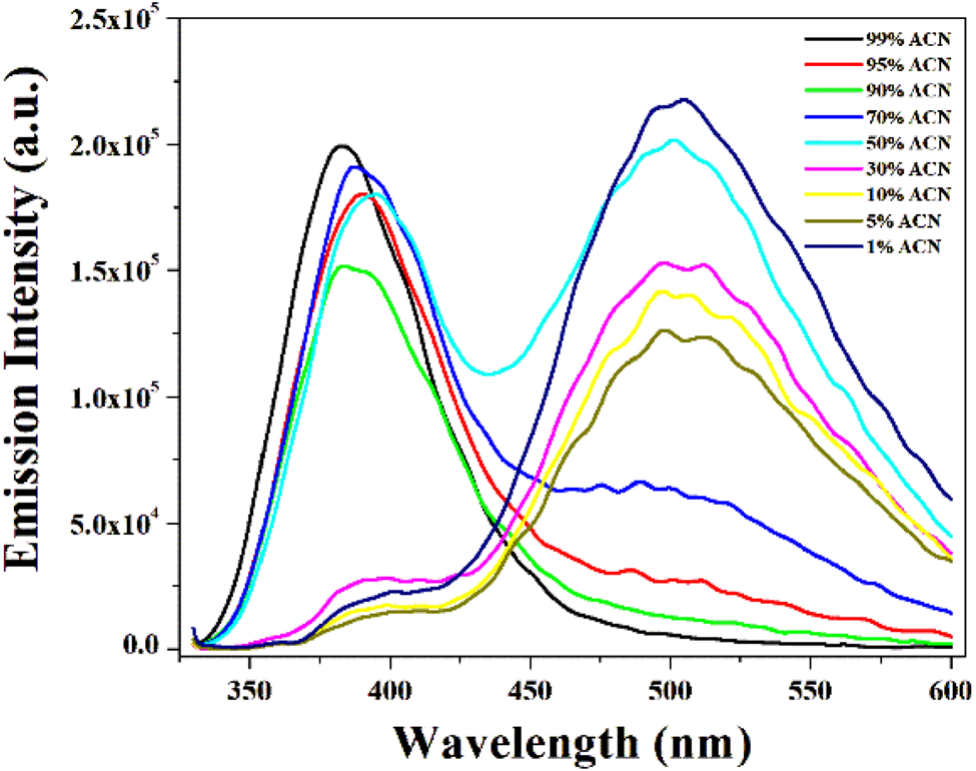
Emission spectra of the compound in the water–ACN solution upon the gradual addition of water.

Interestingly, as depicted in (Fig. 4c), we already observe that the emission spectra of the compound shift toward the right as the polarity of the solvent increases, but there is a remarkable red shift shown in the case of water solution. This observation suggests that water influences the electronic environment of the fluorophore differently than other solvents. This may be due to its high polarity, hydrogen bonding ability and high dielectric constant. The huge red shifted fluorescence is due to aggregation-induced emission (AIE). A similar type of observation has also been found in other reported molecules.^43^ To confirm this, we add water to the ACN solution [Fig. 5]. Initially, there is only one peak at 380 nm in the ACN solution, but when the water percentage increases, the emission peak at 380 nm gradually decreases; concomitantly, the peak at 505 nm increases. This indicates a significant spectral shift. This trend suggests that water-induced molecular interactions may be due to aggregation effects. However, it is also observed that the absorption spectra in water are almost the same in other solvents. Such spectral behavior confirms the presence and influence of water and highlights molecular aggregation in the excitation state.

**Fig. 6:**
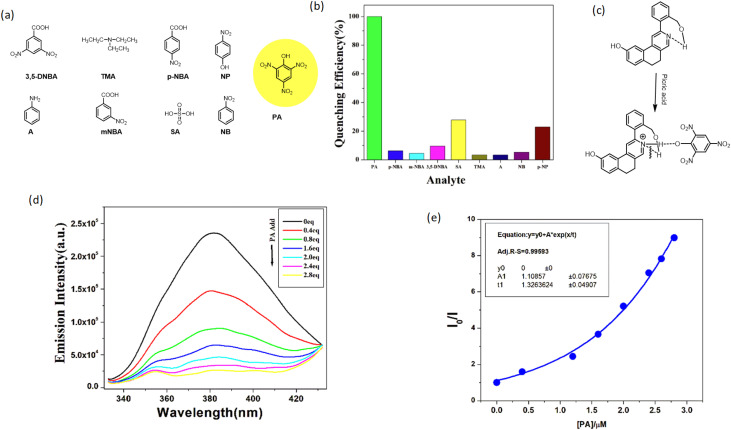
Molecular sensing properties of the synthesized compound. (a) Different toxic analytes. (b) Comparison of quenching efficiency. (c) Structural explanation for quenching. (d) Fluorescence titration curves upon gradual addition of PA and (e) the corresponding Stern–volmer plot *I*_0_/*I vs.* [PA] in ACN.

After successful observation of the solvent-induced fluorescent study of new fluorophore 3b, we turn our research focus towards toxic organic molecule sensing. In recent years, we have published several articles on toxic molecule sensing with our new synthetic fluorophore and found interesting observations. Consequently, we want to explore this observation according to the functional group present in the fluorophore. Our fluorophore has the –CH_2_OH group and pyridinyl N-atom, which have a greater tendency to capture labile protons. Hence, we take several aromatic acids (3,4-dinitrobenzoic acid, *p*-nitrobenzoic acid, sulphonic acid and *p*-nitrophenol) along with amine derivatives (triethylamine and aniline). As illustrated in Fig. 6d, we can infer that the fluorescence quenching is achieved only in the presence of picric acid (∼99%) upon adding 2.8 equiv.of picric acid in 1 equiv.solution of synthesized compound 3d. Among the rest used quenchers, sulphuric acid showed 20–25% emission quenching, while the others showed negligible quenching (<10%). Thus, the synthesized compound displayed high selectivity towards picric acid compared to other selected quenchers. However, with the increasing concentration of picric acid, a new adsorption peak emerges at approximately 370 nm, and its intensity progressively increases with the further addition of picric acid (Fig. S2).^44^ To determine the quenching constant, a solution of picric acid (20 µM) was gradually added to a solution of our compound (1 µM), and the change in emission was recorded. Using the recorded data, we plotted a graph of the fluorescence intensity ratio (*I*_O_/*I*) against the concentration of PA, which resulted in a nonlinear curve [Fig. 6e]. Therefore, we put the data in equation *I*_O_/*I* = *A*e^*k*|*Q*|^ + *B* for the exponential fitting of the quenching. From this nonlinear curve fitting, the quenching constant was calculated to be around 7.54 × 10^5^ M^−1^, and the LOD was 0.29 µM (Fig. S1).^44^

To better understand the quenching mechanism, the absorption of PA and emission of the compound were plotted. It is important to mention that in the case of static quenching, a non-fluorescent complex is formed owing to the binding of the quencher with the sensor in the ground state (Fig. 6). The higher quenching efficiency of PA can be explained by the higher efficiency of proton donating capability to the basic nitrogen of the compound. The H-bonding capability of phenolic analytes becomes more pronounced if electron-withdrawing groups, such as –NO_2_, are present. The presence of three –NO_2_ groups in picric acid makes the phenolic proton more labile. Thus, PA showed the highest quenching efficiency.

## Conclusion

In summary, we developed a novel synthetic route of hydroxymethylation (–CH_2_OH) of fused isoquinoline derivatives *via* Ru(ii)-catalyzed functional group-directed C–H activation, which is the key analogue of Decumbenine B. This protocol was extended to regioselective halogenation (–Cl and –Br), and selenylation (–SePh) reactions *via* metal-catalyzed Pd(ii) or Cu(ii) promoted C–H activation in the fused isoquinoline moieties. DFT studies were conducted to gain insights into the mechanisms of metal-catalyzed selective C–H activation reactions. Interestingly, the presence of a –CH_2_OH functional group in the newly synthesized Decumbenine B analogues exhibited aggregation-induced emission (AIE) and enabled the selective sensing of toxic picric acid. These findings suggest a promising direction for further exploration of natural alkaloids, like Decumbenine B, as fluorescent sensors for the detection of hazardous environmental pollutants.

## Conflicts of interest

There are no conflicts to declare.

## Supplementary Material

RA-016-D5RA09256F-s001

## Data Availability

The data supporting this article have been included as part of the Supplementary information (SI). Supplementary information: experimental details, characterization and DFT data. See DOI: https://doi.org/10.1039/d5ra09256f.
