# Supplementary material for: Directing group-assisted C–H functionalization of 2-arylbenzo[f]isoquinoline: a robust route to decumbenine B analogues with photophysical applications
Source: RSC Adv. 2026 Jan 5;16(2):1310–21. doi: 10.1039/d5ra09256f (PMC12767574; doi:10.1039/d5ra09256f)
Supplement: RA-016-D5RA09256F-s001 [file RA-016-D5RA09256F-s001.pdf]

Supplementary Information (SI) for RSC Advances.
This journal is © The Royal Society of Chemistry 2025

# Directing Group Assisted C–H Functionalization of 2-Arylbenzo[f]isoquinoline: A Robust Route to Decumbenine B Analogues with Photophysical Applications

Sampa Mondal,[a,b]≠ Sudipta Ghara,[a]≠ Sk Abulkalam Azad,[a] Paritosh Barik,[a] Prasenjit Sen,[c] Nayim Sepay,[d] Prasanta Patra,[e] Subrata Mahanta,*[c] Laksmikanta Adak,*[b] and Shubhankar Samanta*[a]

≠ These authors contributed equally to this work

[a] S. Mondal, S. Ghara, Sk. A. Azad, P. Barik, Dr. S. Samanta
Department of Chemistry,
EB 2, Sector I, Salt Lake, Kolkata-700064, West Bengal, India
E-mail: chemshubha@gmail.com

[b] S. Mondal, Dr. L. Adak
Department of Chemistry
Indian Institute of Engineering Science and Technology, Shibpur
Botanic Garden, Howrah 711103

[c] P. Sen, Dr. S. Mahanta
Department of Chemistry
National Institute of Technology Jamshedpur, India

[d] N. Sepay
Department of Chemistry
Lady Brabourne College
Kolkata-700017, West Bengal, India

[e] P. Patra
Department of Chemistry
Jhargram Raj College, Jhargram, India

## Table of Contents

1. General information S2
2. General experimental procedure for C-H activation reactions and characterization data of products S3
3. $^{1}$H and $^{13}$C NMR Spectra S10
4. DFT calculation S30
5. The limit of detection (LOD) S54
6. Absorption spectra toward picric acid sensing S55

## 1. General information:

All reactions dealing with air- or moisture-sensitive compounds were carried out in a dry reaction vessel. The air- and moisture-sensitive liquids and solutions were transferred via syringes or a PTFE cannula. Precoated silica gel 60 F254 TLC sheets (Merck) were used to monitor the reaction. The spots on TLC were visualized under UV 254 nm light. For purification in column chromatography 60-120 or 100-200 mesh silica gels (Sprectrochem Co.) were used. n-Hexane (Merck) or petroleum ether (boiling range 60-80 °C) and ethyl acetate eluent were used in column chromatographic separation.

### Instrumentation

*NMR spectroscopy*

$^{1}H$ and $^{13}C$ NMR spectra of all the synthesized compounds were recorded in 300 MHz, 400 MHz, and 700 MHz NMR spectrometers (Bruker Avance-300, Bruker Avance-400, and Bruker Avance-700) using $CDCl_3$ and DMSO-$d_6$ (99.9 atom% D) as a solvent with TMS as internal standard or without TMS. The proton chemical shift values are reported in parts per million (ppm, δ scale) downfield from tetramethyl silane (TMS) and are referenced to TMS (δ 0.0), $CHCl_3$ (δ 7.26), and DMSO (δ = 2.50). The chemical shifts of the carbon atoms are reported in parts per million (ppm, δ scale) downfield from TMS and referenced to the carbon resonance of $CDCl_3$ (δ 77.16), DMSO-$d_6$ (δ 39.52) The data are presented as chemical shift, multiplicity (s = singlet, d = doublet, dd= doublet of doublet, t = triplet, q = quartet, quint = quintet, sext = sextet, sept = septet, m = multiplate and/or multiple resonances, and br = broad), coupling constant in hertz (Hz), and signal area integration in natural numbers.

*HRMS*

High-resolution mass spectra (HRMS) were recorded using fast atom bombardment (FAB) ionization with a Q-TOF mass spectrometer.

### Materials

All chemical and solvent were purchased from Sigma-Aldrich Co., Tokyo Chemical Industry (India) Pvt. Ltd., Spectrochem Pvt. Ltd., Sisco Research Laboratory Pvt. Ltd., Thermo Fisher Scientific, BLD pharm, etc. [Ru(*p*-cymene)$Cl_2$]$_2$ and $Pd(OAc)_2$ was purchased from bld pharm, $Cu(OAc)_2$ was purchased from Sigma-Aldrich .

## 2. General experimental procedure for C–H of activation reaction and characterization data of products:

### General experimental procedure A: general experimental procedure of C–H activation of 2-arylbenzo(f)isoquinoline:

2-arylbenzo(f)isoquinoline (0.1 mmol), paraformaldehyde (0.3 mmol, 3 equiv.), $ZnBr_2$ (0.05 mmol, 0.5 equiv.) and AcOK (0.05 mmol, 0.5 equiv.), [Ru(*p*-cymene)$Cl_2$]$_2$ (10 mol%) and 2 mL of DCE, were taken in an oven-dried reaction vessel. The mixture was heated at 120 °C for 3h under nitrogen atmosphere. Reaction progress was monitored by TLC. Upon completion, the reaction mixture was cooled and $CH_2Cl_2$ was evaporated under reduced pressure, and $H_2O$ was added. The aqueous phase was extracted with EtOH, the combined organic phases were extracted with brine, and dried with $Na_2SO_4$. Evaporation of the solvent. The crude product was purified by column chromatography on silica gel (100–200 mesh) using a hexane (or petroleum ether) and ethyl acetate mixture as the eluent to get the pure product.

### General experimental procedure B: general experimental procedure for bromination:

2-arylbenzo(f)isoquinoline (0.1 mmol), $Pd(OAc)_2$ (10 mol%), and NBS (0.1 mmol,1 equiv.) in 2 mL acetonitrile were taken in an oven-dried reaction vessel. The mixture was heated at 100 °C for 3h under a nitrogen atmosphere. Reaction progress was monitored by TLC. Upon completion, the reaction mixture was cooled, and $H_2O$ was added. The aqueous phase was extracted with EtOH, the combined organic phases were extracted with brine, and dried with $Na_2SO_4$. Evaporation of the solvent. The crude product was purified by column chromatography on silica gel (100–200 mesh) using a hexane (or petroleum ether) and ethyl acetate mixture as the eluent to get the pure product.

### General experimental procedure C: general experimental procedure for Selenylation:

2-arylbenzo(f)isoquinoline (0.1 mmol), $Cu(OAc)_2$ (0.1 mmol,1 equiv.) and NBS (0.1 mmol,1 equiv.) in 1 mL DMSO were taken in an oven-dried reaction vessel. The mixture was heated at 150 °C for 6h under a nitrogen atmosphere. Reaction progress was monitored by TLC. Upon completion, the reaction mixture was cooled, and $H_2O$ was added. The aqueous phase was extracted with EtOH, the combined organic phases were extracted with brine, and dried with $Na_2SO_4$. Evaporation of the solvent. The crude product was purified by column chromatography on silica gel (100–200 mesh) using a hexane (or petroleum ether) and ethyl acetate mixture as the eluent to get the pure product.

**(2-(8-methoxy-5,6-dihydrobenzo[f]isoquinolin-2-yl)phenyl)methanol (3a):**

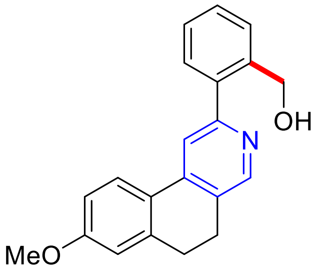


The reaction was carried out according to the general procedure A. 27 mg (85% yield), brown semi-liquid. $^1$H NMR (300 MHz, $CDCl_3$) δ in ppm 8.44 (s, 1H), 7.82 – 7.78 (m, 2H), 7.63 – 7.59 (m, 1H), 7.51 – 7.48 (m, 1H), 7.44 – 7.39 (m, 2H), 6.90 (dd, *J* = 8.6, 2.7 Hz, 1H), 6.84 (d, *J* = 2.7 Hz, 1H), 4.50 (s, 2H), 3.87 (s, 3H), 2.93 (s, 4H). $^{13}$C NMR (75 MHz, $CDCl_3$) δ 158.0, 155.2, 144.1, 140.4, 137.6, 137.4, 137.4, 128.2, 127.1, 126.9, 126.1, 125.2, 123.0, 121.6, 114.2, 111.0, 110.0, 61.9, 52.5, 25.93, 22.3. HRMS (EI$^+$) m/z $[M+H]^+$ Calcd for $C_{21}H_{20}NO_2$: 318.1489, Found 318.1487.

**(2-(9-methoxy-5,6-dihydrobenzo[f]isoquinolin-2-yl)phenyl)methanol (3b):**

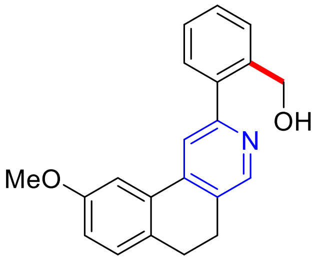


The reaction was carried out according to the general procedure A. 26 mg, 81% yield), brown semi-liquid.$^1$H NMR (300 MHz, $CDCl_3$) δ 8.49 (s, 1H), 7.86 (s, 1H), 7.64 – 7.61 (m, 1H), 7.52 – 7.49 (m, 1H), 7.46 – 7.42 (m, 2H), 7.38 (d, *J* = 2.6 Hz, 1H), 7.24 (d, *J* = 8.3 Hz, 1H), 6.93 (dd, *J* = 8.3, 2.6 Hz, 1H). 4.50 (s, 2H), 3.87 (s, 3H), 2.91 (s, 4H). $^{13}$C NMR (75 MHz, $CDCl_3$) δ 159.1, 158.3, 147.4, 143.4, 140.4, 132.7, 131.2, 130.9, 130.6, 130.1, 129.7, 129.2, 128.3, 117.9, 115.3, 113.5, 110.1, 64.8, 55.6, 27.6, 25.5. HRMS (EI$^+$) m/z $[M+H]^+$ Calcd for $C_{21}H_{20}NO_2$: 318.1489, Found 318.1483.

**(2-(5,6-dihydrobenzo[f]isoquinolin-2-yl)phenyl)methanol (3c):**

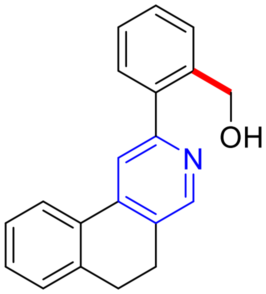


The reaction was carried out according to the general procedure A. 19 mg, 67% yield), brown semi-liquid. $^1$H NMR (400 MHz, $CDCl_3$) δ 8.50 (s, 1H), 7.91 – 7.81 (m, 2H), 7.64 – 7.60 (m, 1H), 7.52 – 7.30 (m, 6H), 4.50 (s, 2H), 3.00 - 2.93 (m, 4H). $^{13}$C NMR (101 MHz, $CDCl_3$) δ 158.3, 147.4, 143.4, 140.5, 140.4, 138.5, 131.8, 131.3, 130.7, 130.1, 130.0, 129.2, 128.9, 128.3, 127.5, 124.5, 117.9, 64.9, 28.5, 25.2. HRMS (EI$^+$) m/z $[M+H]^+$ Calcd for $C_{20}H_{18}NO$: 288.1383, Found 288.1378.

**(2-(6-methyl-5,6-dihydrobenzo[f]isoquinolin-2-yl)phenyl)methanol (3d):**

The reaction was carried out according to the general procedure A. 24 mg (80% yield), brown

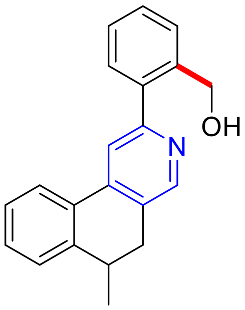


semi-liquid. $^1$H NMR (300 MHz, $CDCl_3$) δ 8.48 (s, 1H), 7.92 – 7.86 (m, 2H), 7.65 – 7.62 (m, 1H), 7.52 – 7.34 (m, 1H), 7.45 – 7.34 (m, 5H), 4.51 (s, 2H), 3.19 – 3.05 (m, 2H), 2.77 (dd, *J* = 15.0, 6.1 Hz, 1H), 1.26 (s, 3H). $^{13}$C NMR (75 MHz, $CDCl_3$) δ 158.3, 147.9, 143.3, 143.0, 140.5, 140.4, 131.2, 130.9, 130.3, 130.1, 129.4, 129.2, 128.3, 127.4, 127.3, 124.7, 117.7, 64.9, 32.7, 32.6, 20.1. HRMS (EI$^+$) m/z $[M+H]^+$ Calcd for $C_{21}H_{20}NO$: 302.1539, Found 302.1543

**(5-fluoro-2-(9-methoxy-5,6-dihydrobenzo[f]isoquinolin-2-yl)phenyl)methanol (3e):**

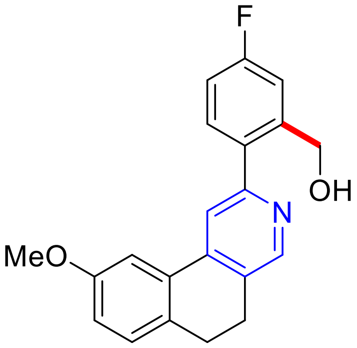


The reaction was carried out according to the general procedure A. 24 mg, (72% yield), brown semi-liquid. $^{1}$H NMR (300 MHz, $CDCl_3$) δ 8.48 (s, 1H), 7.80 (s, 1H), 7.59 (dd, *J* = 8.5, 5.6 Hz, 1H), 7.37 (d, *J* = 2.6 Hz, 1H), 7.23 – 7.09 (m, 3H), 6.94 (dd, *J* = 8.3, 2.6 Hz, 1H), 4.46 (s, 2H), 3.88 (s, 3H), 2.91 (s, 4H). $^{13}$C NMR (75 MHz, $CDCl_3$) δ 162.97 (d, *J* = 249.3 Hz), 159.1, 157.4, 147.5, 143.5, 142.89 (d, *J* = 6.8 Hz), 136.50 (d, *J* = 3.3 Hz), 132.6, 131.86 (d, *J* = 8.4 Hz), 130.84 (d, *J* = 27.9 Hz), 129.7, 118.1, 117.8, 117.7 115.4, 114.94 (d, *J* = 21.3 Hz), 110.2, 64.5, 55.7, 27.6, 25.6. HRMS (EI$^{+}$) m/z [M+H]$^{+}$ Calcd for $C_{21}H_{19}FNO_2$: 336.1394, Found 336.1393.

**(2-(7-methoxy-5,6-dihydrobenzo[f]isoquinolin-2-yl)-4-methylphenyl)methanol (3f):**

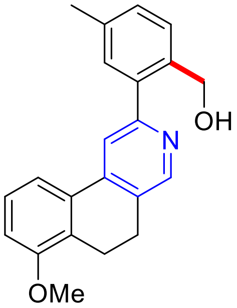


The reaction was carried out according to the general procedure A. 23 mg (71% yield), brown semi-liquid. $^{1}$H NMR (400 MHz, $CDCl_3$) δ 8.48 (s, 1H), 7.88 (s, 1H), 7.53 – 7.50 (m, 1H), 7.42 – 7.32 (m, 3H), 7.23 (dd, *J* = 7.7, 1.9 Hz, 1H), 6.97 (dd, *J* = 8.2, 0.9 Hz, 1H), 4.46 (s, 2H), 3.90 (s, 3H), 3.00 – 2.95 (m, 2H), 2.92 – 2.87 (m, 2H), 2.43 (s, 3H). $^{13}$C NMR (101 MHz, $CDCl_3$) δ 158.31, 156.87, 147.28, 143.37, 140.43, 137.94, 137.63, 133.01, 131.20, 130.80, 130.66, 129.77, 127.64, 127.08, 118.18, 116.78, 111.72, 64.52, 55.80, 24.63, 21.37, 20.34. HRMS (EI$^{+}$) m/z [M+H]$^{+}$ Calcd for $C_{22}H_{22}NO_2$: 332.1645, Found 332.1649.

**(2-(8-methoxy-5,6-dihydrobenzo[f]isoquinolin-2-yl)-5-methylphenyl)methanol (3g):**

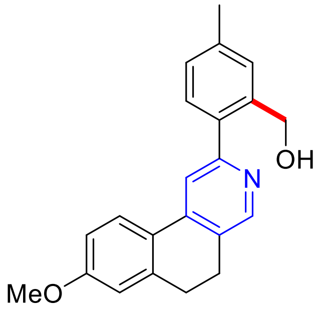


The reaction was carried out according to the general procedure A. 27 mg (80% yield), brown semi-liquid. $^{1}$H NMR (300 MHz, $CDCl_3$) δ 8.41 (s, 1H), 7.78 (d, *J* = 8.2 Hz, 2H), 7.51 (d, *J* = 7.8 Hz, 1H), 7.31 (d, *J* = 1.8 Hz, 1H), 7.25 – 7.21 (m, 1H), 6.91 – 6.82 (m, 2H), 4.47 (s, 2H), 3.86 (s, 3H), 2.91 (s, 4H), 2.42 (s, 3H). $^{13}$C NMR (75 MHz, $CDCl_3$) δ 160.9, 158.1, 147.0, 143.2, 140.3, 140.2, 139.0, 137.6, 131.9, 129.9, 129.5, 128.8, 126.0, 124.6, 116.9, 113.9, 113.0, 64.9, 55.4, 28.9, 25.2, 21.2. HRMS (EI$^{+}$) m/z [M+H]$^{+}$ Calcd for $C_{22}H_{22}NO_2$: 332.1645, Found 332.1639.

**(5-methyl-2-(6-methyl-5,6-dihydrobenzo[f]isoquinolin-2-yl)phenyl)methanol (3h):**

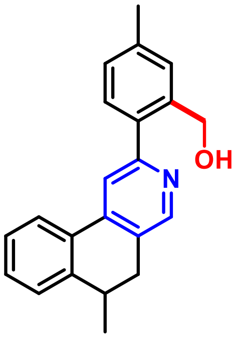


The reaction was carried out according to the general procedure A. (19 mg, 61% yield), yielding a brown semi-liquid. $^{1}$H NMR (300 MHz, $CDCl_3$) δ 8.46 (s, 1H), 7.91 – 7.84 (m, 2H), 7.53 (d, *J* = 7.8 Hz, 1H), 7.43–7.22 (m, 5H), 4.48 (s, 2H), 3.17 – 3.02 (m, 2H), 2.75 (dd, *J* = 15.1, 6.2 Hz, 1H), 2.42 (s, 3H), 2.04 (s, 3H). $^{13}$C NMR (75 MHz, $CDCl_3$) δ 171.3, 158.2, 147.8, 143.3, 143.0, 140.2, 139.2, 137.5, 132.0, 130.9, 130.3, 130.0, 129.1, 128.9, 127.4, 124.6, 117.4, 64.8, 60.5, 21.2, 20.0, 14.3. HRMS (EI$^{+}$) m/z [M+H]$^{+}$ Calcd for $C_{22}H_{22}NO$ : 316.1696, Found 316.1700.

**(2-(9-bromo-5,6-dihydrobenzo[f]isoquinolin-2-yl)-5-methylphenyl)methanol (3i)**

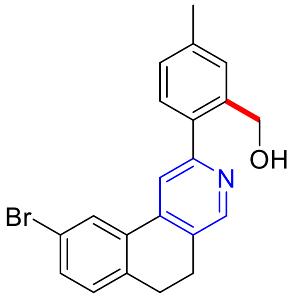


The reaction was carried out according to the general procedure A. 24 mg (65% yield), brown semi-liquid. $^{1}$H NMR (400 MHz, $CDCl_3$) δ 8.52 (s, 1H), 7.97 (d, $J$ = 2.0 Hz, 1H), 7.85 (s, 1H), 7.65 – 7.61 (m, 1H), 7.52 – 7.43 (m, 3H), 7.20 (d, $J$ = 8.1 Hz, 1H), 4.49 (s, 2H), 2.94 (s, 4H), 2.04 (s, 3H). $^{13}$C NMR (101 MHz, $CDCl_3$) δ 158.6, 147.6, 142.2, 140.4, 140.1, 137.2, 133.9, 132.7, 131.3, 130.6, 130.5, 130.1, 129.4, 128.4, 127.5, 121.2, 117.9, 64.8, 29.8, 28.0, 25.0.

**(2-(9-methoxy-5,6-dihydrobenzo[f]isoquinolin-2-yl)naphthalen-1-yl)methanol (3j):**

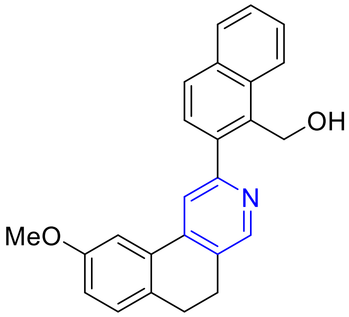


The reaction was carried out according to the general procedure A. 24 mg (65% yield), brown semi-liquid.$^{1}$H NMR (300 MHz, $CDCl_3$) δ 8.52 (s, 1H), 8.08 (s, 1H), 8.00 – 7.87 (m, 4H), 7.58 – 7.51 (m, 2H), 7.44 (d, $J$ = 2.6 Hz, 1H), 7.23 (s, 1H), 6.94 (dd, $J$ = 8.3, 2.6 Hz, 1H), 4.67 (s, 2H), 3.88 (s, 3H), 2.92 (s, 4H). $^{13}$C NMR (75 MHz, $CDCl_3$) δ 159.1, 158.4, 147.4, 143.5, 138.6, 137.9, 133.5, 132.9, 132.7, 131.0, 130.6, 130.1, 129.8, 129.7, 128.0, 127.8, 126.9, 126.6, 118.2, 115.2, 110.3, 65.1, 55.7, 27.6, 25.5.

**(2-(9-methoxy-5,6-dihydrobenzo[f]isoquinolin-2-yl)-5-methylphenyl)methanol (3k):**

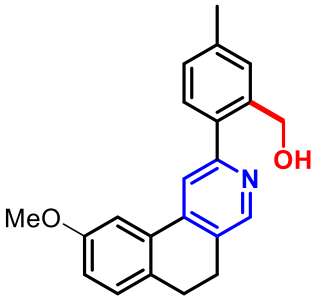


The reaction was carried out according to the general procedure A using 9-methoxy-2-(p-tolyl)-5,6-dihydrobenzo[f]isoquinoline (15 mg, 0.05 mmol). 16 mg (98% yield), brown liquid. $^{1}$H NMR (700 MHz, $CDCl_3$) δ 8.48 (s, 1H), 7.84 (s, 1H), 7.52 (d, $J$ = 7.7 Hz, 1H), 7.38 (d, $J$ = 2.6 Hz, 1H), 7.32 (s, 1H), 7.24 (dd, $J$ = 11.3, 7.4 Hz, 3H), 6.93 (dd, $J$ = 8.4, 2.6 Hz, 1H), 4.47 (s, 2H), 3.88 (s, 3H), 2.91 (s, 4H), 2.43 (s, 3H). $^{13}$C NMR (176 MHz, $CDCl_3$) δ 159.1, 158.4, 147.4, 143.3, 140.3, 139.3, 137.6, 132.9, 132.0, 130.7, 130.67, 130.1, 129.7, 128.9, 117.7, 115.3, 110.1, 64.9, 55.7, 32.1, 27.7, 25.6. HRMS ($EI^+$) m/z $[M+H]^+$ Calcd for $C_{22}H_{22}NO_2$: 332.1643, Found 332.1638.

**2-(2-bromophenyl)-8-methoxy-5,6-dihydrobenzo[f]isoquinoline (4a):**

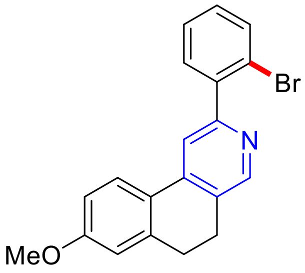


The reaction was carried out according to the general procedure B. 18 mg (51% yield) of yellowish-brown liquid. $^{1}$H NMR (400 MHz, $CDCl_3$) δ 8.56 (d, $J$ = 11.4 Hz, 1H), 7.90 – 7.81 (m, 1H), 7.79 – 7.66 (m, 3H), 7.62 – 7.54 (m, 1H), 7.49 – 7.41 (m, 1H), 6.92 – 6.83 (m, 2H), 3.89 (s, 3H), 2.97 (d, $J$ = 8.6 Hz, 4H). $^{13}$C NMR (101 MHz, $CDCl_3$) δ 148.67, 148.57, 140.23, 133.42, 132.02, 131.56, 130.57, *129.69, 127.68, 126.11, 126.06, 124.26, 118.24, 117.94, 113.99,* 112.94, 108.98, 55.53, 29.02, 25.41. HRMS ($EI^+$) m/z $[M+H]^+$ Calcd for $C_{20}H_{17}BrNO$: 366.0488, Found 366.0484.

**2-(2-bromophenyl)-5,6-dihydrobenzo[f]isoquinoline (4b):**

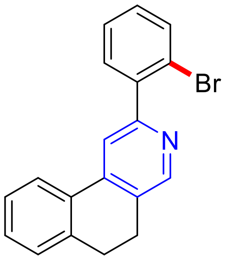


The reaction was carried out according to the general procedure B. 17 mg (52% yield) brown semi-liquid.$^{1}$H NMR (400 MHz, $CDCl_3$) δ 8.58 (s, 1H), 7.91 (s, 1H), 7.84 – 7.80 (m, 1H), 7.70 (dd, $J$ = 8.0, 1.2 Hz, 1H), 7.59 (dd, $J$ = 7.6, 1.8 Hz, 1H), 7.42 (td, $J$ = 7.5, 1.2 Hz, 1H), 7.37 – 7.34 (m, 2H), 7.31 – 7.26 (m, 1H), 7.27 (d, $J$ = 1.6 Hz, 1H), 2.98-2.94 (m, 4H). $^{13}$C NMR (101 MHz, CDCl3) δ 157.32, 148.80, 141.81, 141.55, 138.36, 133.45, 132.15, 131.57, 130.80, 129.76, 129.73, 128.73, 127.71, 127.46, 124.57, 122.11, 118.88, 28.58, 25.37. HRMS (EI$^{+}$) m/z [M+H]$^{+}$ Calcd for $C_{19}H_{15}BrN$: 336.0382, Found 336.0385.

**2-(1-bromonaphthalen-2-yl)-8-methoxy-5,6-dihydrobenzo[f]isoquinoline (4c):**

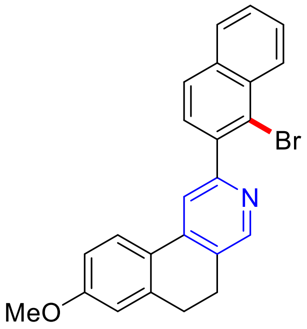


The reaction was carried out according to the general procedure B using 8-methoxy-2-(naphthalen-2-yl)-5,6-dihydrobenzo[f]isoquinoline (17 mg, 0.05 mmol), $Pd(OAc)_2$ (10 mol%), and NBS (0.05 mmol,1 equiv.). 11 mg (57% yield), brown semi-liquid. $^{1}$H NMR (300 MHz, $CDCl_3$) δ 8.52 (s, 1H), 8.08 (s, 1H), 8.01 – 7.87 (m, 4H), 7.58 – 7.50 (m, 2H), 7.44 (d, $J$ = 2.6 Hz, 1H), 7.23 (s, 1H), 6.94 (dd, $J$ = 8.3, 2.6 Hz, 1H), 4.67 (s, 2H), 3.88 (s, 3H), 2.92 (s, 4H).$^{13}$C NMR (75 MHz, $CDCl_3$) δ 156.76, 156.68, 149.08, 141.53, 139.46, 137.02, 135.35, 133.68, 129.56, 129.39, 128.83, 128.59, 127.81, 126.56, 126.44, 126.24, 125.15, 124.78, 114.39, 112.02, 110.32, 56.52, 28.96, 25.18. HRMS (EI$^{+}$) m/z [M+H]$^{+}$ Calcd for $C_{24}H_{19}BrNO$: 416.0645 and 418.0624, Found 416.0640 and 418.0628

**2-(2-bromo-4-methylphenyl)-8-methoxy-5,6-dihydrobenzo[f]isoquinoline(4d):**

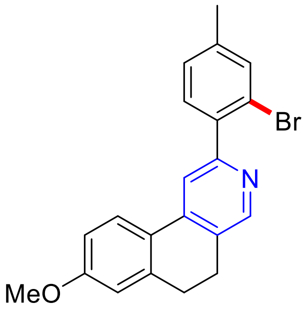


The reaction was carried out according to the general procedure B. 23 mg (60% yield), yellow semi liquid. $^{1}$H NMR (400 MHz, $CDCl_3$) δ 8.51 (s, 1H), 8.03 (s, 1H), 7.94 – 7.90 (m, 2H), 7.87 (s, 1H), 7.32 – 7.28 (m, 2H), 6.82 (s, 1H), 3.96 (s, 3H), 2.90 (s, 4H), 2.42 (s, 3H).$^{13}$C NMR (101 MHz, $CDCl_3$) δ 156.94, 156.61, 148.90, 141.34, 139.43, 138.88, 136.96, 129.61, 129.57, 129.34, 129.15, 126.85, 126.51, 113.81, 112.91, 112.00, 110.27, 56.51, 28.99, 25.15, 21.43. HRMS (EI$^{+}$) m/z [M+H]$^{+}$ Calcd for $C_{21}H_{19}BrNO$: 380.0645 and 382.0624, Found 380.0639 and 382.0619.

**2-(2-bromo-4-methylphenyl)-9-methoxy-5,6-dihydrobenzo[f]isoquinoline (4e):**

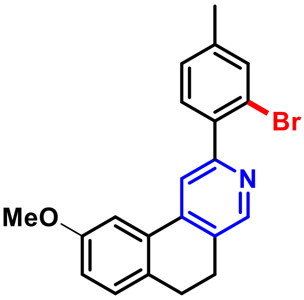


The reaction was carried out according to the general procedure A using 9-methoxy-2-(p-tolyl)-5,6-dihydrobenzo[f]isoquinoline (15 mg, 0.05 mmol). 12 mg (62% yield), brown semi-liquid.$^{1}$H NMR (700 MHz, $CDCl_3$) δ 8.60 (s, 1H), 7.55 (s, 1H), 7.48 (s, 2H), 7.31 (d, $J$ = 2.6 Hz, 1H), 7.22 (d, $J$ = 8.3 Hz, 1H), 6.91 (dd, $J$ = 8.3, 2.6 Hz, 1H), 3.85 (s, 3H), 2.93 (tt, $J$ = 7.4, 3.8 Hz, 4H), 2.38 (s, 3H). $^{13}$C NMR (176 MHz, $CDCl_3$) δ 159.1, 157.6, 148.8, 141.3, 132.9, 132.6, 131.4, 130.55, 129.6, 123.7, 119.0, 119.0, 115.5, 113.5, 109.9, 55.7, 27.6, 25.7, 20.7. HRMS ($EI^+$) m/z $[M+H]^+$ Calcd for $C_{21}H_{19}BrNO$: 380.0645, Found 380.0641.

**2-(2-chlorophenyl)-6-methyl-5,6-dihydrobenzo[f]isoquinoline (4f) :**

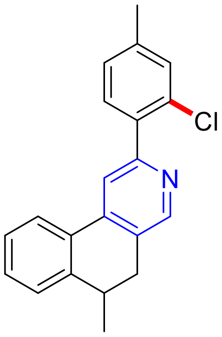


The reaction was carried out according to the general procedure B. 19 mg (62 % yield), brown semi-liquid. $^{1}$H NMR (300 MHz, CDC13) δ 8.56 (s, 1H), 7.96 (s, 1H), 7.84 – 7.81 (m, 1H), 7.55 (d, J = 7.8 Hz, 1H), 7.42 – 7.31 (m, 3H), 7.21-7.16 (m, 1H), 3.19 – 3.03 (m, 2H), 2.78 – 2.71 (m, 1H), 2.40 (s, 3H), 1.25(s,3H) $^{13}$C NMR (75 MHz, $CDCl_3$) δ 155.80, 149.33, 143.15, 141.42, 139.89, 136.57, 134.77, 131.95, 131.42, 131.26, 130.69, 129.98, 129.28, 128.01, 127.27, 124.71, 118.80, 32.89, 32.69, 21.09, 20.08. HRMS ($EI^+$) m/z $[M+H]^+$ Calcd for $C_{21}H_{19}ClN$: 320.1201, Found 320.1204.

**2-(2-chlorophenyl)-9-methoxy-5,6-dihydrobenzo[f]isoquinoline (4g):**

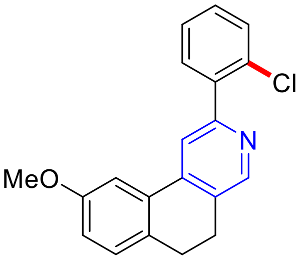


The reaction was carried out according to the general procedure B. (19 mg, 59% yield), brown semi-liquid. $^{1}$H NMR (400 MHz, $CDCl_3$) δ 8.59 (s, 1H), 7.92 (s, 1H), 7.65 (dd, $J$ = 7.3, 2.1 Hz, 1H), 7.51 (dd, $J$ = 7.6, 1.7 Hz, 1H), 7.42 – 7.33 (m, 3H), 7.22 (d, $J$ = 8.3 Hz, 1H), 6.92 (dd, $J$ = 8.3, 2.6 Hz, 1H), 3.86 (s, 3H), 2.92 (s, 4H). $^{13}$C NMR (101 MHz, $CDCl_3$) δ 159.07, 155.66, 148.69, 132.97, 132.39, 131.65, 131.11, 130.61, 130.29, 129.73, 129.62, 129.05, 127.21, 127.17, 119.03, 115.32, 110.03, 55.63, 27.66, 25.71. HRMS ($EI^+$) m/z $[M+H]^+$ Calcd for $C_{20}H_{17}ClNO$: 322.0993, Found 322.1998.

**9-bromo-2-(2,6-dichlorophenyl)-5,6-dihydrobenzo[f]isoquinoline (4h):**

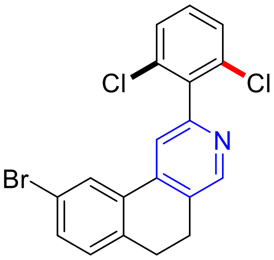


The reaction was carried out according to the general procedure B. (18 mg, 45% yield), brown liquid. $^{1}$H NMR (300 MHz, $CDCl_3$) δ 8.66 (s, 1H), 7.93 (t, $J$ = 3.7 Hz, 1H), 7.62 (s, 1H), 7.50 – 7.44 (m, 3H), 7.35 – 7.29 (m, 1H), 7.20 (s, 1H), 2.97 (d, $J$ = 6.5 Hz, 4H). $^{13}$C NMR (75 MHz, $CDCl_3$) δ 154.64, 149.21, 141.26, 137.12, 134.94, 133.92, 132.54, 131.19, 131.17, 130.36, 130.29, 130.06, 129.84, 128.33, 127.56, 121.07, 119.01, 27.95, 25.17. HRMS ($EI^+$) m/z $[M+H]^+$ Calcd for $C_{19}H_{13}BrCl_2N$: 403.9603 and 405.9582, Found 403.9598 and 405.9584.

**8-methoxy-2-(2-(phenylselanyl)phenyl)-5,6-dihydrobenzo[f]isoquinoline(5):**

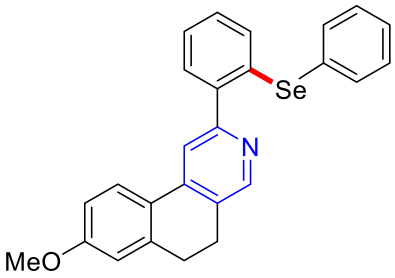


The reaction was carried out according to the general procedure C. brown semi-solid, 27 mg (61% yield). $^{1}$H NMR (700 MHz, DMSO) δ 8.54 (s, 1H), 8.07 (s, 1H), 8.01 (d, J = 8.6 Hz, 1H), 7.85 (d, J = 7.6 Hz, 1H), 7.55 (d, J = 6.9 Hz, 2H), 7.41 – 7.31 (m, 4H), 7.21 (t, J = 7.7 Hz, 1H), 7.07 (d, J = 8.0 Hz, 1H), 6.95 – 6.95 (m, 2H), 3.82 (s, 3H), 2.89 (s, 4H). $^{13}$C NMR (176 MHz, DMSO) δ 160.65, 156.81, 147.12, 142.15, 140.30, 139.76, 135.71, 133.97, 131.60, 130.87, 130.07, 130.02, 129.98, 129.86, 129.62, 129.06, 128.66, 126.70, 126.34, 124.35, 115.81, 113.87, 113.25, 55.51, 28.27, 24.69. HRMS (EI$^{+}$) m/z $[M+H]^{+}$ Calcd for $C_{26}H_{22}NOSe$: 444.0861, Found 444.0856.

# 3. $^{1}$H and $^{13}$C NMR Spectra

## (2-(8-methoxy-5,6-dihydrobenzo[f]isoquinolin-2-yl)phenyl)methanol (3a):

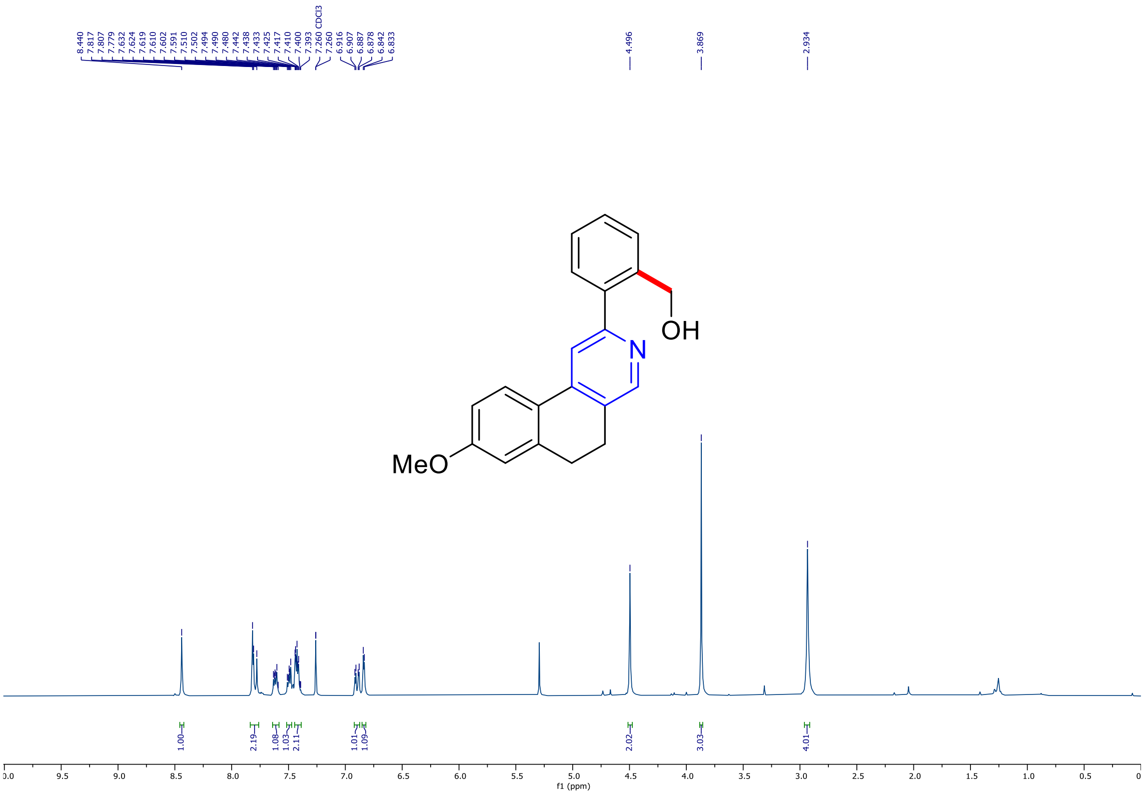


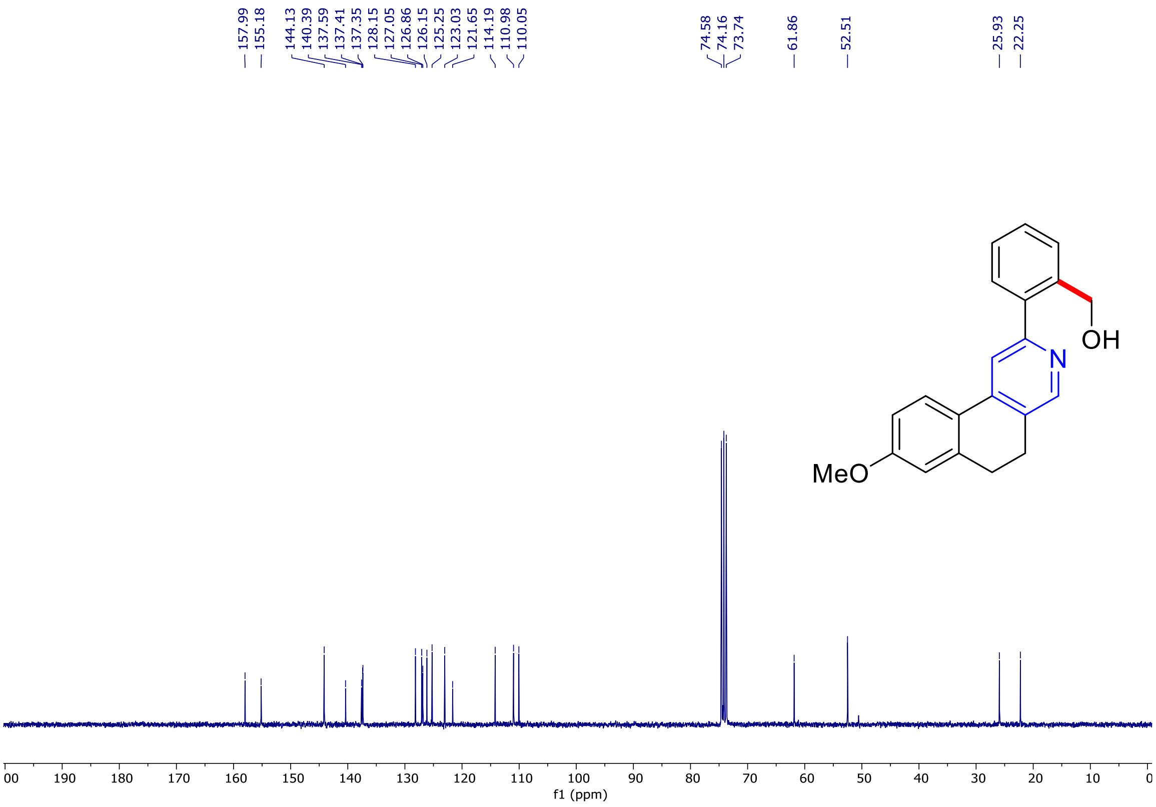

# (2-(9-methoxy-5,6-dihydrobenzo[f]isoquinolin-2-yl)phenyl)methanol (3b):

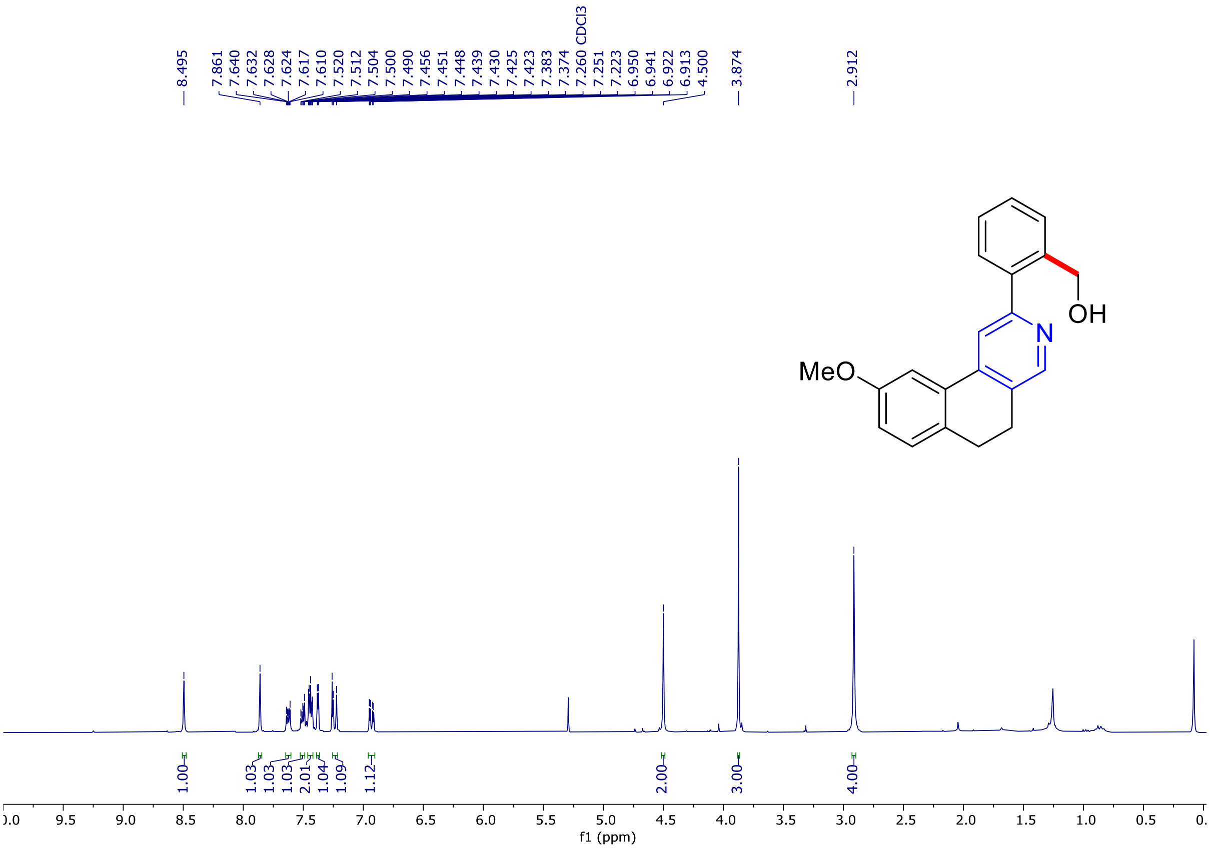

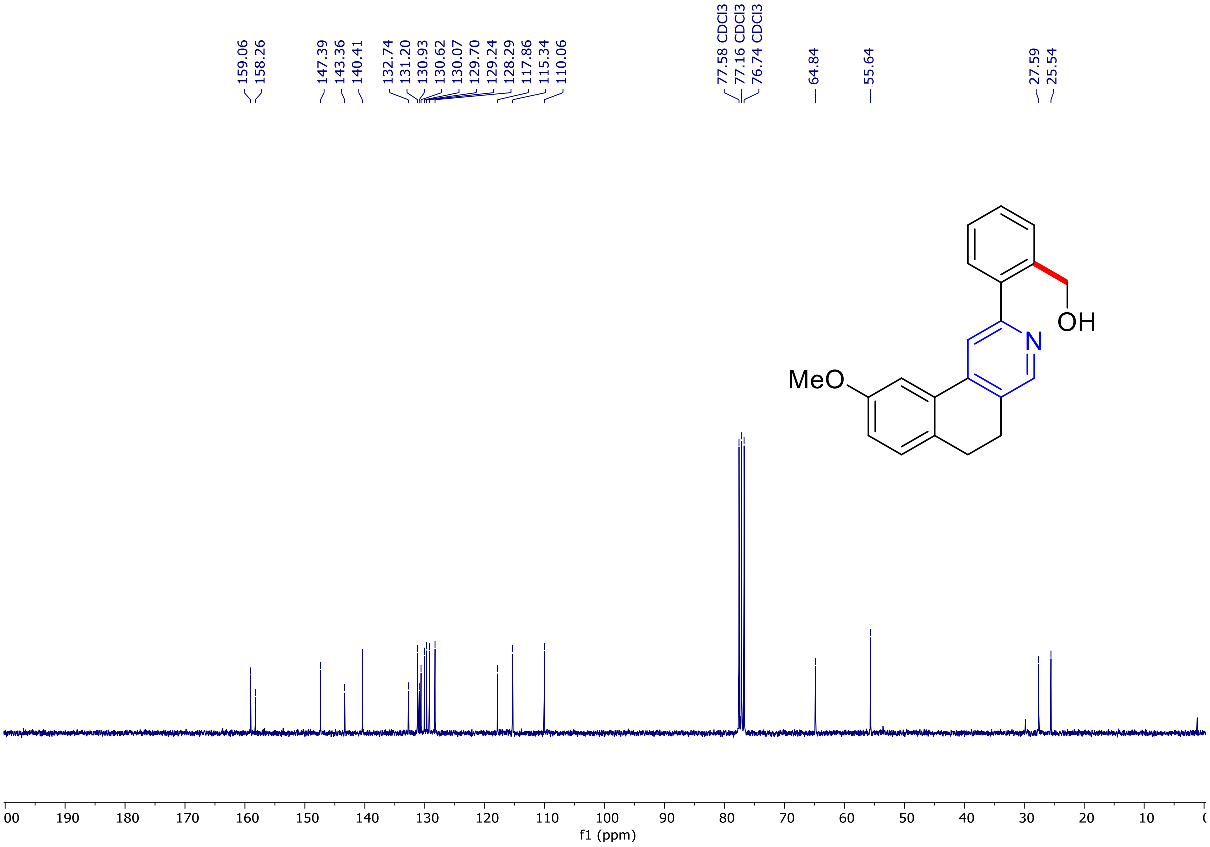

**(2-(5,6-dihydrobenzo[f]isoquinolin-2-yl)phenyl)methanol (3c):**

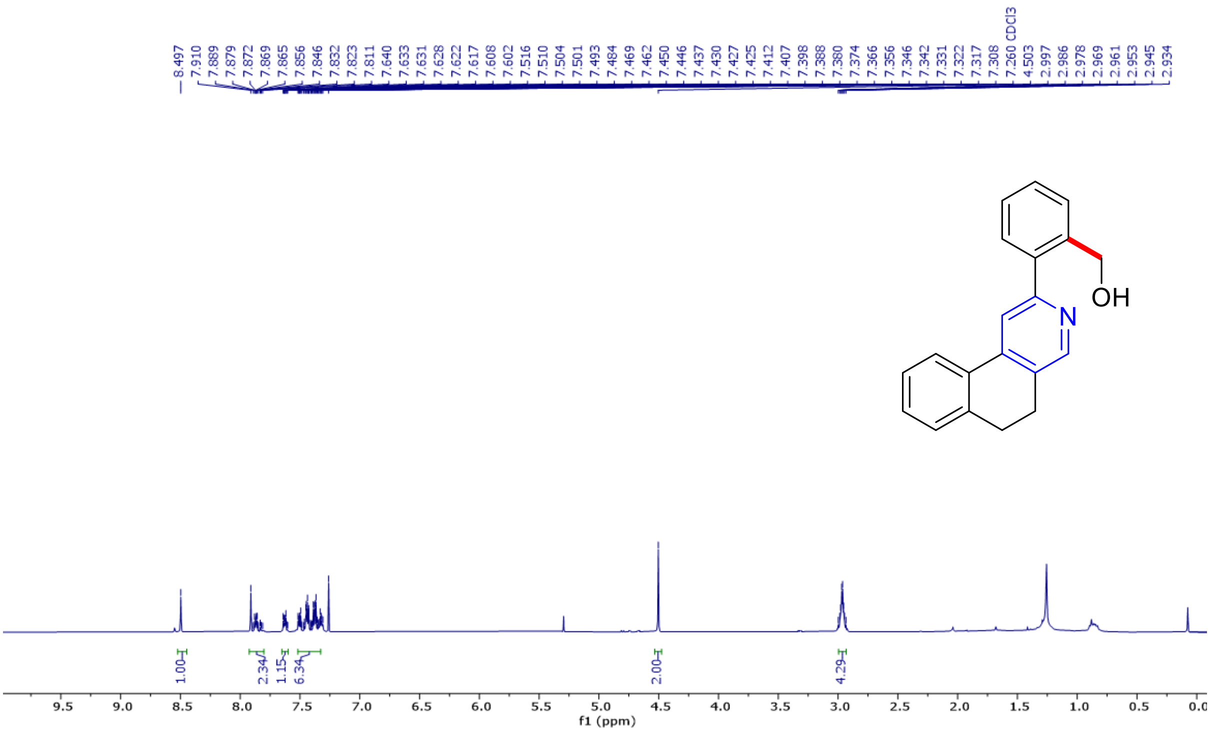


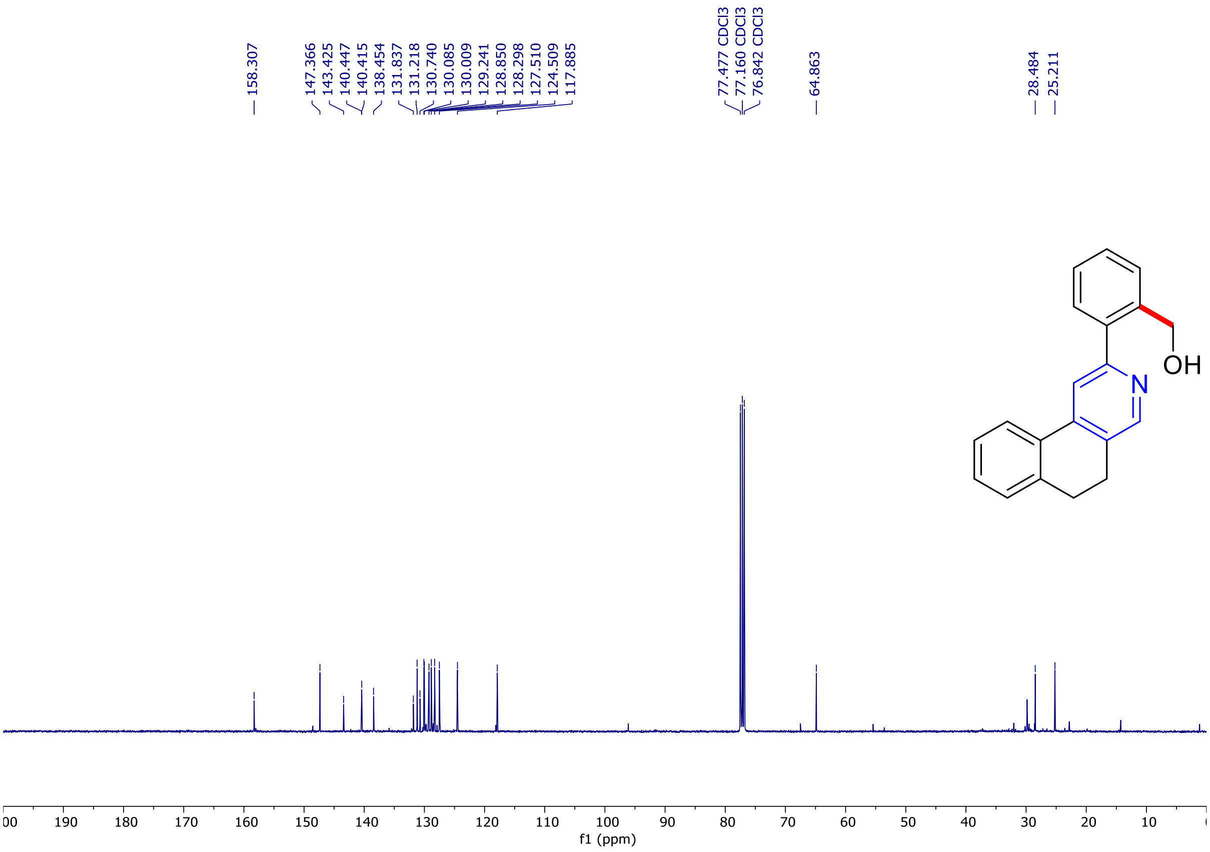

**(2-(6-methyl-5,6-dihydrobenzo[f]isoquinolin-2-yl)phenyl)methanol (3d):**

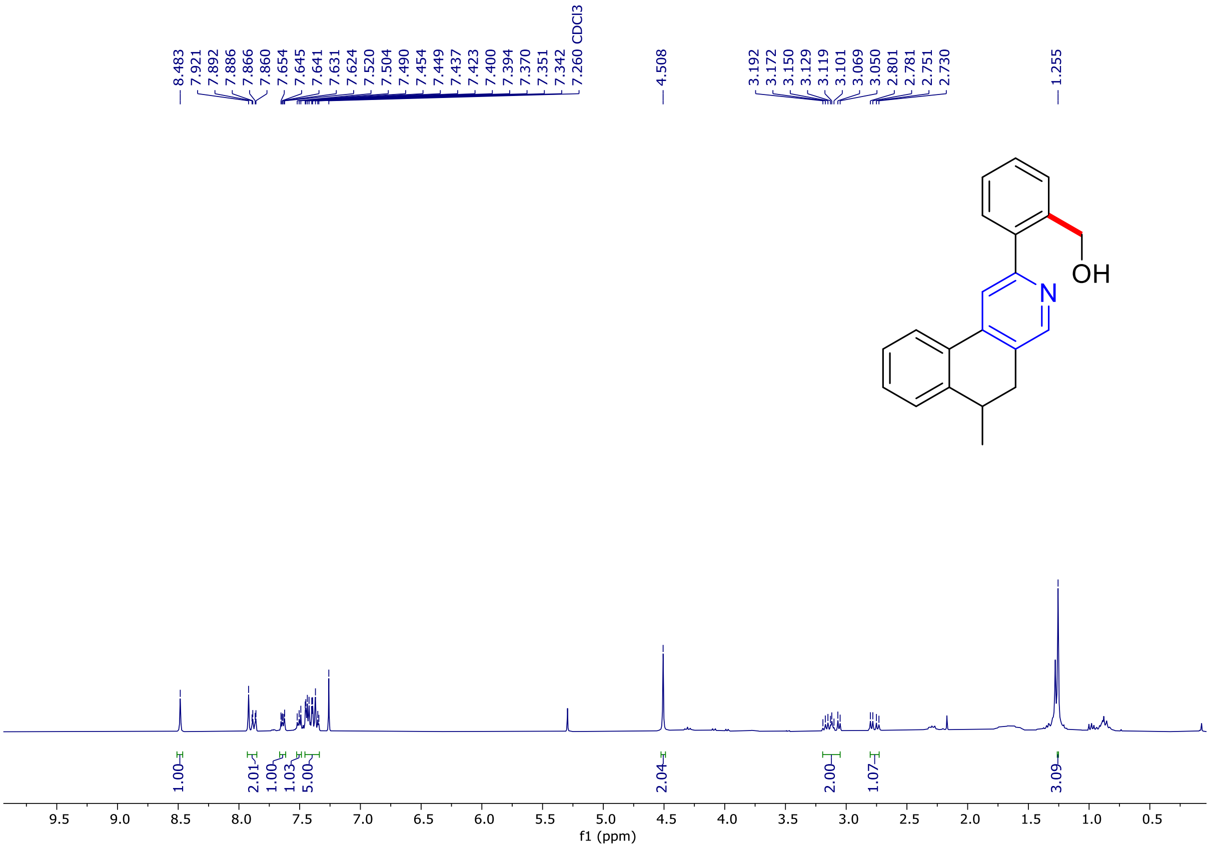


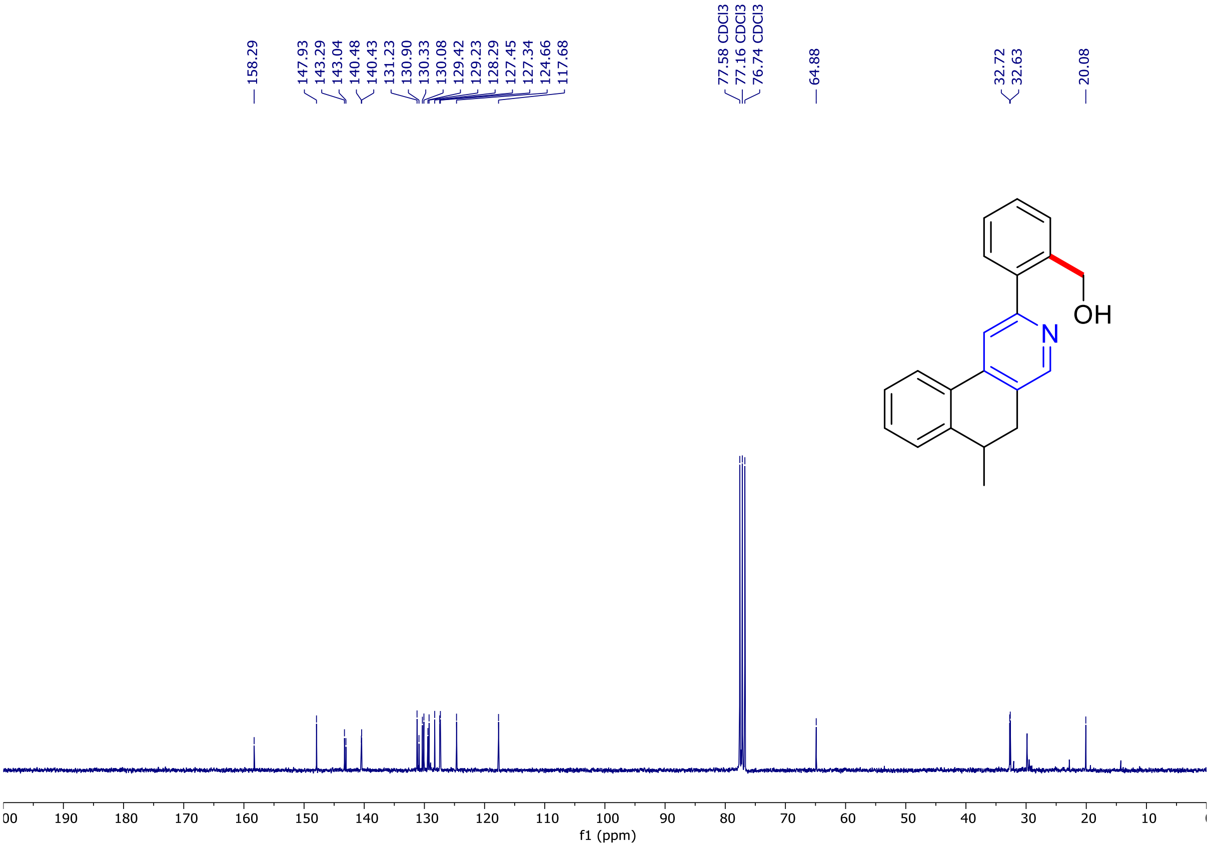

**(5-fluoro-2-(9-methoxy-5,6-dihydrobenzo[f]isoquinolin-2-yl)phenyl)methanol (3e):**

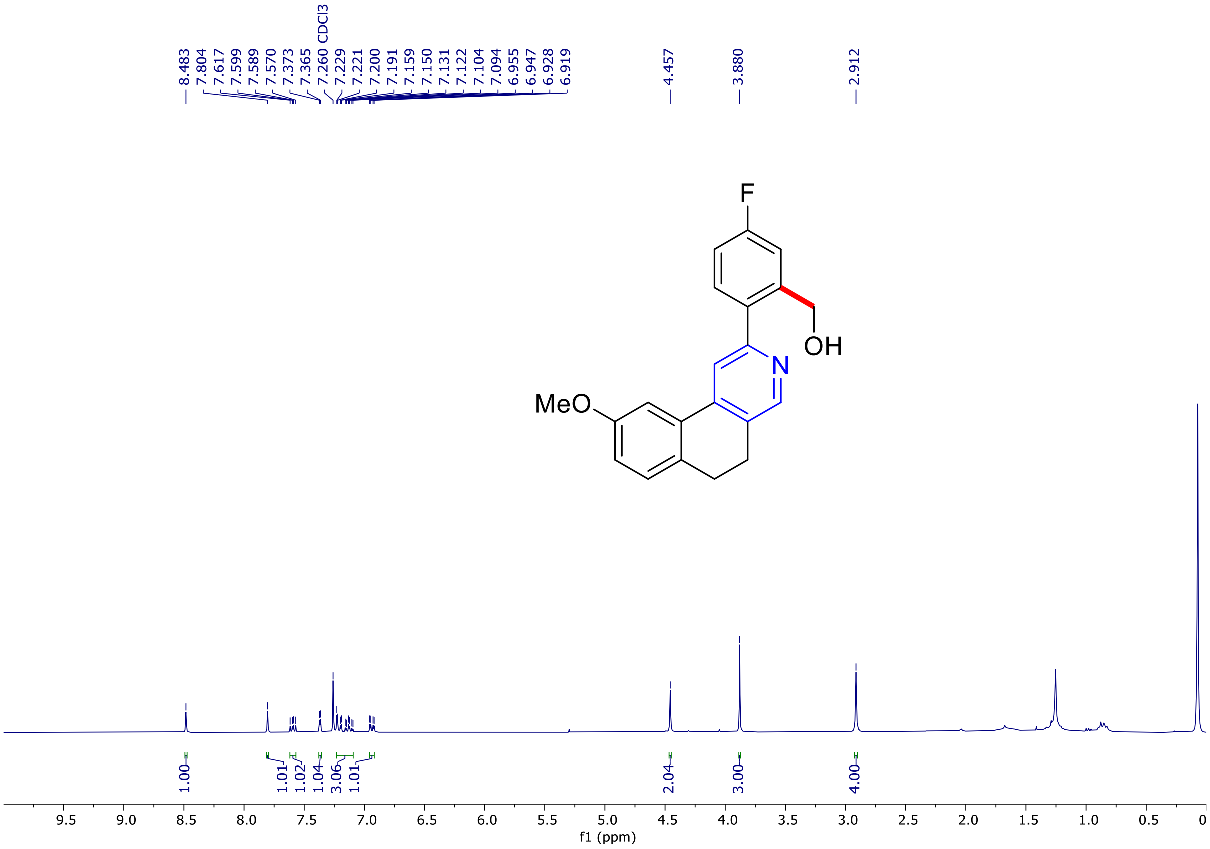


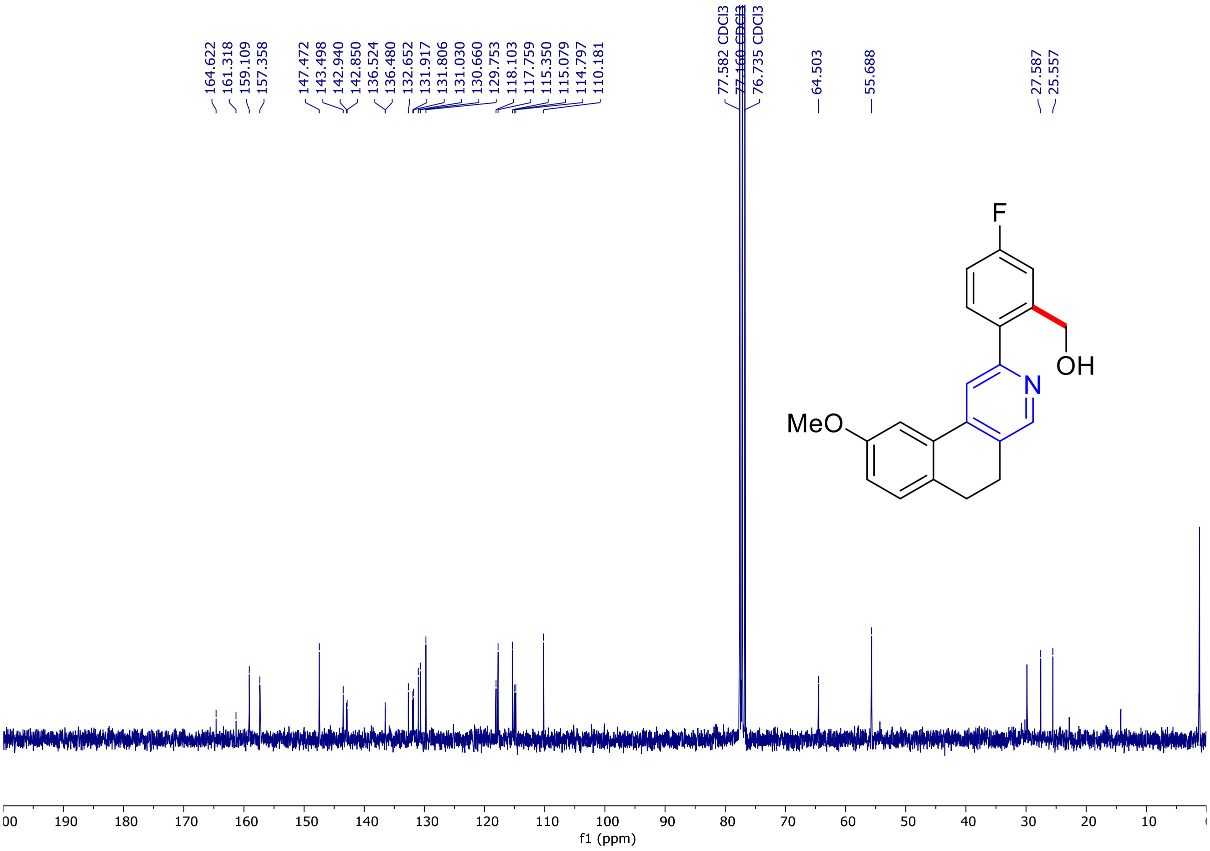

## (2-(7-methoxy-5,6-dihydrobenzo[f]isoquinolin-2-yl)-4-methylphenyl)methanol (3f):

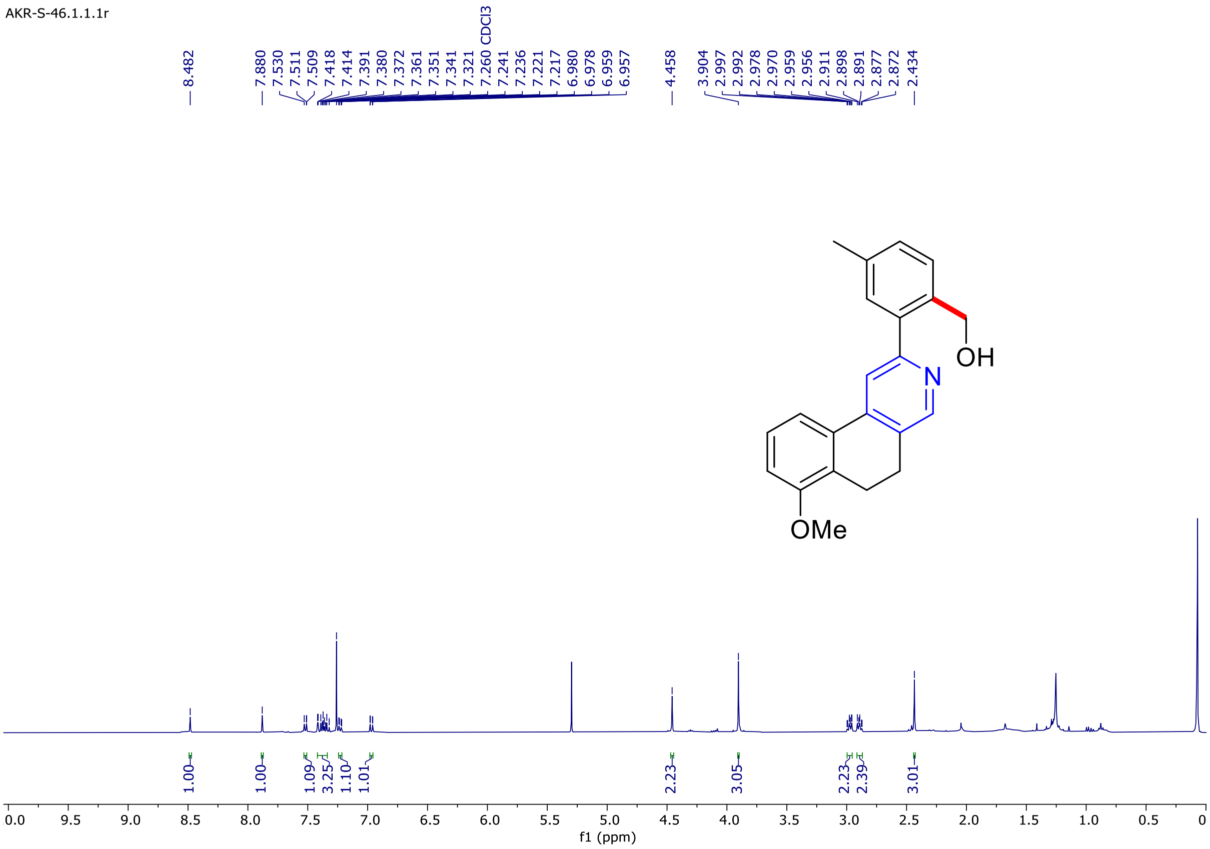


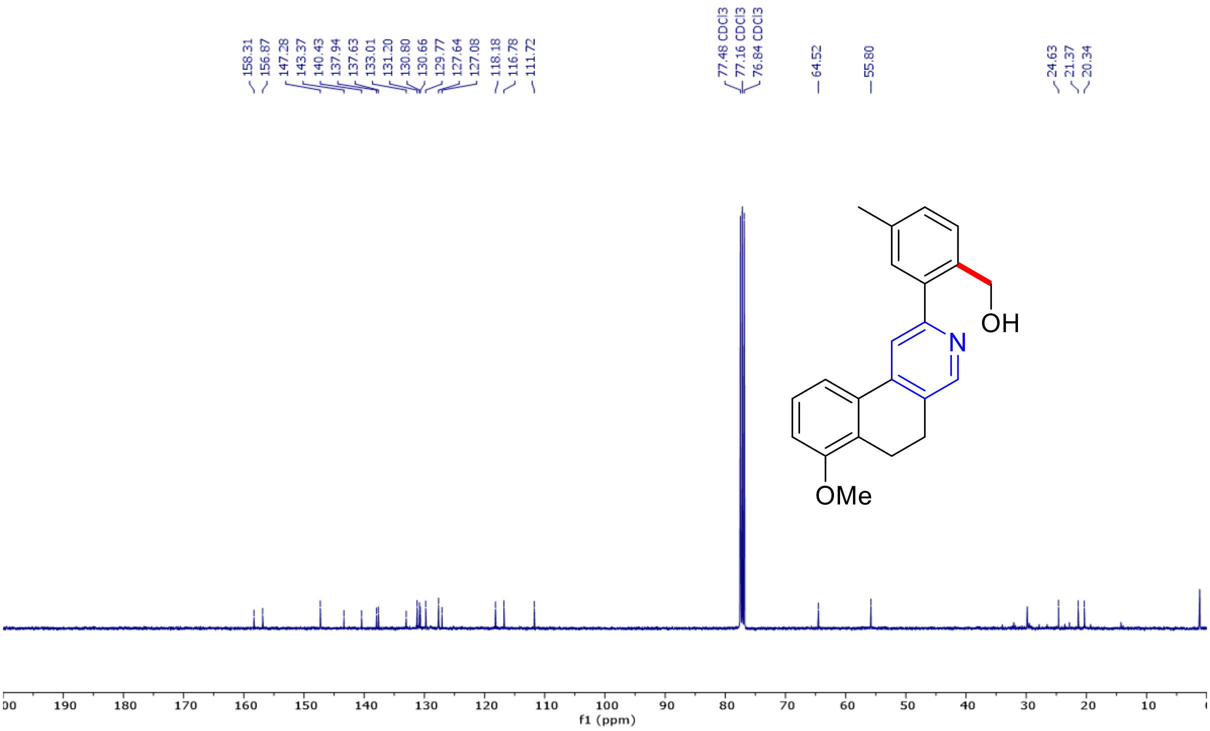

**(2-(8-methoxy-5,6-dihydrobenzo[f]isoquinolin-2-yl)-5-methylphenyl)methanol (3g):**

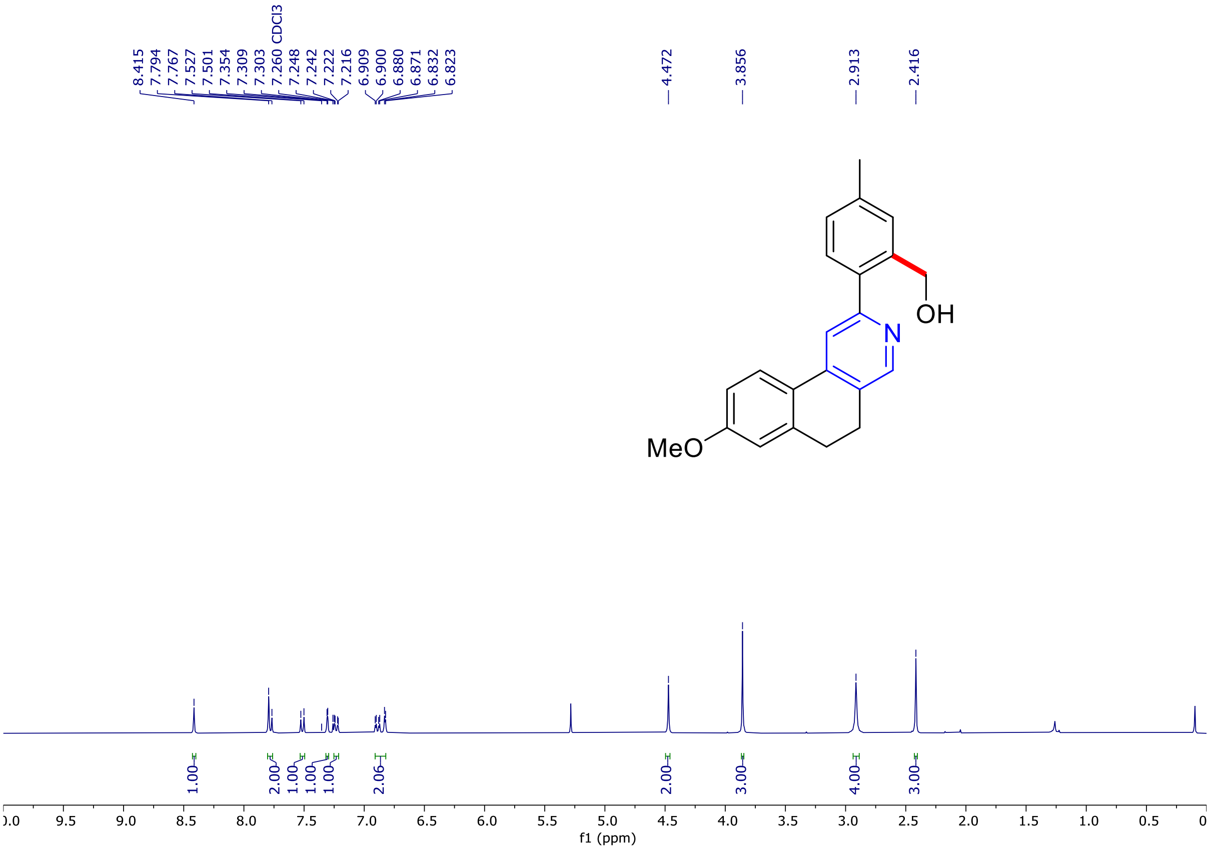


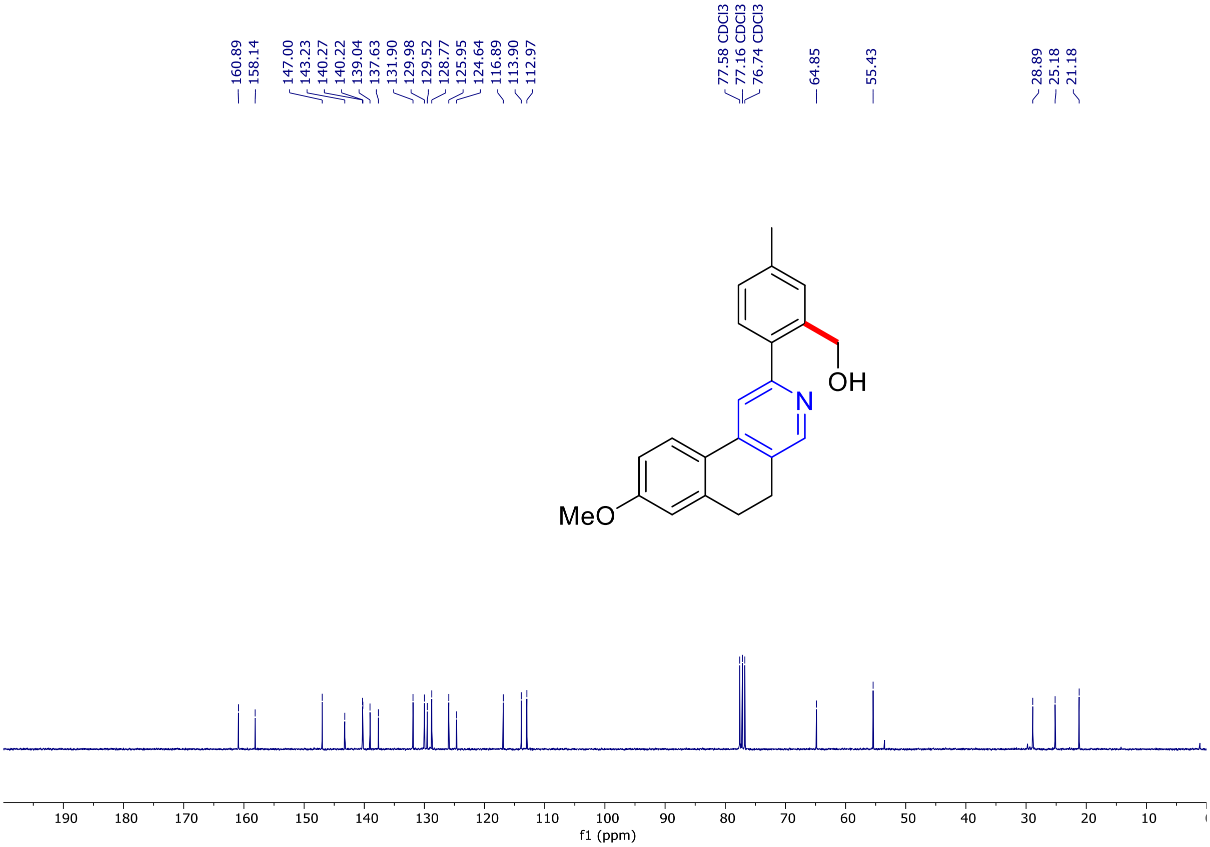

**(5-methyl-2-(6-methyl-5,6-dihydrobenzo[f]isoquinolin-2-yl)phenyl)methanol (3h)**

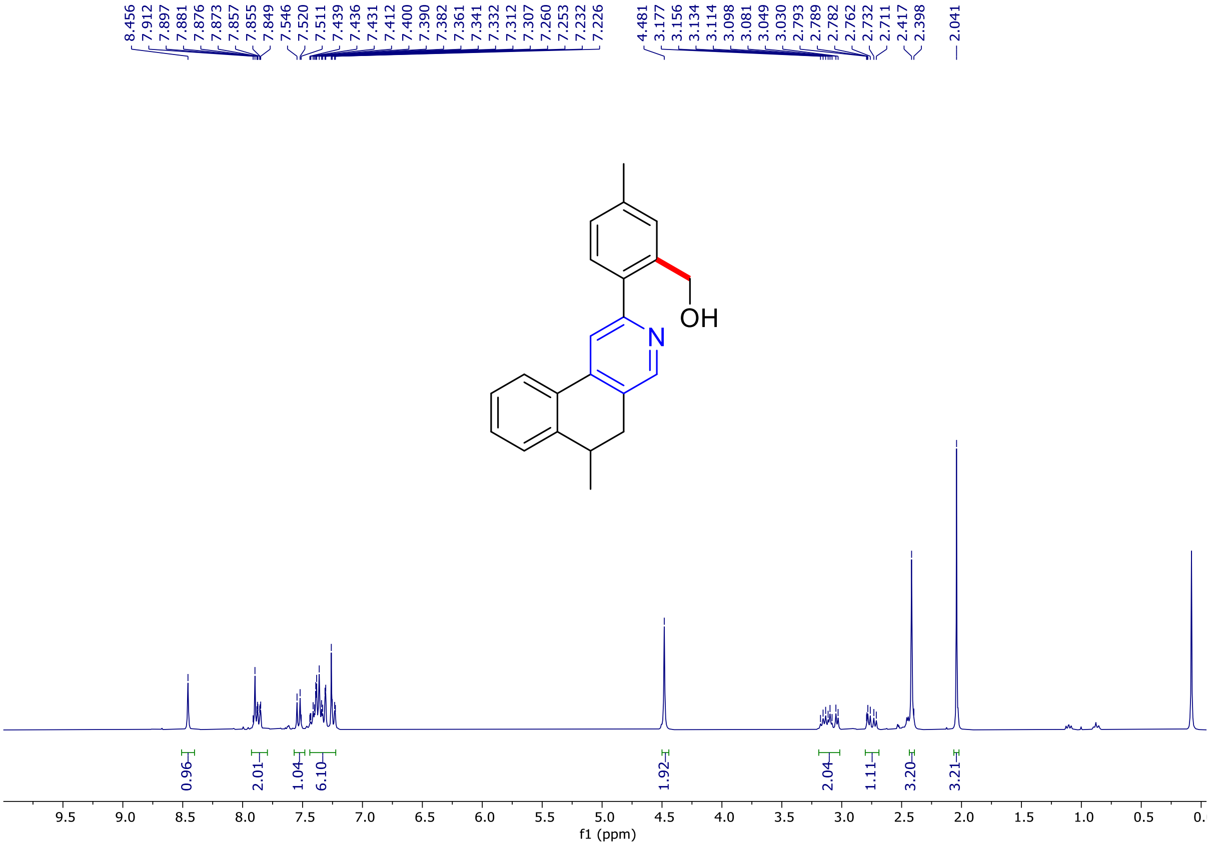


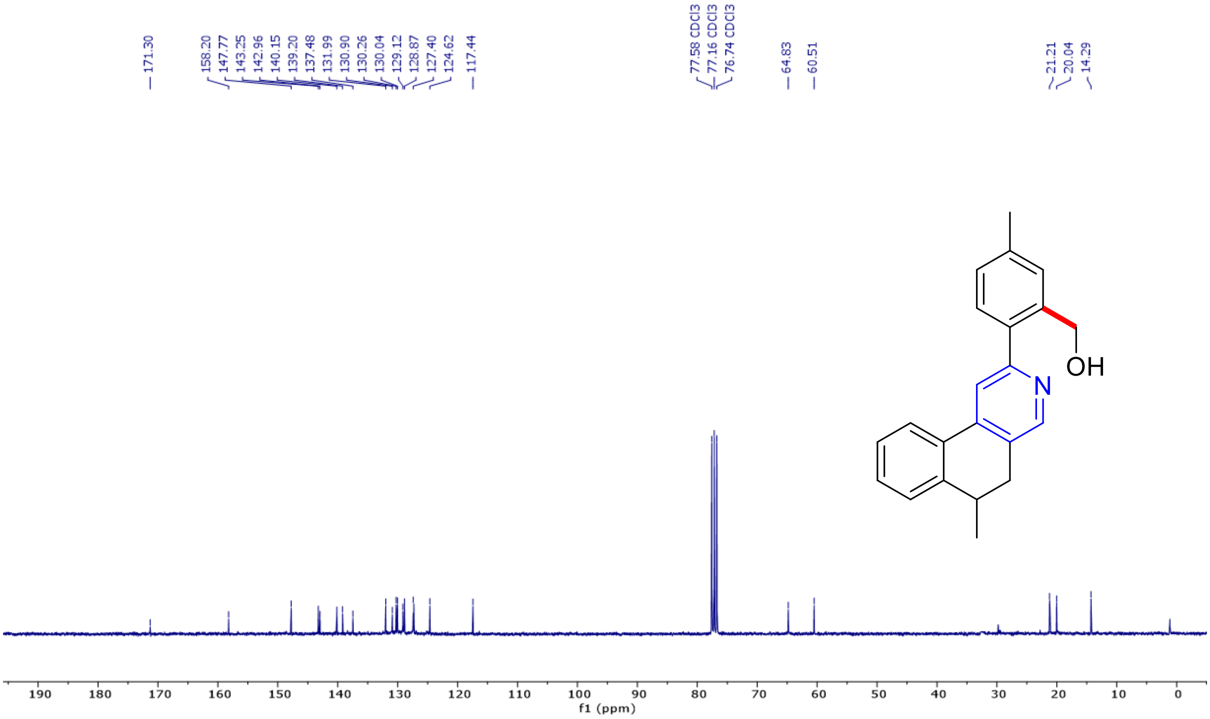

**(2-(9-bromo-5,6-dihydrobenzo[f]isoquinolin-2-yl)-5-methylphenyl)methanol (3i)**

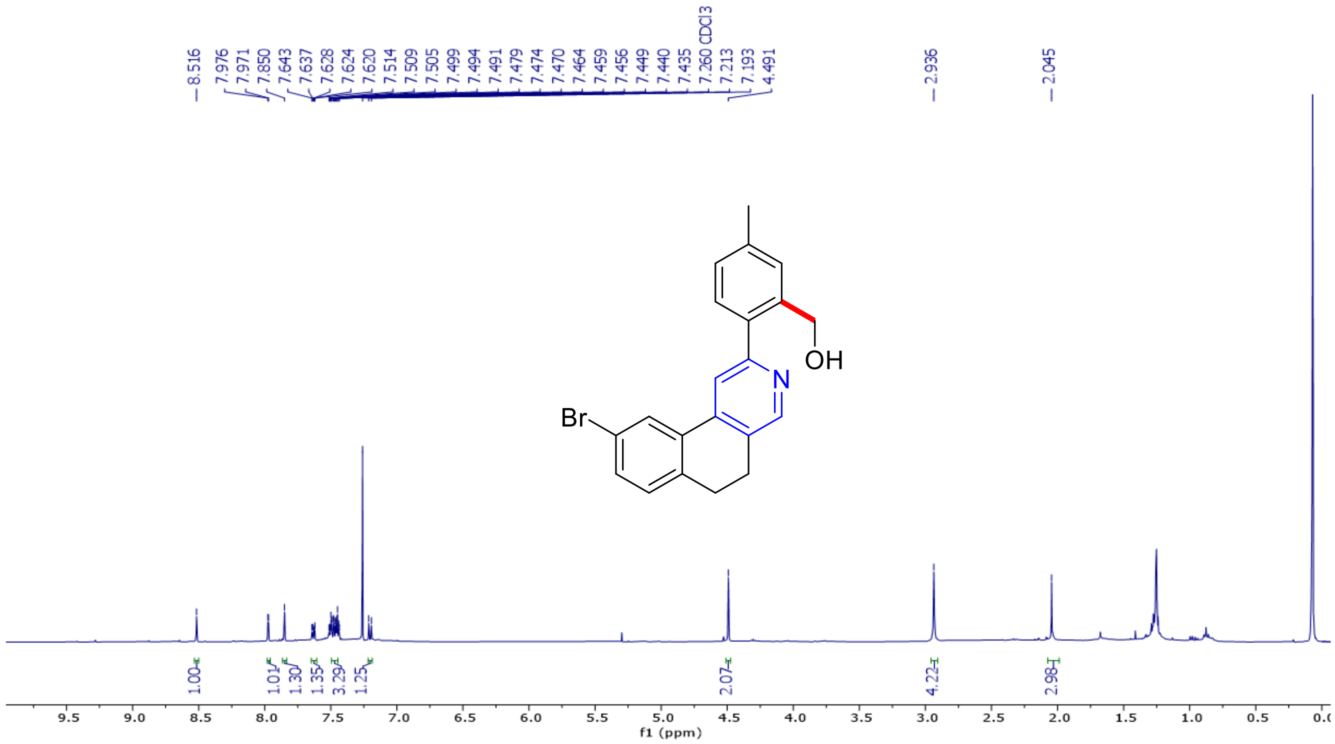

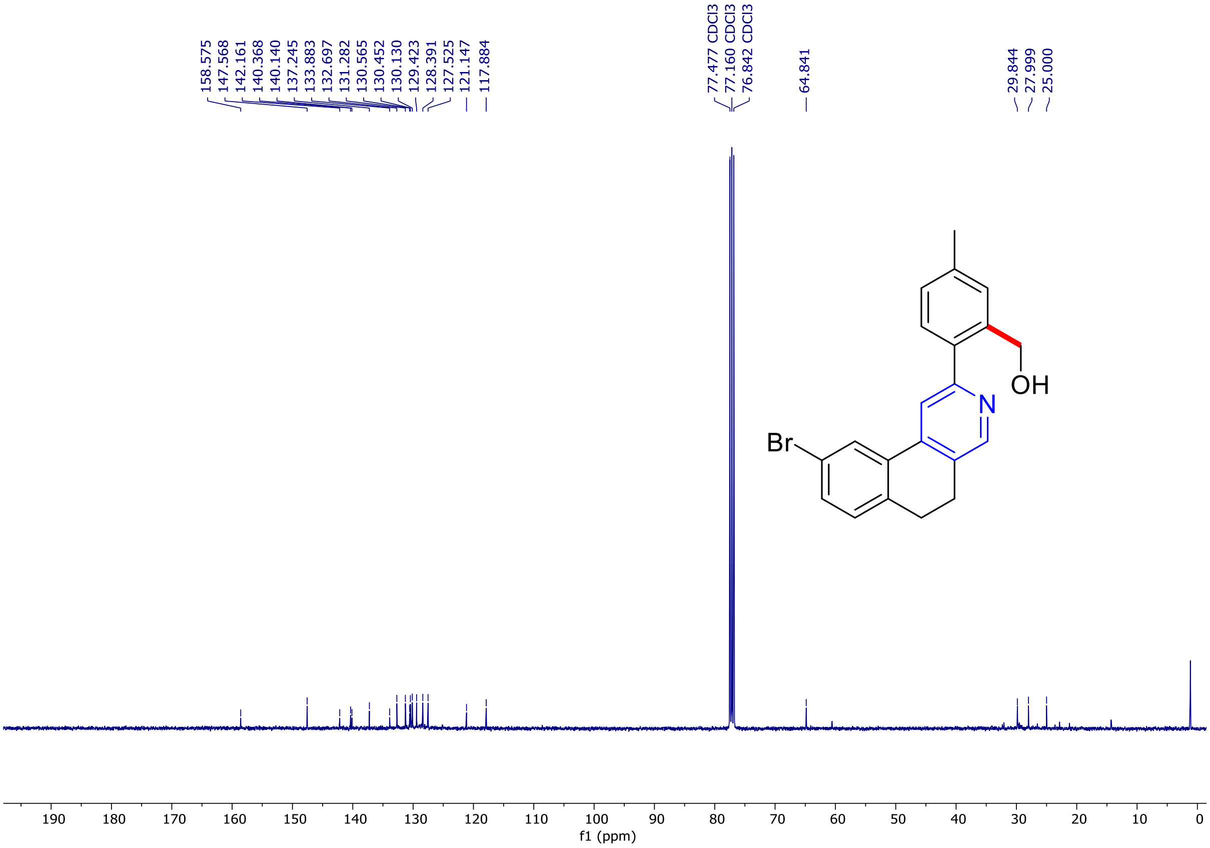

# (2-(9-bromo-5,6-dihydrobenzo[f]isoquinolin-2-yl)-5-methylphenyl)methanol(3j):

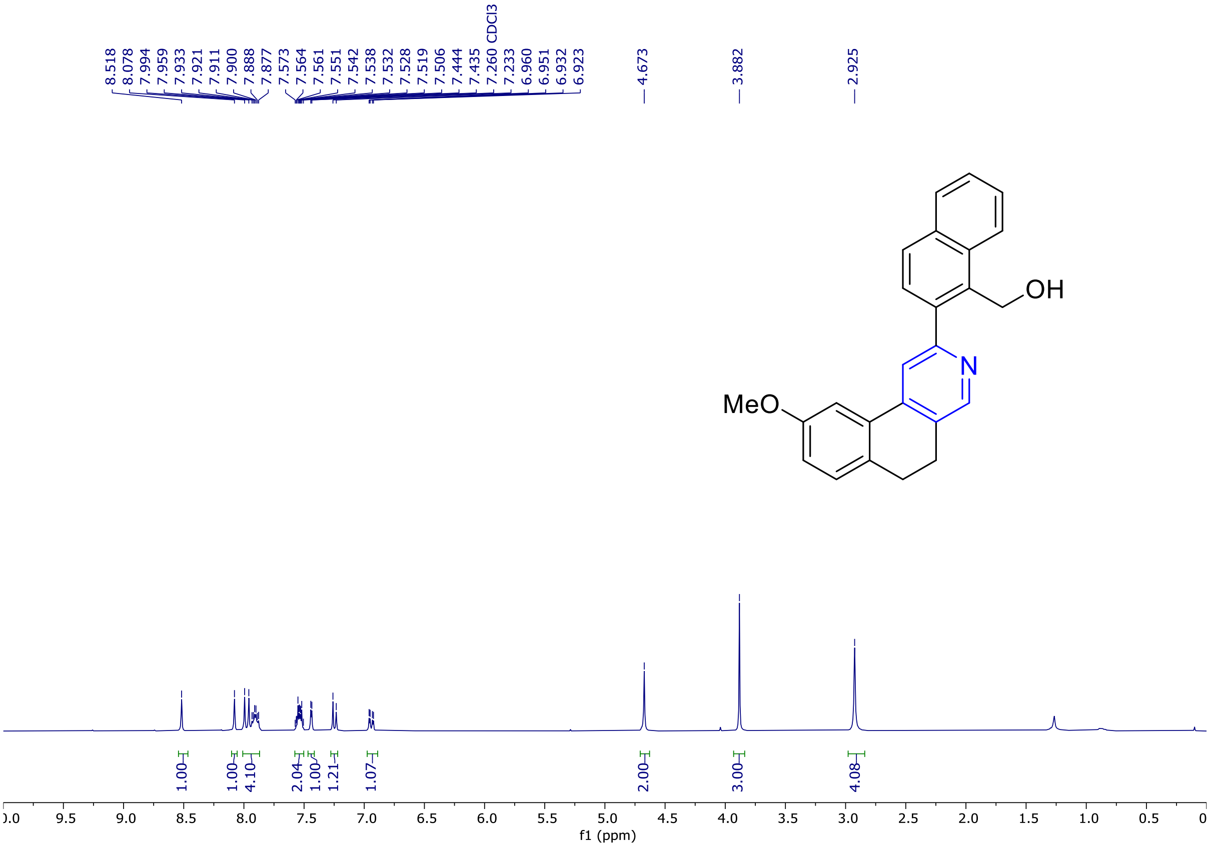


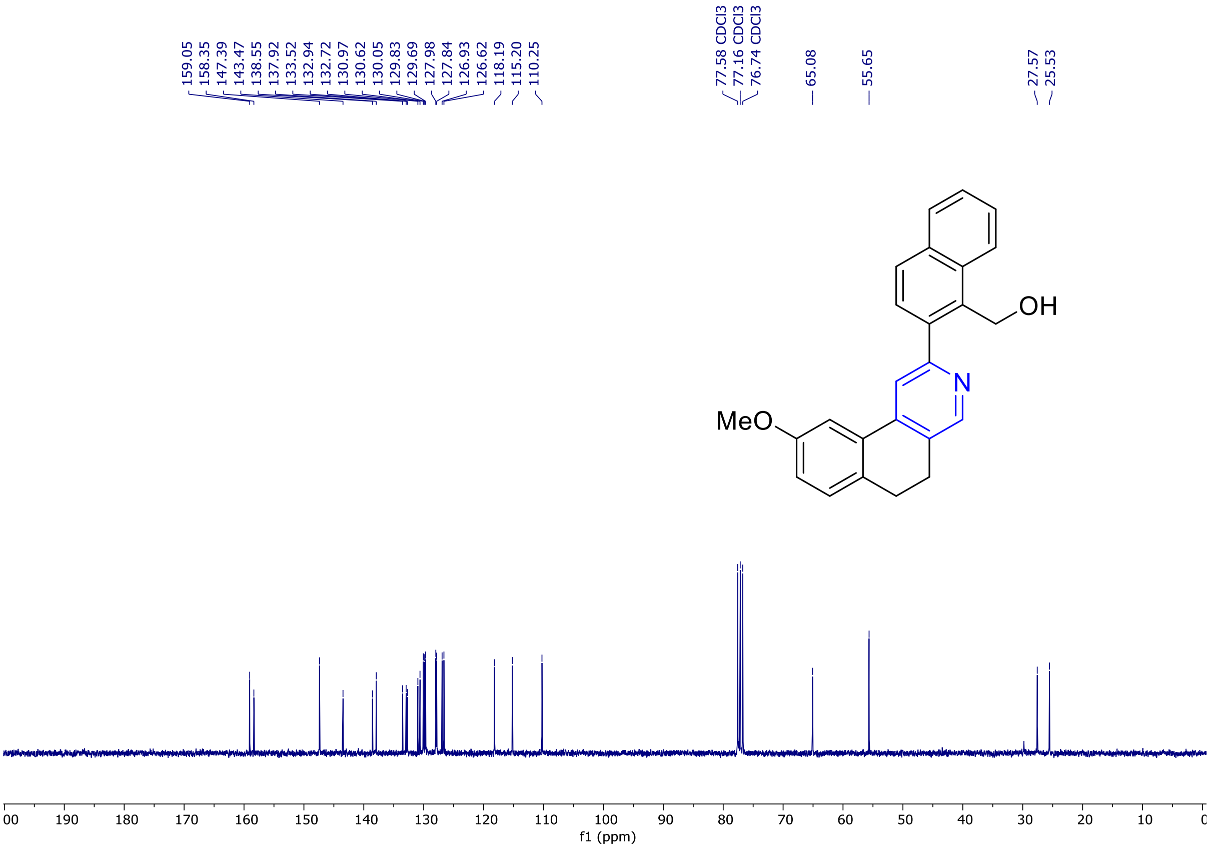

**(2-(9-methoxy-5,6-dihydrobenzo[f]isoquinolin-2-yl)-5-methylphenyl)methanol (3k):**

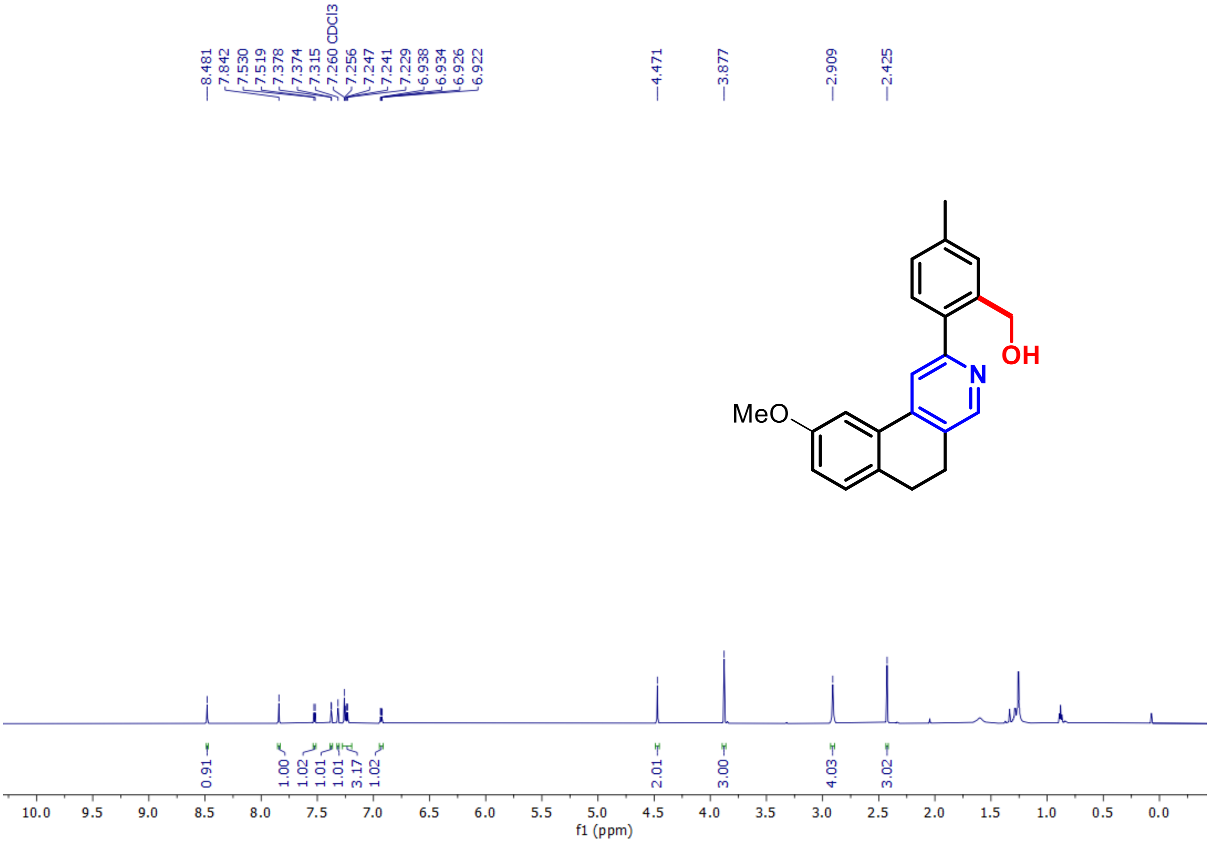


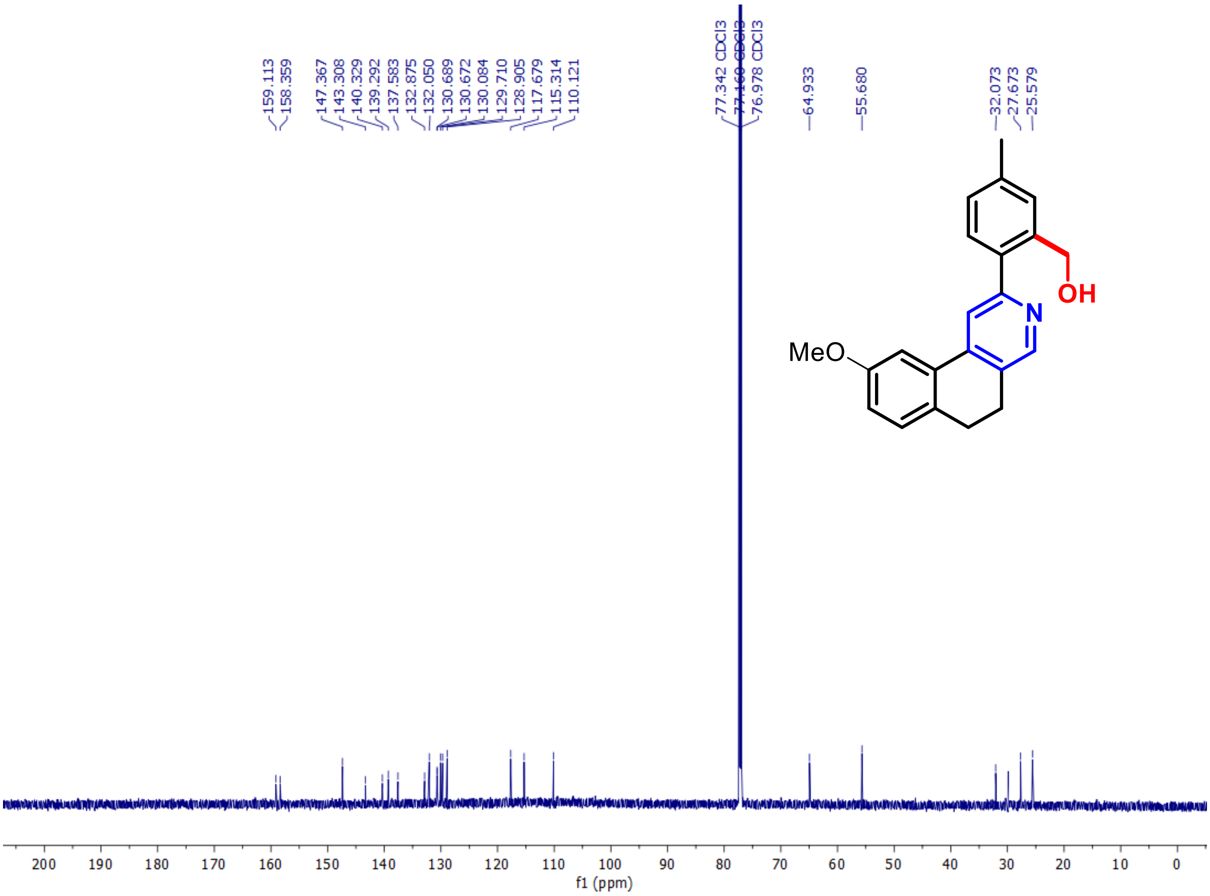

**2-(2-bromophenyl)-8-methoxy-5,6-dihydrobenzo[f]isoquinoline (4a)**

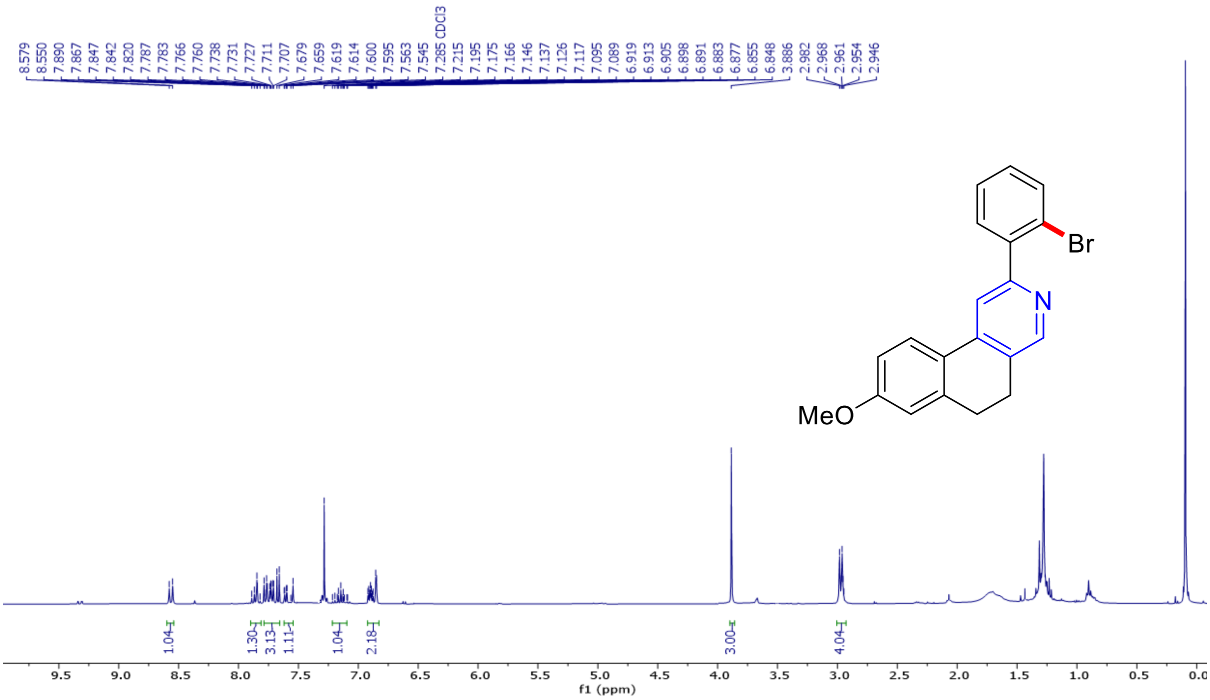

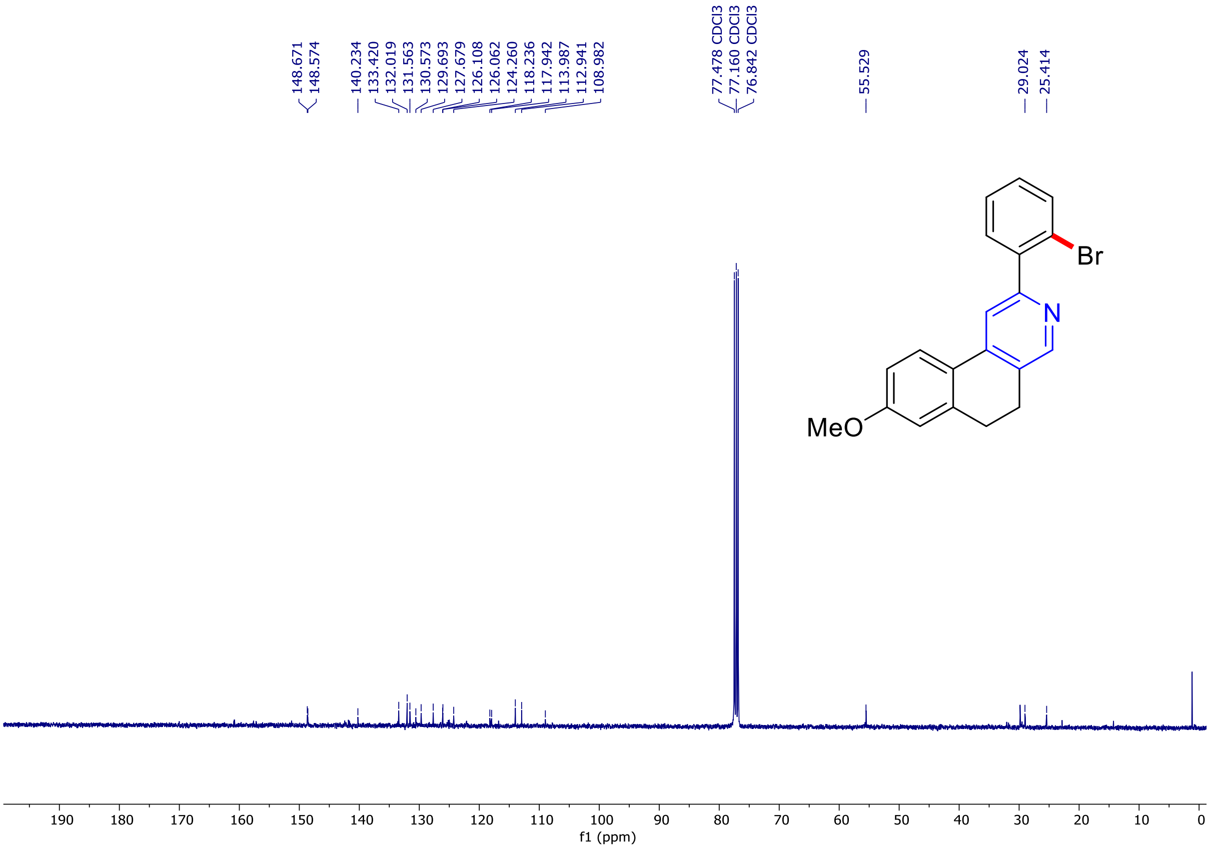

**2-(2-bromophenyl)-5,6-dihydrobenzo[f]isoquinoline (4b):**

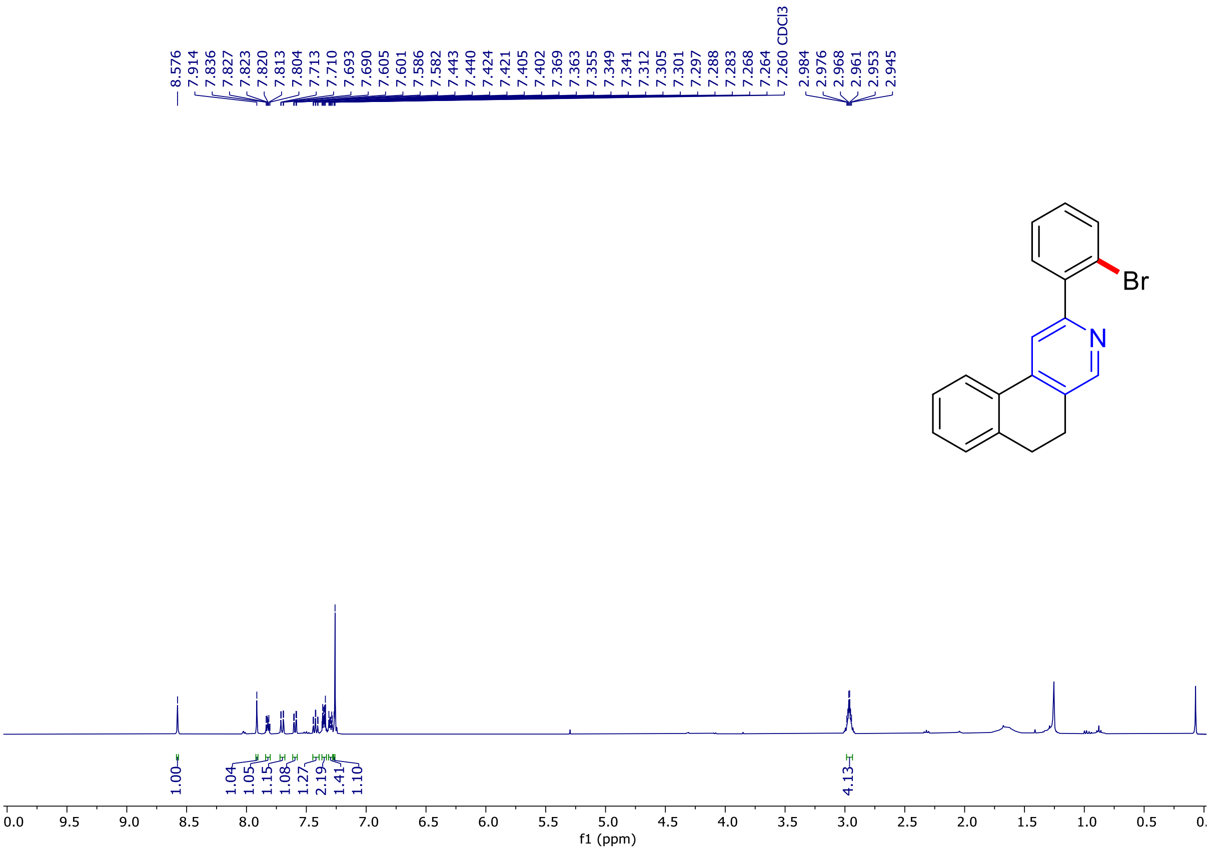


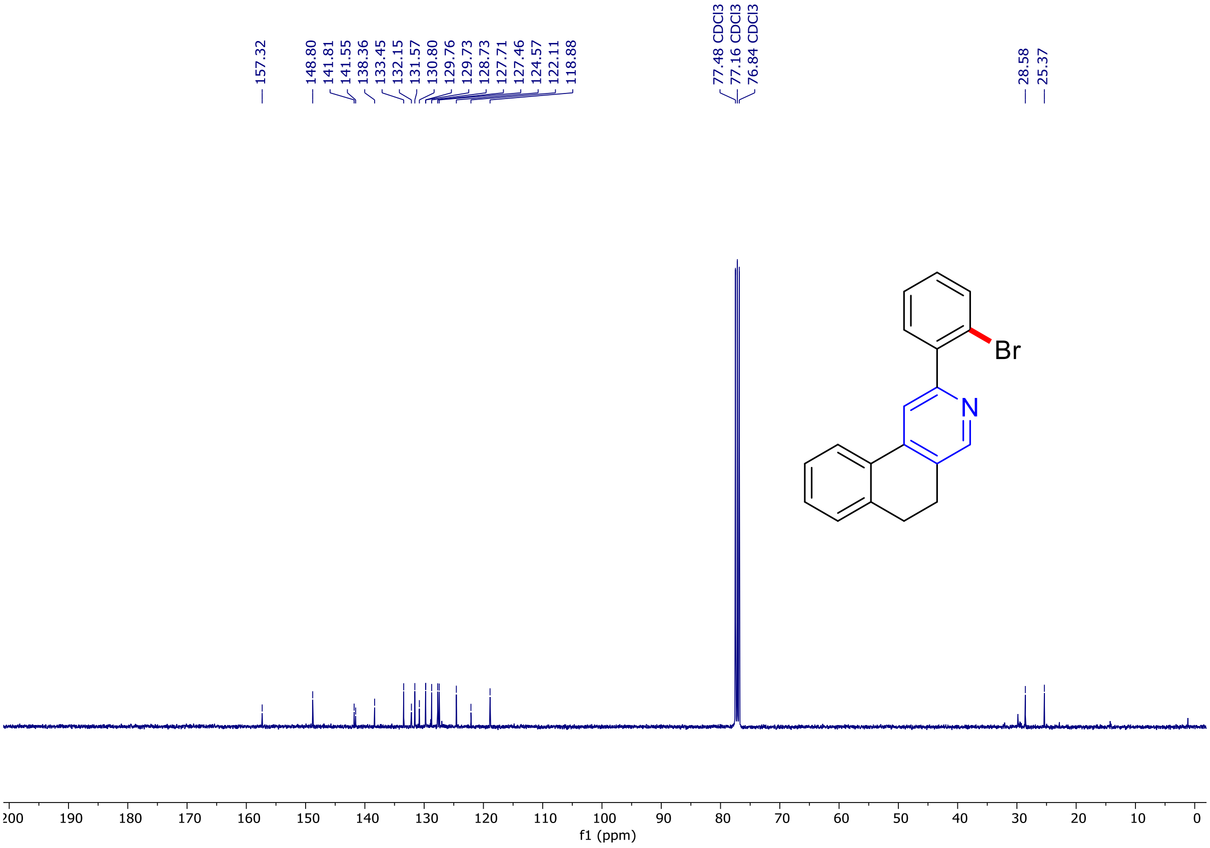

**2-(1-bromonaphthalen-2-yl)-8-methoxy-5,6-dihydrobenzo[f]isoquinoline (4c):**

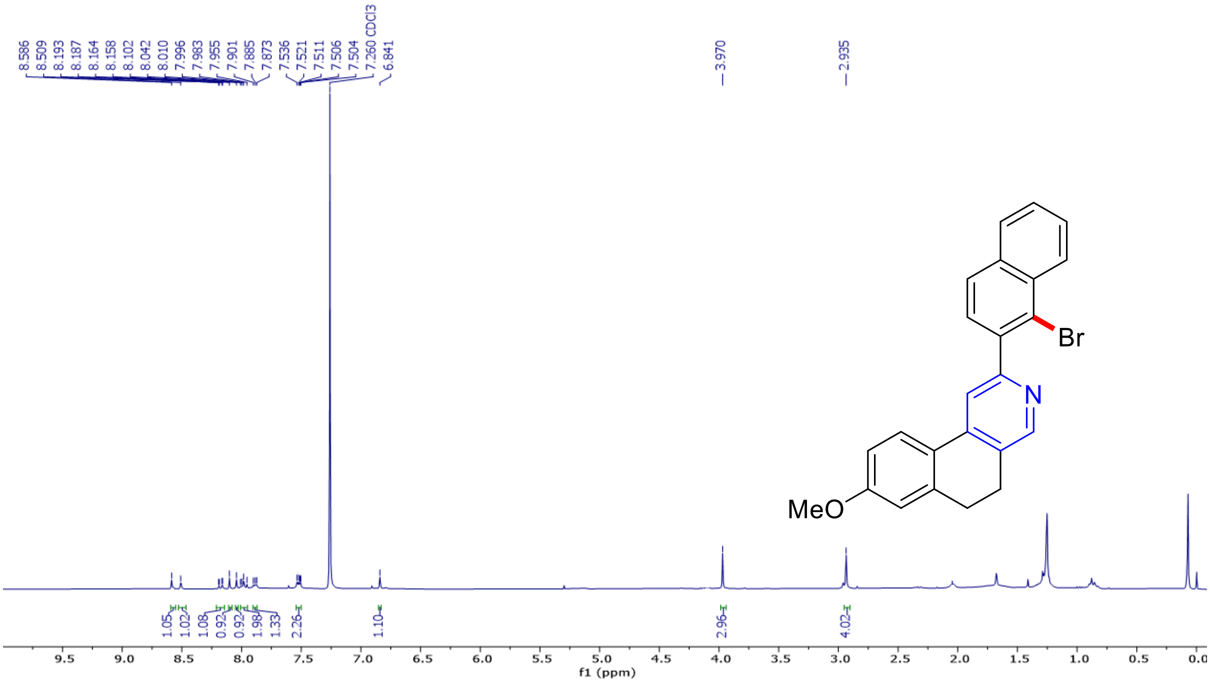


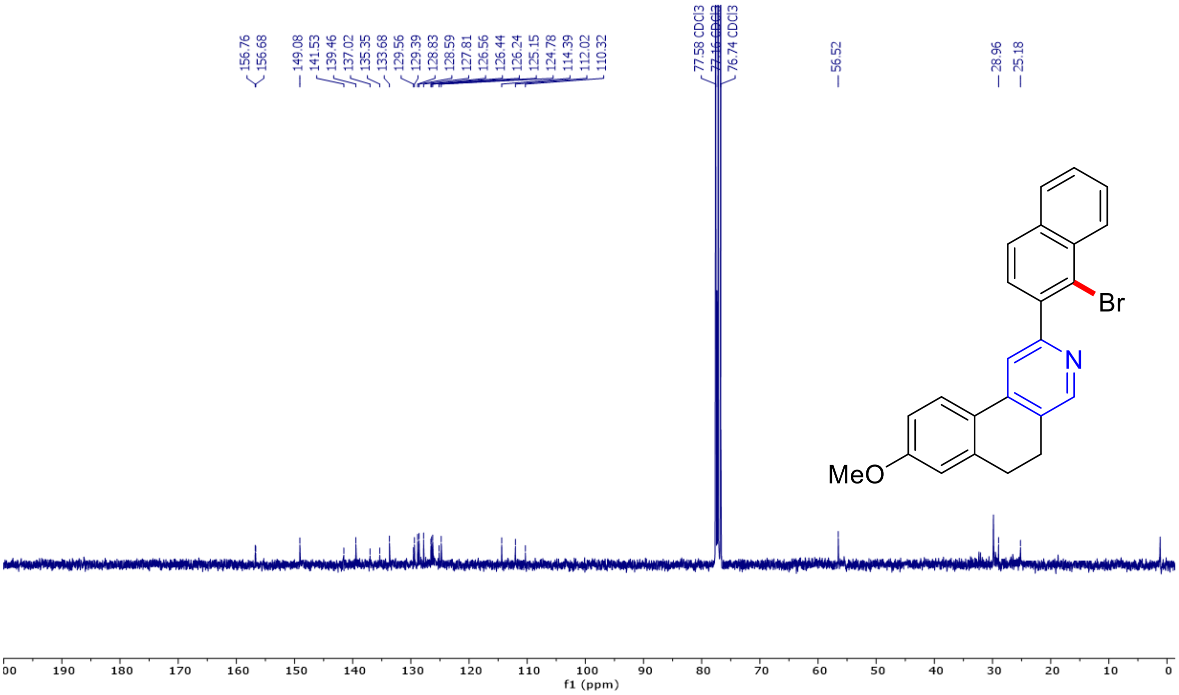

**2-(2-bromo-4-methylphenyl)-8-methoxy-5,6-dihydrobenzo[f]isoquinoline (4d):**

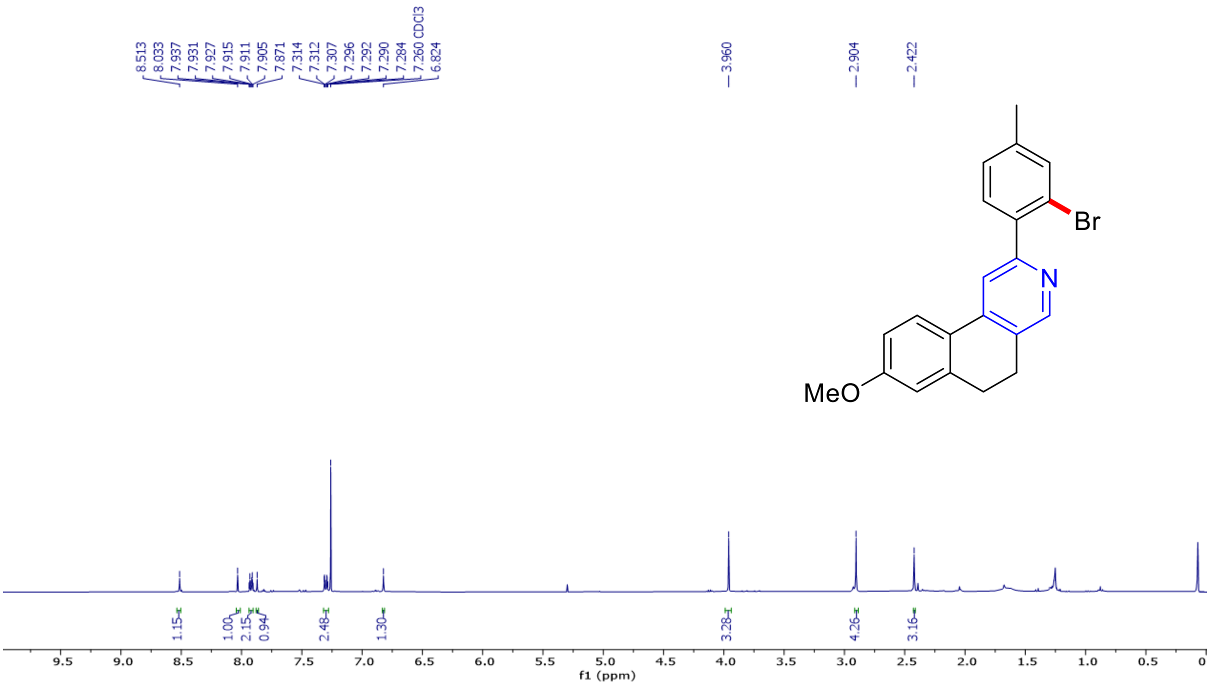


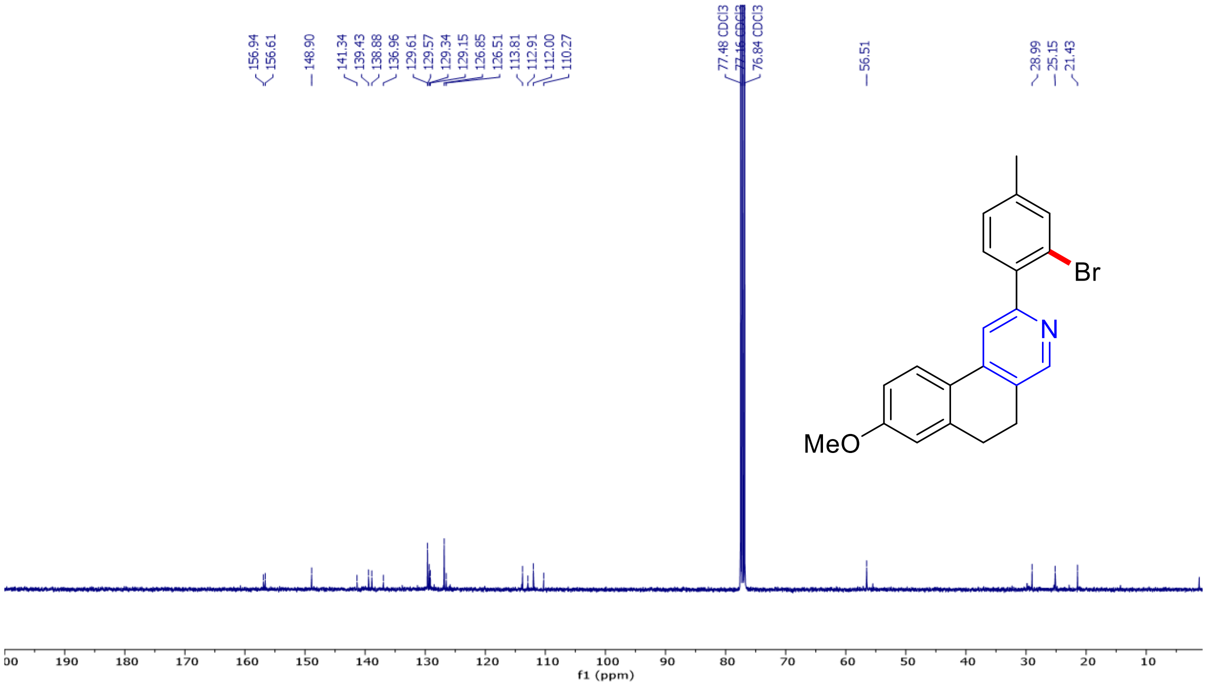

**2-(2-bromo-4-methylphenyl)-9-methoxy-5,6-dihydrobenzo[f]isoquinoline (4e):**

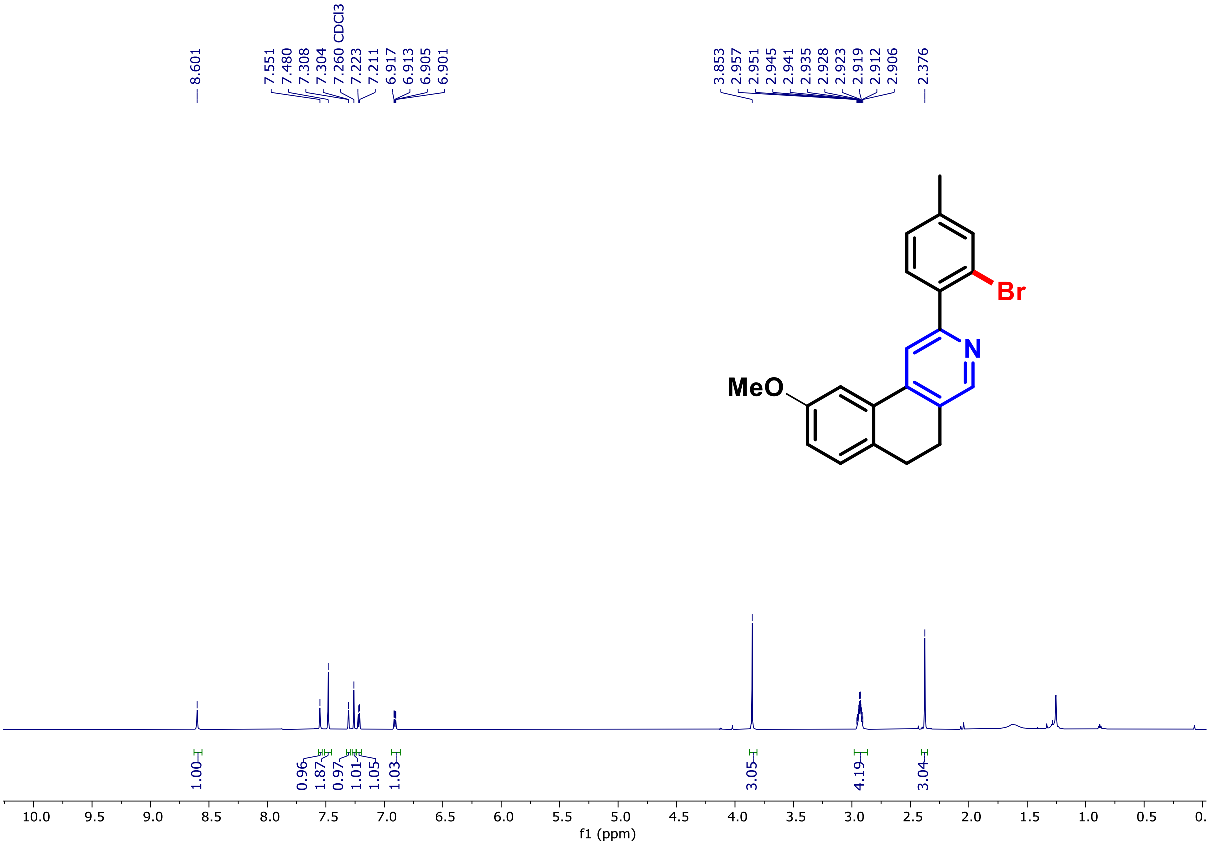


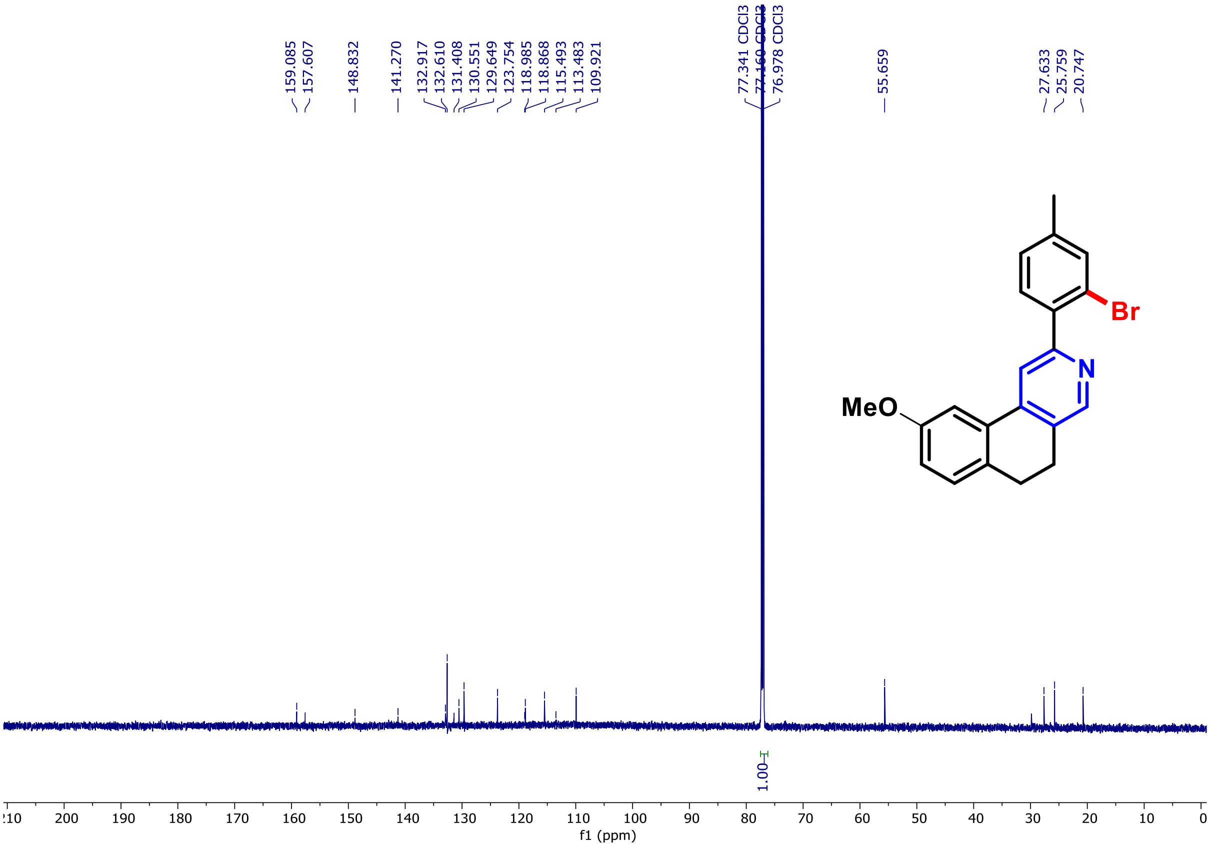

**2-(2-chlorophenyl)-6-methyl-5,6-dihydrobenzo[f]isoquinoline (4f)**

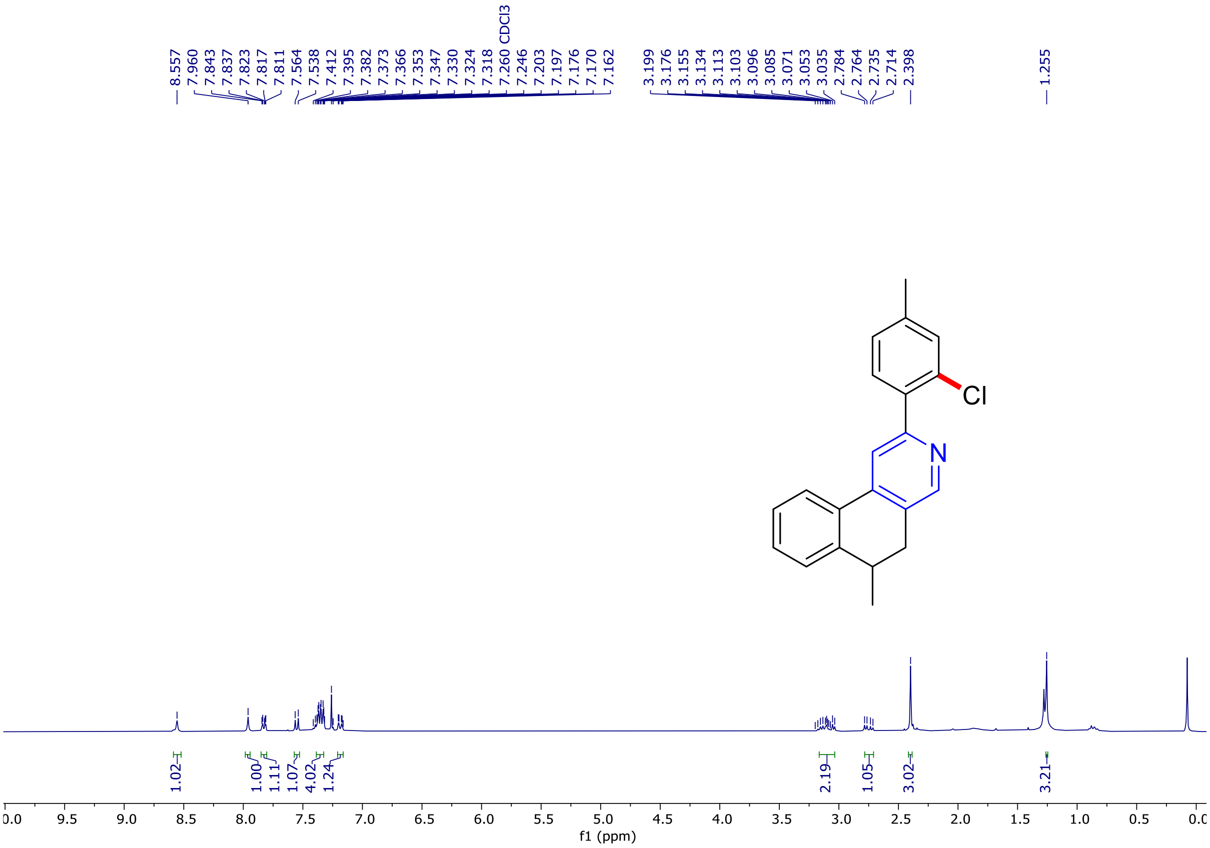


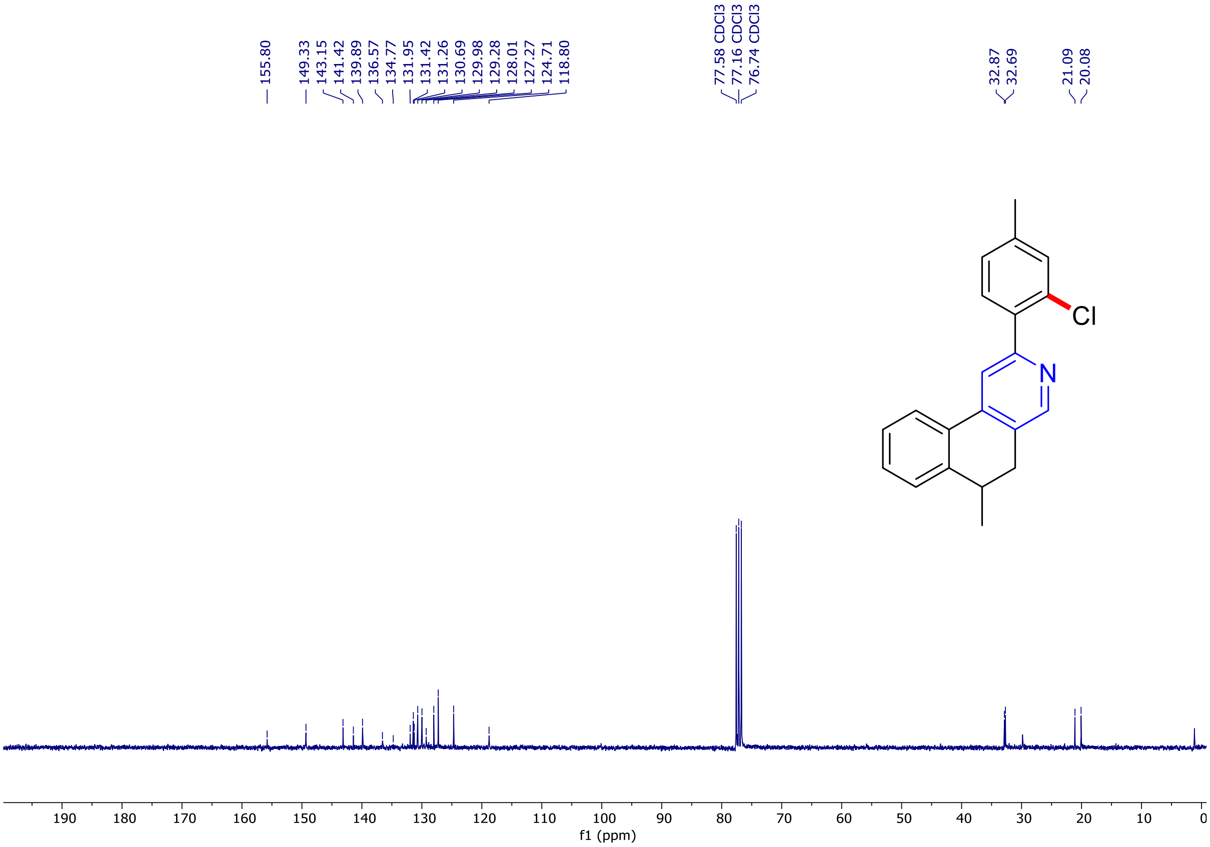

**2-(2-chlorophenyl)-9-methoxy-5,6-dihydrobenzo[f]isoquinoline (4g):**

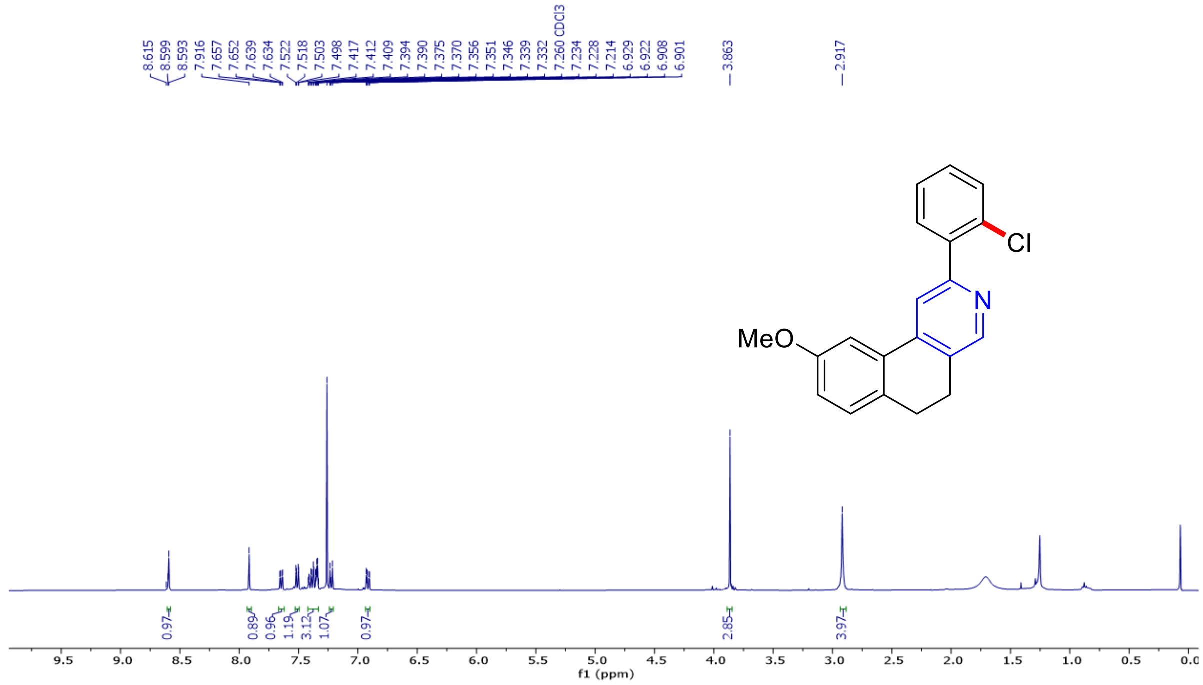


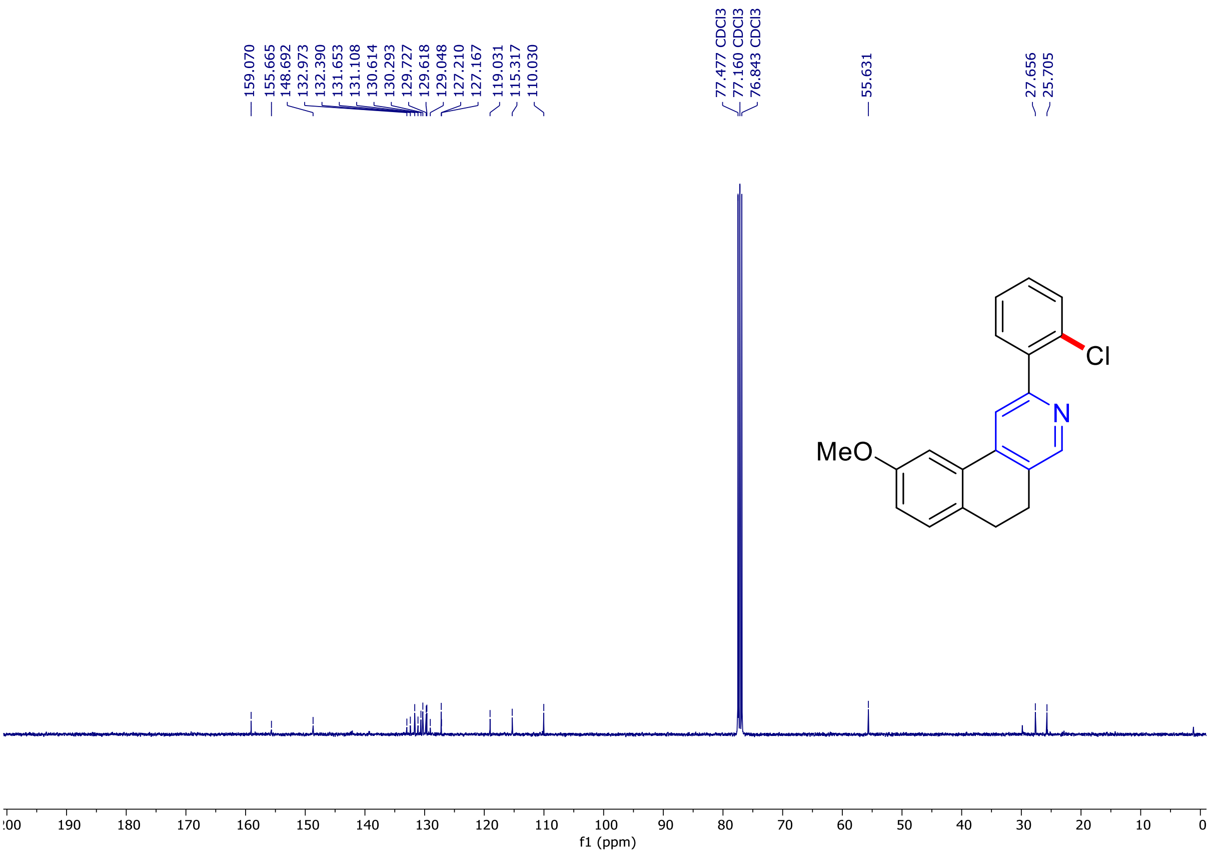

**9-bromo-2-(2,6-dichlorophenyl)-5,6-dihydrobenzo[f]isoquinoline (4h):**

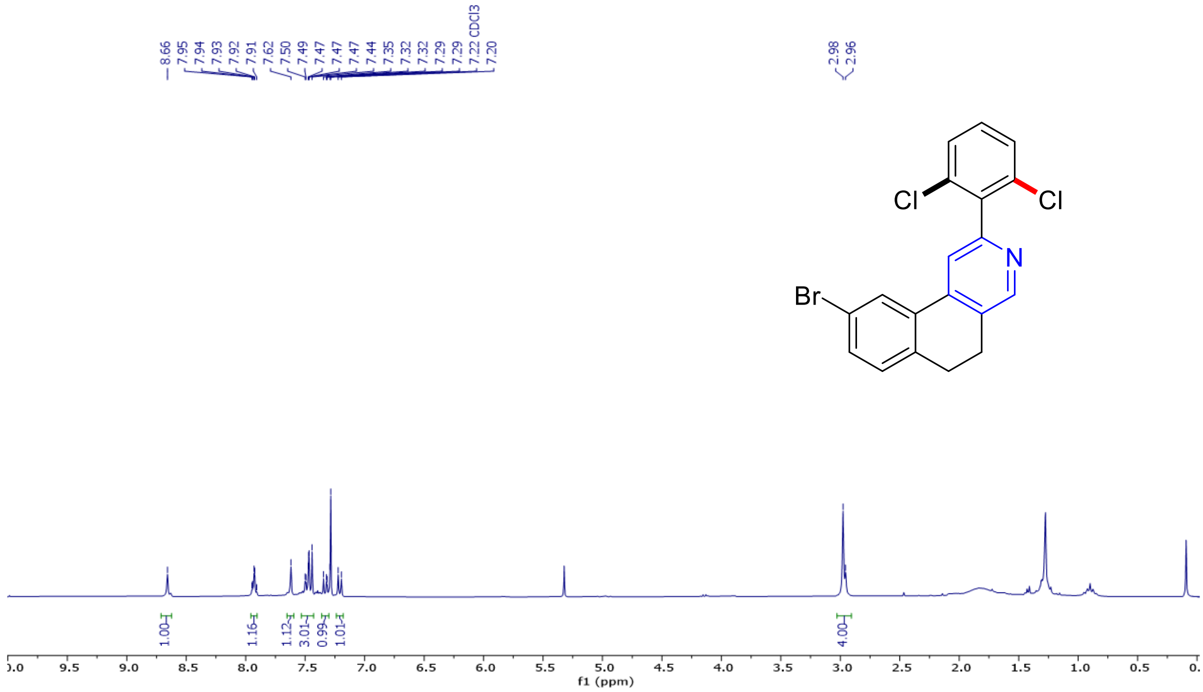


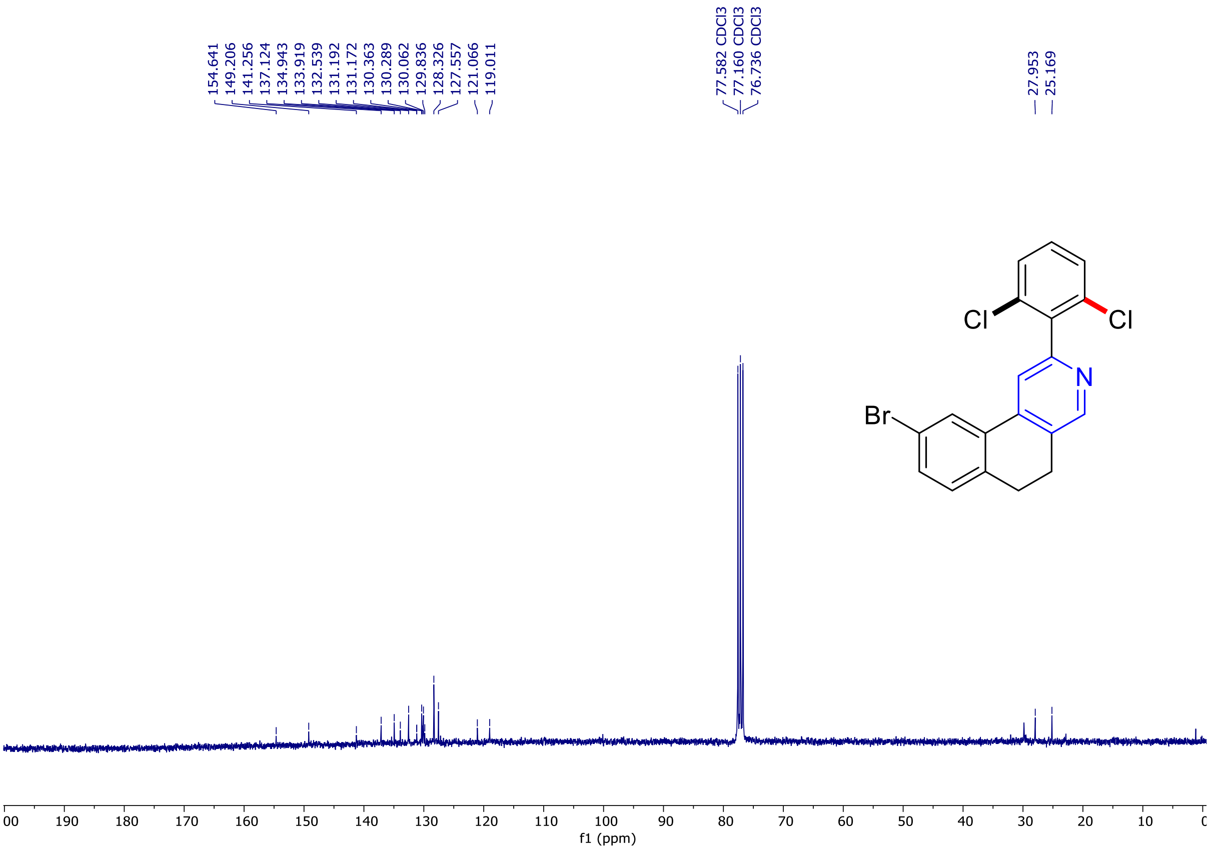

**8-methoxy-2-(2-(phenylselanyl)phenyl)-5,6-dihydrobenzo[f]isoquinoline(5):**

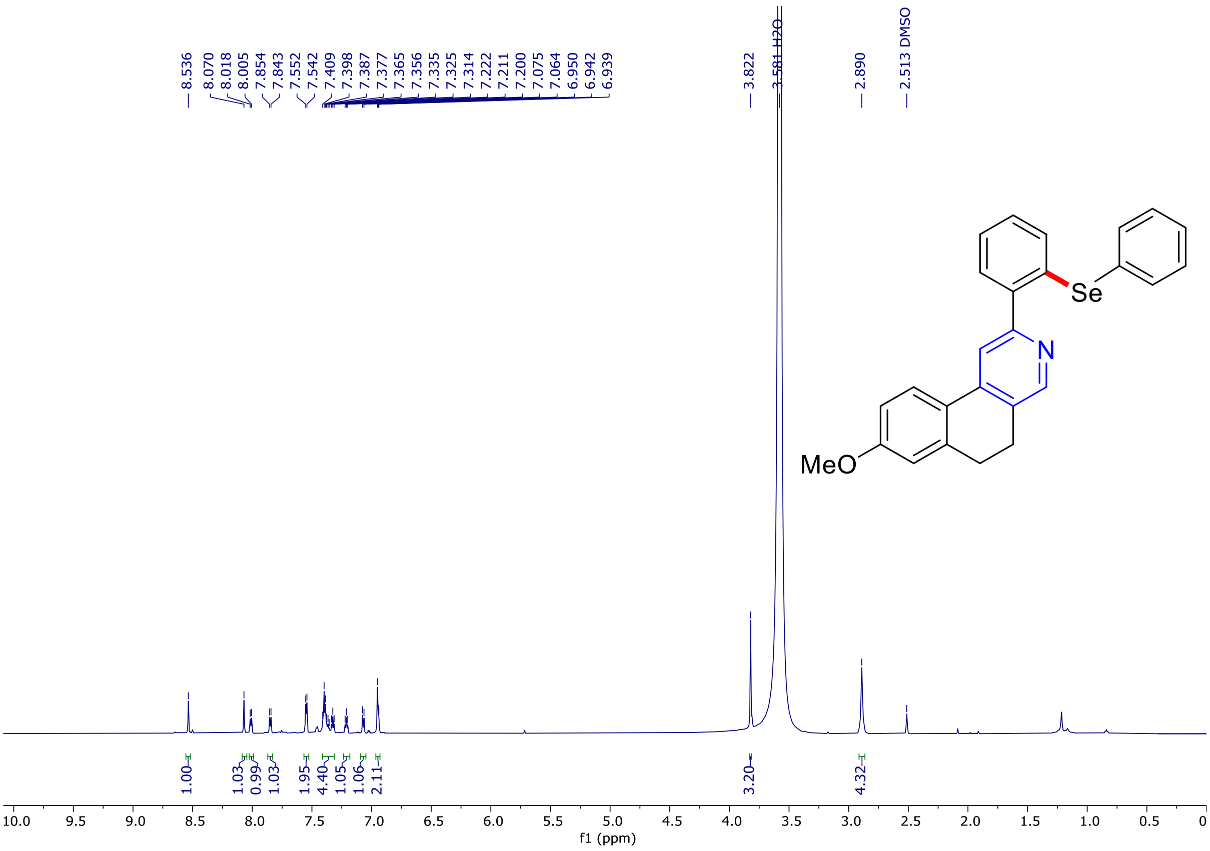


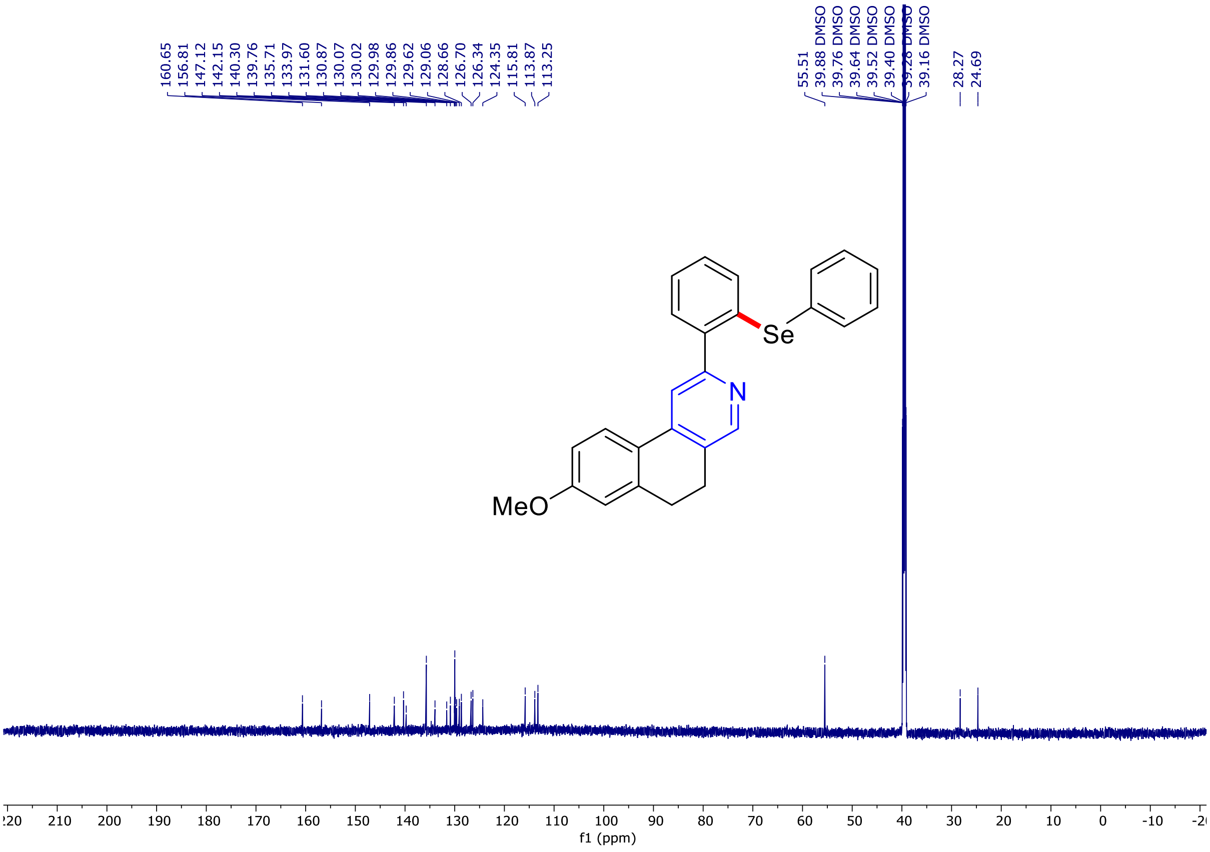

## DFT calculations

Density functional theory (DFT) calculations were carried out using the Gaussian 09 software suite,[1] employing the mPW1PW91 exchange–correlation functional. For light atoms (H, C, O, Se, and N), the D95V basis set was applied, while transition metals (Cu, Pd, and Ru) were treated using the LANL2DZ basis set to account for relativistic effects.[2,3] All molecular systems were optimised in both the singlet ground state and the lowest-energy triplet excited state. Subsequent vibrational frequency analyses confirmed the nature of the stationary points, with all optimised geometries corresponding to true minima, as indicated by the absence of imaginary frequencies.

### Coordinates

<table>
<tr>
<td>
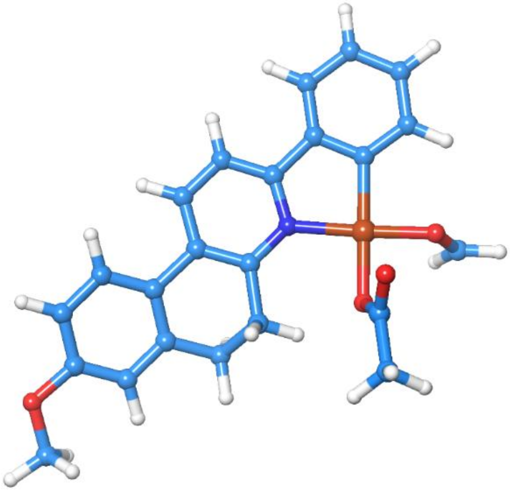
<br>Sum of electronic and thermal Free Energies= -2869.808968
<br>Symbolic Z-matrix:
<br>Charge = 0 Multiplicity = 2
<br>Cu 0.2948 0.30636 0.
<br>O -1.4532 0.09936 -0.501
<br>C -1.6982 -0.01664 -1.762
<br>O 0.1818 2.06236 -0.504
<br>C -0.6232 2.76336 0.457
<br>C 0.4158 -1.55564 0.533
<br>C -0.5932 -2.50964 0.444
</td>
<td>
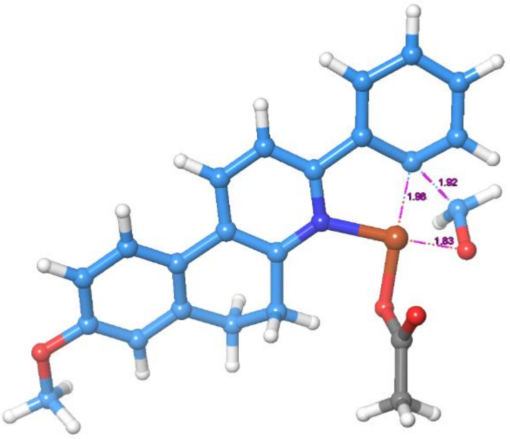

<br>Sum of electronic and thermal Free Energies= -2869.692289
<br>Symbolic Z-matrix:
<br>Charge = 0 Multiplicity = 2
<br>Cu -0.03094 0.34653 0.
<br>O -1.77894 0.13953 -0.501
<br>C -1.20094 -0.86247 -1.065
<br>O -0.14394 2.10253 -0.504
<br>C -0.94894 2.80353 0.457
<br>C 0.07906 -1.62047 0.152
<br>C -0.91894 -2.46947 0.444
</td>
</tr>
</table>

| | | | |
|---|---|---|---|
| C | 1.6408 | -1.86564 | 1.06 |
| C | -0.3402 | -3.80164 | 0.899 |
| H | -1.5452 | -2.25664 | 0.039 |
| C | 1.9068 | -3.15364 | 1.517 |
| C | 0.9068 | -4.12264 | 1.434 |
| H | -1.1012 | -4.54864 | 0.84 |
| H | 2.8558 | -3.40364 | 1.923 |
| H | 1.0948 | -5.11664 | 1.782 |
| C | 2.5808 | -0.74064 | 1.058 |
| C | 3.0428 | 1.47136 | 0.481 |
| C | 3.8588 | -0.93864 | 1.554 |
| C | 4.3528 | 1.30836 | 0.967 |
| C | 4.7568 | 0.10336 | 1.518 |
| H | 4.1498 | -1.88164 | 1.95 |
| H | 5.7448 | -0.02964 | 1.887 |
| N | 2.1518 | 0.43436 | 0.547 |
| C | 2.7518 | 2.89236 | -0.075 |
| H | 1.9108 | 2.89536 | -0.728 |
| H | 2.5458 | 3.54836 | 0.781 |
| C | 3.9438 | 3.48236 | -0.823 |
| H | 3.7338 | 4.49636 | -1.202 |
| H | 4.1478 | 2.82136 | -1.678 |
| C | 5.1448 | 3.48836 | 0.032 |
| C | 5.3598 | 2.42336 | 0.895 |
| C | 6.0648 | 4.52836 | -0.07 |
| C | 6.5238 | 2.40136 | 1.661 |
| C | 7.2268 | 4.50936 | 0.704 |

| | | | |
|---|---|---|---|
| C | 1.31506 | -1.82547 | 1.06 |
| C | -0.66594 | -3.76147 | 0.899 |
| H | -1.87094 | -2.21647 | 0.039 |
| C | 1.58106 | -3.11347 | 1.517 |
| C | 0.58106 | -4.08247 | 1.434 |
| H | -1.42694 | -4.50847 | 0.84 |
| H | 2.53006 | -3.36347 | 1.923 |
| H | 0.76906 | -5.07647 | 1.782 |
| C | 2.25506 | -0.70047 | 1.058 |
| C | 2.71706 | 1.51153 | 0.481 |
| C | 3.53306 | -0.89847 | 1.554 |
| C | 4.02706 | 1.34853 | 0.967 |
| C | 4.43106 | 0.14353 | 1.518 |
| H | 3.82406 | -1.84147 | 1.95 |
| H | 5.41906 | 0.01053 | 1.887 |
| N | 1.82606 | 0.47453 | 0.547 |
| C | 2.42606 | 2.93253 | -0.075 |
| H | 1.58506 | 2.93553 | -0.728 |
| H | 2.22006 | 3.58853 | 0.781 |
| C | 3.61806 | 3.52253 | -0.823 |
| H | 3.40806 | 4.53653 | -1.202 |
| H | 3.82206 | 2.86153 | -1.678 |
| C | 4.81906 | 3.52853 | 0.032 |
| C | 5.03406 | 2.46353 | 0.895 |
| C | 5.73906 | 4.56853 | -0.07 |
| C | 6.19806 | 2.44153 | 1.661 |
| C | 6.90106 | 4.54953 | 0.704 |

<table>
<tr>
<td>C 7.4448 3.43936 1.567<br>H 6.7178 1.59436 2.327<br>O 8.2238 5.52936 0.657<br>H 8.3298 3.41236 2.16<br>H 5.8668 5.31636 -0.749<br>C 7.9768 6.61736 -0.259<br>H 8.8118 7.33136 -0.209<br>H 7.0428 7.12836 0.015<br>H 7.8958 6.22536 -1.285<br>C -0.9992 4.23336 0.277<br>O -0.9752 2.14936 1.465<br>H -1.4262 4.65536 1.21<br>H -1.7492 4.30836 -0.532<br>H -0.1172 4.80136 -0.057<br>H -0.9072 0.00436 -2.468<br>H -2.7172 -0.14264 -2.105</td>
<td>C 7.11906 3.47953 1.567<br>H 6.39206 1.63453 2.327<br>O 7.89806 5.56953 0.657<br>H 8.00406 3.45253 2.16<br>H 5.54106 5.35653 -0.749<br>C 7.65106 6.65753 -0.259<br>H 8.48606 7.37153 -0.209<br>H 6.71706 7.16853 0.015<br>H 7.57006 6.26553 -1.285<br>C -1.32494 4.27353 0.277<br>O -1.30094 2.18953 1.465<br>H -1.75194 4.69553 1.21<br>H -2.07494 4.34853 -0.532<br>H -0.44294 4.84153 -0.057<br>H -0.69194 -0.49547 -1.923<br>H -1.93894 -1.57647 -1.358</td>
</tr>
<tr>
<td>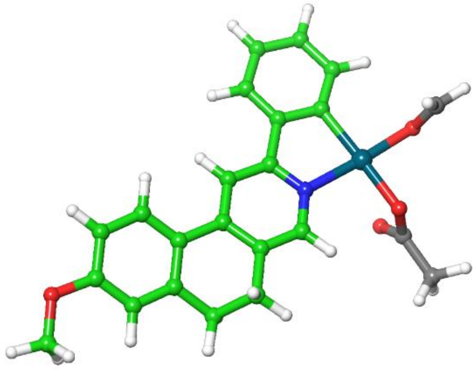<br>Sum of electronic and thermal Free Energies= -6154.856482<br>Symbolic Z-matrix:</td>
<td>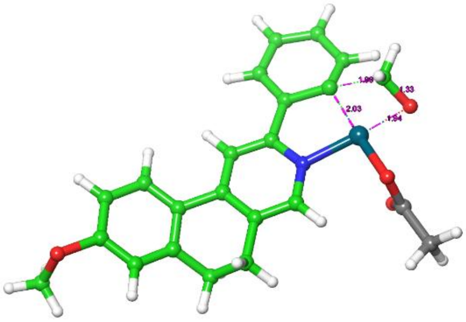<br>Sum of electronic and thermal Free Energies= -6154.700775<br>Symbolic Z-matrix:</td>
</tr>
</table>

| | |
|---|---|
| Charge = 0 Multiplicity = 1 | Charge = 0 Multiplicity = 1 |
| Pd -0.3651 0.37129 0. | Pd 0.08045 -0.24752 0. |
| O -0.8501 1.96729 -0.991 | O -0.40455 1.34848 -0.991 |
| O 1.4339 0.48329 -0.718 | O 1.87945 -0.13552 -0.718 |
| C -2.1991 0.23229 0.891 | C -1.67955 -0.11852 0.997 |
| C -3.2861 1.08229 0.709 | C -2.84755 0.43348 0.615 |
| C -2.2741 -0.85971 1.744 | C -1.83455 -1.40652 1.839 |
| C -4.4651 0.82229 1.406 | C -4.03255 0.24848 1.35 |
| H -3.2231 1.91429 0.051 | H -2.78655 1.19048 -0.139 |
| C -3.4511 -1.11671 2.441 | C -2.99655 -1.58652 2.592 |
| C -4.5461 -0.27071 2.267 | C -4.10255 -0.75152 2.345 |
| H -5.3151 1.45929 1.281 | H -4.88255 0.86848 1.157 |
| H -3.5231 -1.94971 3.097 | H -3.04755 -2.34952 3.341 |
| H -5.4561 -0.46071 2.796 | H -5.00055 -0.87552 2.913 |
| C 1.1769 -1.99071 1.086 | C 1.63745 -2.69752 0.993 |
| C -1.0131 -1.70771 1.835 | C -0.56755 -2.29052 1.951 |
| C 1.3219 -3.11971 1.888 | C 1.78145 -3.81352 1.835 |
| H 1.9919 -1.66271 0.492 | H 2.40845 -2.43452 0.299 |
| C -0.9261 -2.82571 2.655 | C -0.43355 -3.37352 2.824 |
| C 0.2619 -3.54871 2.681 | C 0.74745 -4.13352 2.777 |
| H -1.7541 -3.12171 3.251 | H -1.21455 -3.61852 3.514 |
| C 2.6369 -3.85471 1.948 | C 3.05845 -4.70252 1.748 |
| H 3.2609 -3.62271 1.071 | H 3.46845 -4.65852 0.761 |
| H 3.1599 -3.51671 2.855 | H 3.80245 -4.38352 2.447 |
| C 2.3969 -5.35771 2.107 | C 2.58645 -6.11052 2.056 |
| H 3.3399 -5.92771 2.195 | H 3.36145 -6.84152 1.951 |
| H 1.8619 -5.69171 1.206 | H 1.79945 -6.32652 1.365 |

| | | | | | | | |
|---|---|---|---|---|---|---|---|
| C | 1.4949 | -5.61771 | 3.286 | C | 2.04345 | -6.13652 | 3.487 |
| C | 0.4379 | -4.74971 | 3.543 | C | 0.97745 | -5.30452 | 3.757 |
| C | 1.6989 | -6.74171 | 4.081 | C | 2.57745 | -6.94852 | 4.489 |
| C | -0.4291 | -5.02171 | 4.597 | C | 0.19645 | -5.50252 | 4.898 |
| C | 0.8369 | -7.00871 | 5.148 | C | 1.86845 | -7.08652 | 5.695 |
| H | 2.5099 | -7.38371 | 3.854 | H | 3.49945 | -7.46952 | 4.336 |
| C | -0.2251 | -6.14271 | 5.393 | C | 0.64545 | -6.40652 | 5.875 |
| H | -1.2441 | -4.37171 | 4.806 | H | -0.71755 | -4.96252 | 5.034 |
| H | -0.8891 | -6.33971 | 6.202 | H | 0.06545 | -6.57252 | 6.758 |
| N | 0.0229 | -1.31871 | 1.082 | N | 0.46845 | -1.93752 | 1.082 |
| C | -0.6031 | 3.12729 | -0.482 | C | -0.90155 | 1.54948 | 0.23 |
| H | -0.1501 | 3.22529 | 0.473 | H | -0.12355 | 1.89748 | 0.867 |
| H | -0.8611 | 4.00729 | -1.034 | H | -1.65355 | 2.30448 | 0.173 |
| C | 1.4689 | -0.22371 | -1.943 | C | 1.92145 | -0.82152 | -1.957 |
| O | 0.4529 | -0.85471 | -2.246 | O | 0.91045 | -1.44852 | -2.282 |
| C | 2.7239 | -0.28171 | -2.827 | C | 3.18045 | -0.85352 | -2.838 |
| C | 2.0899 | -9.01371 | 5.743 | C | 2.20845 | -9.46652 | 6.434 |
| H | 3.0319 | -8.45271 | 5.831 | H | 2.89645 | -9.73852 | 5.661 |
| H | 2.0889 | -9.83871 | 6.47 | H | 2.37345 | -10.08252 | 7.293 |
| H | 1.9979 | -9.42371 | 4.727 | H | 1.20645 | -9.60452 | 6.084 |
| O | 0.9759 | -8.13771 | 6.015 | O | 2.42045 | -7.98952 | 6.814 |
| H | 2.5129 | -0.79571 | -3.783 | H | 2.96945 | -1.32652 | -3.815 |
| H | 3.5119 | -0.81171 | -2.278 | H | 3.96045 | -1.41652 | -2.309 |
| H | 3.1039 | 0.74229 | -3.006 | H | 3.57045 | 0.17348 | -2.971 |

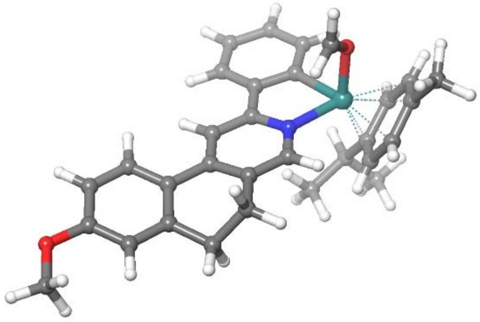

Sum of electronic and thermal Free Energies= -5820.309479

Symbolic Z-matrix:

Charge = 0 Multiplicity = 2

Ru 0.27847 -0.70545 0.

C -0.59553 -0.06945 1.786

C 0.20847 -0.86345 -2.222

C 0.99047 -1.89945 -1.678

H 1.88147 -2.03745 -1.976

C 0.42347 -2.73345 -0.674

H 0.93847 -3.45145 -0.324

C -0.86253 -2.52345 -0.192

C -1.63453 -1.46845 -0.804

H -2.52353 -1.31645 -0.508

C -1.11453 -0.67545 -1.804

H -1.65153 -0.00445 -2.209

C 0.76647 -0.01145 -3.309

H 0.68847 -0.56645 -4.255

H 0.19347 0.92355 -3.396

H 1.83347 0.17355 -3.121

C -1.53853 -3.34545 0.883

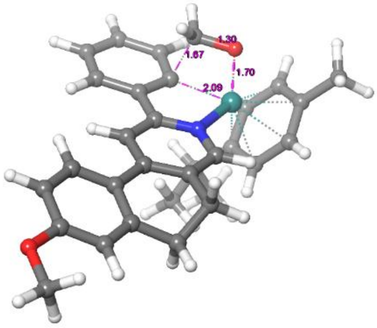


Sum of electronic and thermal Free Energies= -5820.260976

Symbolic Z-matrix:

Charge = 0 Multiplicity = 2

Ru -1.307 2.589 7.576

C -2.181 3.225 9.362

C -1.377 2.431 5.354

C -0.595 1.395 5.898

H 0.296 1.257 5.6

C -1.162 0.561 6.902

H -0.647 -0.157 7.252

C -2.448 0.771 7.384

C -3.22 1.826 6.772

H -4.109 1.978 7.068

C -2.7 2.619 5.772

H -3.237 3.29 5.367

C -0.819 3.283 4.267

H -0.897 2.728 3.321

H -1.392 4.218 4.18

H 0.248 3.468 4.455

C -3.124 -0.051 8.459

| | |
|---|---|
| H -2.20853 -2.69345 1.463 | H -3.794 0.601 9.039 |
| C -2.37153 -4.45445 0.233 | C -3.957 -1.16 7.809 |
| H -2.87153 -5.03945 1.016 | H -4.457 -1.745 8.592 |
| H -3.12453 -4.00845 -0.431 | H -4.71 -0.714 7.145 |
| H -1.70953 -5.10945 -0.35 | H -3.295 -1.815 7.226 |
| C -0.50953 -4.02545 1.818 | C -2.095 -0.731 9.394 |
| H 0.11747 -3.25945 2.294 | H -1.468 0.035 9.87 |
| H -1.04553 -4.59445 2.593 | H -2.631 -1.3 10.169 |
| H 0.12347 -4.71345 1.239 | H -1.462 -1.419 8.815 |
| C -1.87653 0.46955 1.885 | C -3.462 3.764 9.461 |
| H -2.49053 0.58055 1.019 | H -4.076 3.875 8.595 |
| C -2.35653 0.86655 3.129 | C -3.942 4.161 10.705 |
| H -3.33453 1.27955 3.213 | H -4.92 4.574 10.789 |
| C -1.56053 0.72455 4.263 | C -3.146 4.019 11.839 |
| H -1.93453 1.03355 5.219 | H -3.52 4.328 12.795 |
| C -0.27953 0.18855 4.152 | C -1.865 3.483 11.728 |
| H 0.32747 0.08655 5.015 | H -1.258 3.381 12.591 |
| C 0.18847 -0.20345 2.899 | C -1.397 3.091 10.475 |
| C 1.47847 -0.75645 2.62 | C -0.107 2.538 10.196 |
| C 2.41047 -0.95845 3.635 | C 0.825 2.336 11.211 |
| H 2.17447 -0.70745 4.643 | H 0.589 2.587 12.219 |
| C 3.64947 -1.50145 3.317 | C 2.064 1.793 10.893 |
| C 3.92147 -1.80745 1.989 | C 2.336 1.487 9.565 |
| C 2.95347 -1.57845 1.012 | C 1.368 1.716 8.588 |
| H 3.17447 -1.81045 -0.001 | H 1.589 1.484 7.575 |
| N 1.75547 -1.06945 1.337 | N 0.17 2.225 8.913 |
| C 5.29447 -2.34645 1.621 | C 3.709 0.948 9.197 |

| | |
|---|---|
| H 5.28447 -2.82745 0.63 | H 3.699 0.467 8.206 |
| H 5.99347 -1.49545 1.598 | H 4.408 1.799 9.174 |
| C 5.79847 -3.29045 2.714 | C 4.213 0.004 10.29 |
| H 6.81247 -3.67445 2.502 | H 5.227 -0.38 10.078 |
| H 5.09947 -4.13645 2.756 | H 3.514 -0.842 10.332 |
| C 5.75347 -2.60245 4.053 | C.168 0.692 11.629 |
| C 4.69247 -1.75245 4.353 | C.107 1.542 11.929 |
| C 6.74747 -2.85645 4.993 | C 5.162 0.438 12.569 |
| H 7.53647 -3.51245 4.733 | H 5.951 -0.218 12.309 |
| C 6.68647 -2.25645 6.254 | C 5.101 1.038 13.83 |
| C 5.61947 -1.41445 6.552 | C 4.034 1.88 14.128 |
| H 5.56347 -0.95645 7.512 | H 3.978 2.338 15.087 |
| C 4.62447 -1.16345 5.613 | C 3.039 2.131 13.189 |
| H 3.82047 -0.51545 5.863 | H 2.235 2.779 13.439 |
| O 0.61547 1.10455 -0.515 | O -1.37 4.285 7.433 |
| C 1.80547 1.58655 -0.475 | C -1.743 4.654 8.626 |
| H 2.62347 0.98955 -0.176 | H -0.947 5.134 9.149 |
| H 1.97347 2.60055 -0.769 | H -2.589 5.331 8.561 |
| O 7.66547 -2.46345 7.277 | O 6.08 0.831 14.853 |
| C 8.76047 -3.34045 6.939 | C 7.175 -0.046 14.515 |
| H 9.43947 -3.42145 7.8 | H 7.854 -0.127 15.376 |
| H 9.31047 -2.93145 6.079 | H 7.725 0.363 13.655 |
| H 8.37047 -4.33845 6.688 | H 6.786 -1.044 14.264 |

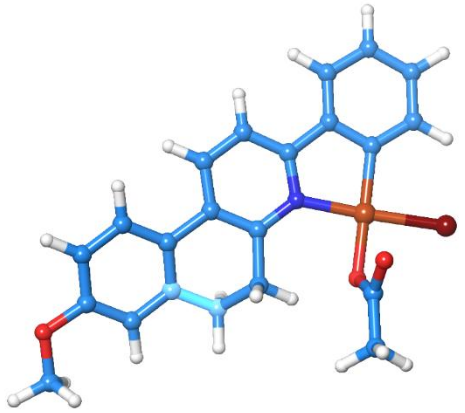

Sum of electronic and thermal Free Energies= -5317.853473

Symbolic Z-matrix:

Charge = 0 Multiplicity = 1

| | | | |
|---|---|---|---|
| Cu | 0.24134 | -0.29703 | 0. |
| Br | -1.96466 | -0.55803 | -0.632 |
| O | 0.12834 | 1.45897 | -0.504 |
| C | -0.63266 | 2.18297 | 0.474 |
| C | 0.38734 | -2.16503 | 0.476 |
| C | -0.58766 | -3.15803 | 0.357 |
| C | 1.61334 | -2.47003 | 1.011 |
| C | -0.30466 | -4.45303 | 0.782 |
| H | -1.54066 | -2.94103 | -0.05 |
| C | 1.90834 | -3.76103 | 1.44 |
| C | 0.94034 | -4.75503 | 1.325 |
| H | -1.04666 | -5.21603 | 0.694 |
| H | 2.86034 | -3.99503 | 1.849 |
| H | 1.15234 | -5.75303 | 1.65 |
| C | 2.53234 | -1.34403 | 1.046 |
| C | 2.98734 | 0.87197 | 0.504 |

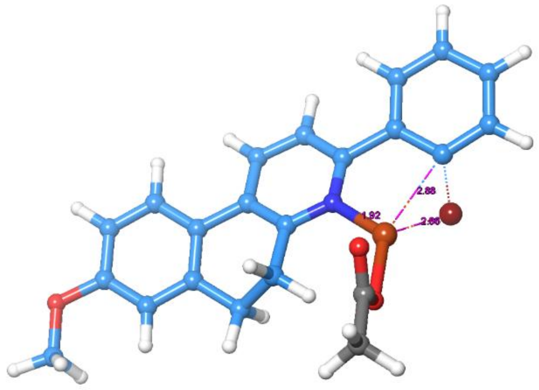

Sum of electronic and thermal Free Energies= -5317.716572

Symbolic Z-matrix:

Charge = 0 Multiplicity = 1

| | | | |
|---|---|---|---|
| Cu | -0.19183 | -0.2104 | 0. |
| Br | 0.00617 | -2.6084 | -1.552 |
| O | -0.72883 | 1.5136 | -0.577 |
| C | -1.44983 | 2.2146 | 0.447 |
| C | -0.27983 | -3.0794 | 0.176 |
| C | -1.10583 | -4.2454 | 0.235 |
| C | 0.93617 | -3.0854 | 0.936 |
| C | -0.76483 | -5.3364 | 1.019 |
| H | -2.00083 | -4.2874 | -0.349 |
| C | 1.25017 | -4.2114 | 1.705 |
| C | 0.41117 | -5.3194 | 1.751 |
| H | -1.39883 | -6.1944 | 1.044 |
| H | 2.14517 | -4.2344 | 2.269 |
| H | 0.67517 | -6.1624 | 2.343 |
| C | 1.93517 | -1.8814 | 0.91 |
| C | 2.53617 | 0.3436 | 0.511 |

| | | | | | | | |
|---|---|---|---|---|---|---|---|
| C | 3.80534 | -1.54003 | 1.557 | C | 3.23217 | -2.1454 | 1.351 |
| C | 4.29034 | 0.71597 | 1.012 | C | 3.84517 | 0.1146 | 0.932 |
| C | 4.69534 | -0.49203 | 1.555 | C | 4.19417 | -1.1504 | 1.368 |
| H | 4.09834 | -2.48603 | 1.943 | H | 3.49817 | -3.1194 | 1.675 |
| H | 5.67734 | -0.62203 | 1.941 | H | 5.18317 | -1.3624 | 1.702 |
| N | 2.09834 | -0.16903 | 0.547 | N | 1.61017 | -0.6294 | 0.507 |
| C | 2.70234 | 2.28997 | -0.042 | C | 2.17817 | 1.7476 | 0.104 |
| H | 1.87734 | 2.28797 | -0.72 | H | 1.27817 | 1.7536 | -0.486 |
| H | 2.46034 | 2.93297 | 0.816 | H | 2.01217 | 2.3326 | 1.021 |
| C | 3.90334 | 2.90897 | -0.748 | C | 3.32217 | 2.4076 | -0.649 |
| H | 3.68534 | 3.92397 | -1.117 | H | 3.08217 | 3.4416 | -0.941 |
| H | 4.14734 | 2.26397 | -1.608 | H | 3.49717 | 1.8096 | -1.558 |
| C | 5.07534 | 2.92297 | 0.139 | C | 4.56917 | 2.3626 | 0.167 |
| C | 5.28434 | 1.84497 | 0.986 | C | 4.83917 | 1.2296 | 0.925 |
| C | 5.97634 | 3.98197 | 0.087 | C | 5.46017 | 3.4286 | 0.132 |
| C | 6.42534 | 1.82697 | 1.786 | C | 6.02617 | 1.1636 | 1.65 |
| C | 7.11434 | 3.96997 | 0.897 | C | 6.64517 | 3.3696 | 0.867 |
| C | 7.32834 | 2.88397 | 1.741 | C | 6.91817 | 2.2306 | 1.621 |
| H | 6.61334 | 1.00997 | 2.441 | H | 6.25517 | 0.3036 | 2.235 |
| O | 8.09034 | 5.01097 | 0.905 | O | 7.61117 | 4.4206 | 0.884 |
| H | 8.19534 | 2.86197 | 2.361 | H | 7.82317 | 2.1756 | 2.183 |
| H | 5.78334 | 4.78197 | -0.58 | H | 5.21917 | 4.2706 | -0.463 |
| C | 7.84234 | 6.11997 | 0.014 | C | 7.29917 | 5.5826 | 0.081 |
| H | 8.65934 | 6.85097 | 0.108 | H | 8.10917 | 6.3196 | 0.175 |
| H | 6.89034 | 6.60297 | 0.279 | H | 6.35617 | 6.0326 | 0.427 |
| H | 7.79634 | 5.75797 | -1.024 | H | 7.20017 | 5.2846 | -0.974 |
| C | -1.00866 | 3.64797 | 0.28 | C | -2.10683 | 3.5886 | 0.197 |

| | |
|---|---|
| O -0.95566 1.58397 1.501<br>H -1.42666 4.07897 1.21<br>H -1.76266 3.71497 -0.526<br>H -0.12766 4.20797 -0.067 | O -1.51683 1.6866 1.56<br>H -2.58583 3.9676 1.115<br>H -2.84583 3.4866 -0.612<br>H -1.34683 4.2956 -0.167 |
| 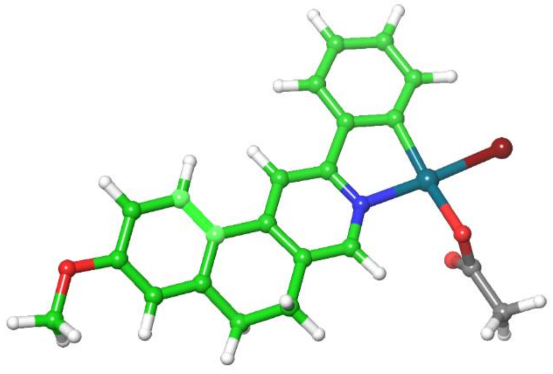<br>Sum of electronic and thermal Free Energies= -8602.861482<br>Symbolic Z-matrix:<br>Charge = 0 Multiplicity = 2<br>Pd -0.51361 -0.19802 0.<br>Br -1.11361 1.77598 -1.226<br>O 1.28539 -0.08602 -0.718<br>C -2.27361 -0.06902 0.997<br>C -3.29161 0.87198 0.886<br>C -2.35761 -1.13302 1.896<br>C -4.41561 0.73798 1.696<br>H -3.22161 1.67698 0.2<br>C -3.48461 -1.25902 2.703<br>C -4.51061 -0.32002 2.599<br>H -5.20761 1.44898 1.626 | 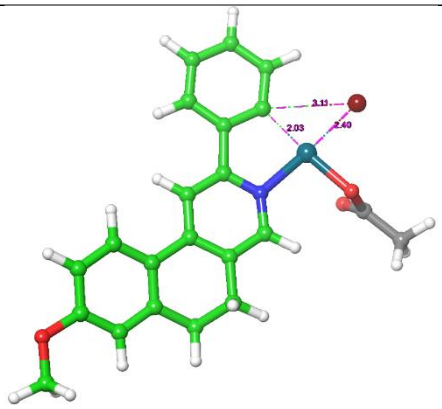<br><br>Sum of electronic and thermal Free Energies= -8602.843188<br>Symbolic Z-matrix:<br>Charge = 0 Multiplicity = 2<br>Pd -0.35269 -0.32178 -0.00462<br>Br -1.29042 1.64403 -1.01258<br>O 1.44631 -0.20978 -0.72262<br>C -2.11269 -0.19278 0.99238<br>C -3.13069 0.74822 0.88138<br>C -2.19669 -1.25678 1.89138<br>C -4.25469 0.61422 1.69138<br>H -3.06069 1.55322 0.19538<br>C -3.32369 -1.38278 2.69838<br>C -4.34969 -0.44378 2.59438<br>H -5.04669 1.32522 1.62138 |

| | | | |
|---|---|---|---|
| H | -3.56961 -2.06102 3.395 | H | -3.40869 -2.18478 3.39038 |
| H | -5.37861 -0.41002 3.215 | H | -5.21769 -0.53378 3.21038 |
| C | 0.96639 -2.63902 1.009 | C | 1.12731 -2.76278 1.00438 |
| C | -1.13361 -2.13302 1.925 | C | -0.97269 -2.25678 1.92038 |
| C | 1.08039 -3.74602 1.845 | C | 1.24131 -3.86978 1.84038 |
| H | 1.74539 -2.38802 0.334 | H | 1.90631 -2.51178 0.32938 |
| C | -1.06961 -3.22502 2.782 | C | -0.90869 -3.34878 2.77738 |
| C | 0.05439 -4.04802 2.738 | C | 0.21531 -4.17178 2.73338 |
| H | -1.86461 -3.42502 3.458 | H | -1.70369 -3.54878 3.45338 |
| C | 2.32939 -4.59202 1.834 | C | 2.49031 -4.71578 1.82938 |
| H | 2.89539 -4.45102 0.9 | H | 3.05631 -4.57478 0.89538 |
| H | 2.95339 -4.26702 2.679 | H | 3.11431 -4.39078 2.67438 |
| C | 1.97639 -6.06002 2.079 | C | 2.13731 -6.18378 2.07438 |
| H | 2.87139 -6.70802 2.119 | H | 3.03231 -6.83178 2.11438 |
| H | 1.34139 -6.38002 1.241 | H | 1.50231 -6.50378 1.23638 |
| C | 1.15639 -6.19402 3.337 | C | 1.31731 -6.31778 3.33238 |
| C | 0.20139 -5.22702 3.637 | C | 0.36231 -5.35078 3.63238 |
| C | 1.33139 -7.30002 4.163 | C | 1.49231 -7.42378 4.15838 |
| C | -0.59461 -5.38202 4.769 | C | -0.43369 -5.50578 4.76438 |
| C | 0.54339 -7.44902 5.307 | C | 0.70431 -7.57278 5.30238 |
| H | 2.06439 -8.01802 3.902 | H | 2.22531 -8.14178 3.89738 |
| C | -0.41861 -6.48502 5.597 | C | -0.25769 -6.60878 5.59238 |
| H | -1.33161 -4.65602 5.013 | H | -1.17069 -4.77978 5.00838 |
| H | -1.02661 -6.59202 6.464 | H | -0.86569 -6.71578 6.45938 |
| N | -0.12561 -1.88802 1.082 | N | 0.03531 -2.01178 1.07738 |
| C | 1.29539 -0.64602 -2.01 | C | 1.45631 -0.76978 -2.01462 |
| O | 0.26239 -1.20002 -2.394 | O | 0.42331 -1.32378 -2.39862 |

| | |
|---|---|
| C 2.53739 -0.59902 -2.895 | C 2.69831 -0.72278 -2.89962 |
| C 1.66939 -9.53102 5.892 | C 1.83031 -9.65478 5.88738 |
| H 2.65939 -9.05002 5.879 | H 2.82031 -9.17378 5.87438 |
| H 1.66139 -10.32402 6.654 | H 1.82231 -10.44778 6.64938 |
| H 1.45939 -9.97102 4.906 | H 1.62031 -10.09478 4.90138 |
| O 0.65939 -8.55102 6.211 | O 0.82031 -8.67478 6.20638 |
| H 2.36339 -1.11402 -3.859 | H 2.52431 -1.23778 -3.86362 |
| H 3.38039 -1.05402 -2.354 | H 3.54131 -1.17778 -2.35862 |
| H 2.81739 0.45698 -3.071 | H 2.97831 0.33322 -3.07562 |
| 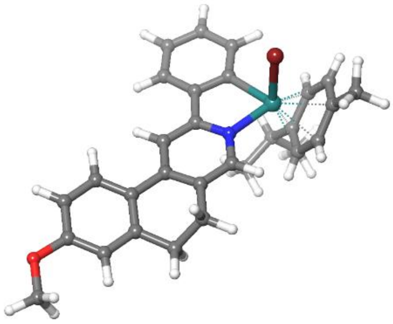 | 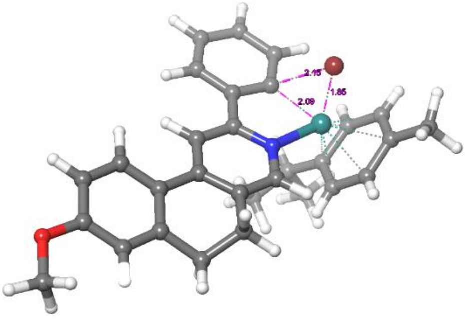 |
| Sum of electronic and thermal Free Energies= -8268.589906 | Sum of electronic and thermal Free Energies= -8268.420985 |
| Symbolic Z-matrix: | Symbolic Z-matrix: |
| Charge = 0 Multiplicity = 1 | Charge = 0 Multiplicity = 1 |
| Ru 0.09282 0.61881 0. | Ru -0.3651 -0.70545 0. |
| C -0.78118 1.25481 1.786 | C -1.2391 -0.06945 1.786 |
| C 0.02282 0.46081 -2.222 | C -0.4351 -0.86345 -2.222 |
| C 0.80482 -0.57519 -1.678 | C 0.3469 -1.89945 -1.678 |
| H 1.69582 -0.71319 -1.976 | H 1.2379 -2.03745 -1.976 |
| C 0.23782 -1.40919 -0.674 | C -0.2201 -2.73345 -0.674 |
| H 0.75282 -2.12719 -0.324 | H 0.2949 -3.45145 -0.324 |

| | | | | | | | |
|---|---|---|---|---|---|---|---|
| C | -1.04818 -1.19919 -0.192 | | | C | -1.5061 -2.52345 -0.192 | | |
| C | -1.82018 -0.14419 -0.804 | | | C | -2.2781 -1.46845 -0.804 | | |
| H | -2.70918 0.00781 -0.508 | | | H | -3.1671 -1.31645 -0.508 | | |
| C | -1.30018 0.64881 -1.804 | | | C | -1.7581 -0.67545 -1.804 | | |
| H | -1.83718 1.31981 -2.209 | | | H | -2.2951 -0.00445 -2.209 | | |
| C | 0.58082 1.31281 -3.309 | | | C | 0.1229 -0.01145 -3.309 | | |
| H | 0.50282 0.75781 -4.255 | | | H | 0.0449 -0.56645 -4.255 | | |
| H | 0.00782 2.24781 -3.396 | | | H | -0.4501 0.92355 -3.396 | | |
| H | 1.64782 1.49781 -3.121 | | | H | 1.1899 0.17355 -3.121 | | |
| C | -1.72418 -2.02119 0.883 | | | C | -2.1821 -3.34545 0.883 | | |
| H | -2.39418 -1.36919 1.463 | | | H | -2.8521 -2.69345 1.463 | | |
| C | -2.55718 -3.13019 0.233 | | | C | -3.0151 -4.45445 0.233 | | |
| H | -3.05718 -3.71519 1.016 | | | H | -3.5151 -5.03945 1.016 | | |
| H | -3.31018 -2.68419 -0.431 | | | H | -3.7681 -4.00845 -0.431 | | |
| H | -1.89518 -3.78519 -0.35 | | | H | -2.3531 -5.10945 -0.35 | | |
| C | -0.69518 -2.70119 1.818 | | | C | -1.1531 -4.02545 1.818 | | |
| H | -0.06818 -1.93519 2.294 | | | H | -0.5261 -3.25945 2.294 | | |
| H | -1.23118 -3.27019 2.593 | | | H | -1.6891 -4.59445 2.593 | | |
| H | -0.06218 -3.38919 1.239 | | | H | -0.5201 -4.71345 1.239 | | |
| C | -2.06218 1.79381 1.885 | | | C | -2.5201 0.46955 1.885 | | |
| H | -2.67618 1.90481 1.019 | | | H | -3.1341 0.58055 1.019 | | |
| C | -2.54218 2.19081 3.129 | | | C | -3.0001 0.86655 3.129 | | |
| H | -3.52018 2.60381 3.213 | | | H | -3.9781 1.27955 3.213 | | |
| C | -1.74618 2.04881 4.263 | | | C | -2.2041 0.72455 4.263 | | |
| H | -2.12018 2.35781 5.219 | | | H | -2.5781 1.03355 5.219 | | |
| C | -0.46518 1.51281 4.152 | | | C | -0.9231 0.18855 4.152 | | |
| H | 0.14182 1.41081 5.015 | | | H | -0.3161 0.08655 5.015 | | |

| | | | | | | | |
|---|---|---|---|---|---|---|---|
| C | 0.00282 | 1.12081 | 2.899 | C | -0.4551 | -0.20345 | 2.899 |
| C | 1.29282 | 0.56781 | 2.62 | C | 0.8349 | -0.75645 | 2.62 |
| C | 2.22482 | 0.36581 | 3.635 | C | 1.7669 | -0.95845 | 3.635 |
| H | 1.98882 | 0.61681 | 4.643 | H | 1.5309 | -0.70745 | 4.643 |
| C | 3.46382 | -0.17719 | 3.317 | C | 3.0059 | -1.50145 | 3.317 |
| C | 3.73582 | -0.48319 | 1.989 | C | 3.2779 | -1.80745 | 1.989 |
| C | 2.76782 | -0.25419 | 1.012 | C | 2.3099 | -1.57845 | 1.012 |
| H | 2.98882 | -0.48619 | -0.001 | H | 2.5309 | -1.81045 | -0.001 |
| N | 1.56982 | 0.25481 | 1.337 | N | 1.1119 | -1.06945 | 1.337 |
| C | 5.10882 | -1.02219 | 1.621 | C | 4.6509 | -2.34645 | 1.621 |
| H | 5.09882 | -1.50319 | 0.63 | H | 4.6409 | -2.82745 | 0.63 |
| H | 5.80782 | -0.17119 | 1.598 | H | 5.3499 | -1.49545 | 1.598 |
| C | 5.61282 | -1.96619 | 2.714 | C | 5.1549 | -3.29045 | 2.714 |
| H | 6.62682 | -2.35019 | 2.502 | H | 6.1689 | -3.67445 | 2.502 |
| H | 4.91382 | -2.81219 | 2.756 | H | 4.4559 | -4.13645 | 2.756 |
| C | 5.56782 | -1.27819 | 4.053 | C | 5.1099 | -2.60245 | 4.053 |
| C | 4.50682 | -0.42819 | 4.353 | C | 4.0489 | -1.75245 | 4.353 |
| C | 6.56182 | -1.53219 | 4.993 | C | 6.1039 | -2.85645 | 4.993 |
| H | 7.35082 | -2.18819 | 4.733 | H | 6.8929 | -3.51245 | 4.733 |
| C | 6.50082 | -0.93219 | 6.254 | C | 6.0429 | -2.25645 | 6.254 |
| C | 5.43382 | -0.09019 | 6.552 | C | 4.9759 | -1.41445 | 6.552 |
| H | 5.37782 | 0.36781 | 7.511 | H | 4.9199 | -0.95645 | 7.511 |
| C | 4.43882 | 0.16081 | 5.613 | C | 3.9809 | -1.16345 | 5.613 |
| H | 3.63482 | 0.80881 | 5.863 | H | 3.1769 | -0.51545 | 5.863 |
| O | 7.47982 | -1.13919 | 7.277 | O | 7.0219 | -2.46345 | 7.277 |
| C | 8.57482 | -2.01619 | 6.939 | C | 8.1169 | -3.34045 | 6.939 |
| H | 9.25382 | -2.09719 | 7.8 | H | 8.7959 | -3.42145 | 7.8 |

| | |
|---|---|
| H 9.12482 -1.60719 6.079 | H 8.6669 -2.93145 6.079 |
| H 8.18582 -3.01419 6.688 | H 7.7279 -4.33845 6.688 |
| Br 0.58905 2.87336 -0.57893 | Br -0.96259 1.00492 0.28286 |
| 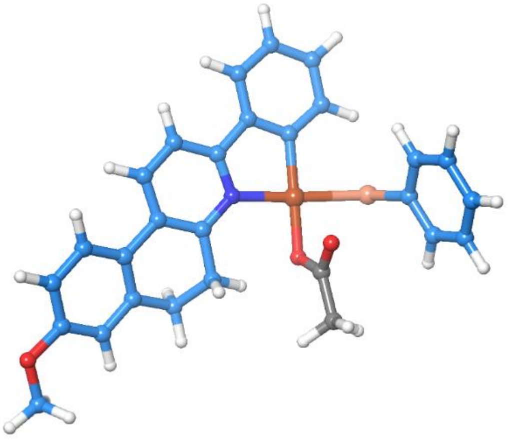 | 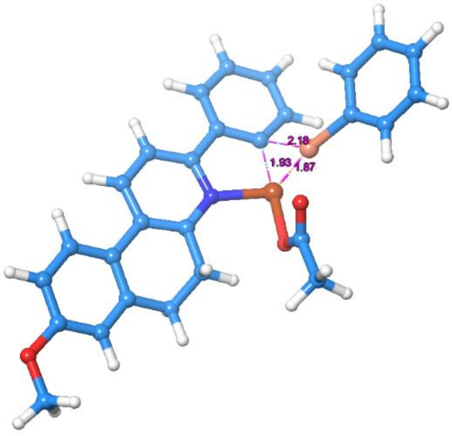 |
| Sum of electronic and thermal Free Energies= -5376.469513 | Sum of electronic and thermal Free Energies= -5376.434601 |
| Symbolic Z-matrix: | Symbolic Z-matrix: |
| Charge = 0 Multiplicity = 1 | Charge = 0 Multiplicity = 1 |
| Cu 0.56312 -0.66832 0. | Cu 0.81064 -0.07426 0. |
| Se -1.67188 -0.93332 -0.641 | Se -0.64216 -0.75771 1.16506 |
| O 0.45012 1.08768 -0.504 | O 0.69764 1.68174 -0.504 |
| C -0.26488 1.83668 0.489 | C 0.05264 2.51374 0.482 |
| C 0.70912 -2.53632 0.476 | C 0.95664 -1.94226 0.476 |
| C -0.27188 -3.51932 0.352 | C 0.09264 -3.04326 0.206 |
| C 1.93412 -2.84432 1.007 | C 2.17464 -2.17226 1.141 |
| C 0.00412 -4.81732 0.773 | C 0.42164 -4.30726 0.688 |
| H -1.22488 -3.29232 -0.054 | H -0.82636 -2.89226 -0.308 |
| C 2.22412 -4.13932 1.432 | C 2.49064 -3.44926 1.605 |
| C 1.25012 -5.12732 1.313 | C 1.60664 -4.50626 1.393 |
| H -0.74088 -5.57732 0.682 | H -0.23836 -5.12926 0.517 |

| | | | | | | | |
|---|---|---|---|---|---|---|---|
| H | 3.17612 | -4.37832 | 1.838 | H | 3.41564 | -3.63426 | 2.093 |
| H | 1.45812 | -6.12832 | 1.634 | H | 1.85164 | -5.48626 | 1.748 |
| C | 2.85612 | -1.71732 | 1.043 | C | 3.07764 | -1.07726 | 1.146 |
| C | 3.30912 | 0.50068 | 0.51 | C | 3.55664 | 1.09474 | 0.51 |
| C | 4.12712 | -1.91432 | 1.555 | C | 4.32964 | -1.25026 | 1.714 |
| C | 4.61112 | 0.34368 | 1.021 | C | 4.84164 | 0.96674 | 1.071 |
| C | 5.01612 | -0.86632 | 1.559 | C | 5.22564 | -0.21126 | 1.685 |
| H | 4.42012 | -2.86232 | 1.938 | H | 4.60364 | -2.17126 | 2.165 |
| H | 5.99712 | -0.99632 | 1.947 | H | 6.19364 | -0.32326 | 2.114 |
| N | 2.42012 | -0.54032 | 0.547 | N | 2.66764 | 0.05374 | 0.547 |
| C | 3.02612 | 1.91968 | -0.03 | C | 3.28864 | 2.48874 | -0.092 |
| H | 2.20412 | 1.91768 | -0.712 | H | 2.48364 | 2.45574 | -0.801 |
| H | 2.77712 | 2.55668 | 0.83 | H | 3.00964 | 3.15474 | 0.738 |
| C | 4.22612 | 2.54668 | -0.725 | C | 4.50464 | 3.10374 | -0.767 |
| H | 4.47612 | 1.90868 | -1.587 | H | 4.78364 | 2.44274 | -1.602 |
| H | 4.00612 | 3.56368 | -1.088 | H | 4.28764 | 4.10774 | -1.167 |
| C | 5.39412 | 2.55968 | 0.167 | C | 5.64764 | 3.15074 | 0.158 |
| C | 5.60212 | 1.47468 | 1.006 | C | 5.83364 | 2.09674 | 1.038 |
| C | 6.29112 | 3.62268 | 0.129 | C | 6.54664 | 4.21274 | 0.108 |
| C | 6.73912 | 1.45568 | 1.812 | C | 6.95064 | 2.10174 | 1.871 |
| C | 7.42412 | 3.60768 | 0.945 | C | 7.65864 | 4.22474 | 0.953 |
| C | 7.63812 | 2.51668 | 1.781 | C | 7.85164 | 3.15974 | 1.828 |
| H | 6.92712 | 0.63368 | 2.462 | H | 7.12264 | 1.30274 | 2.552 |
| O | 8.39412 | 4.65368 | 0.969 | O | 8.62864 | 5.26974 | 0.971 |
| H | 8.50112 | 2.49268 | 2.405 | H | 8.70064 | 3.15474 | 2.473 |
| H | 6.09712 | 4.42768 | -0.532 | H | 6.37164 | 4.99674 | -0.583 |
| C | 8.14612 | 5.77068 | 0.089 | C | 8.39764 | 6.36674 | 0.063 |

| | | | | | | | |
|---|---|---|---|---|---|---|---|
| H | 8.95912 | 6.50468 | 0.194 | H | 9.20964 | 7.10174 | 0.166 |
| H | 7.19012 | 6.24668 | 0.355 | H | 7.43864 | 6.84874 | 0.303 |
| H | 8.10612 | 5.41968 | -0.953 | H | 8.37464 | 5.99274 | -0.971 |
| C | -0.61588 | 3.30768 | 0.286 | C | -0.24836 | 3.98674 | 0.204 |
| O | -0.57288 | 1.25968 | 1.534 | O | -0.20836 | 2.02174 | 1.581 |
| H | -1.05188 | 3.74568 | 1.204 | H | -0.65536 | 4.48974 | 1.101 |
| H | -1.34088 | 3.38968 | -0.546 | H | -0.97436 | 4.05274 | -0.629 |
| H | 0.28412 | 3.85468 | -0.031 | H | 0.66964 | 4.48274 | -0.147 |
| C | -2.90588 | -0.43732 | 0.732 | C | -2.15274 | -0.71641 | 2.13783 |
| C | -3.10188 | -1.24432 | 1.849 | C | -2.57053 | -1.5028 | 3.20259 |
| C | -3.99988 | -0.85732 | 2.837 | C | -3.83558 | -1.2381 | 3.75291 |
| C | -4.70488 | 0.33268 | 2.71 | C | -4.65543 | -0.21749 | 3.26287 |
| C | -4.51788 | 1.13568 | 1.592 | C | -4.22606 | 0.56185 | 2.20735 |
| C | -3.61988 | 0.74968 | 0.604 | C | -2.98626 | 0.30114 | 1.6667 |
| H | -2.57388 | -2.15132 | 1.953 | H | -1.9672 | -2.26176 | 3.59138 |
| H | -4.14688 | -1.47132 | 3.69 | H | -4.18337 | -1.81274 | 4.56164 |
| H | -5.38588 | 0.62668 | 3.463 | H | -5.60797 | -0.0259 | 3.69205 |
| H | -5.05788 | 2.04268 | 1.493 | H | -4.83278 | 1.35659 | 1.81466 |
| H | -3.47588 | 1.36468 | -0.248 | H | -2.65109 | 0.91105 | 0.85478 |

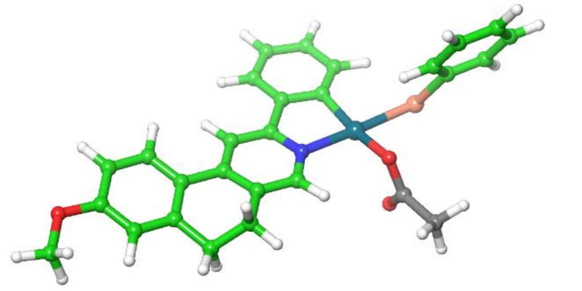

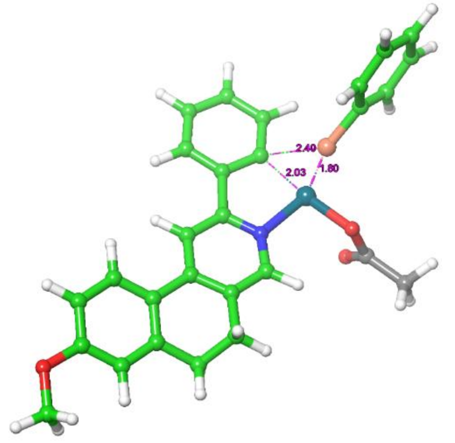

| | |
|---|---|
| Sum of electronic and thermal Free Energies= -8661.478025<br>Symbolic Z-marix:<br>Charge = 0 Multiplicity = 2<br>Pd 1.75124 -0.02475 0.<br>Se 1.14424 1.97425 -1.241<br>O 3.55024 0.08725 -0.718<br>C -0.00876 0.10425 0.997<br>C -1.02676 1.04525 0.886<br>C -0.09276 -0.95975 1.896<br>C -2.15076 0.91125 1.696<br>H -0.94676 1.87025 0.178<br>C -1.21976 -1.08575 2.703<br>C -2.24576 -0.14675 2.599<br>H -2.96076 1.63825 1.624<br>H -1.29976 -1.91075 3.411<br>H -3.12976 -0.24175 3.229<br>C 3.23124 -2.46575 1.009<br>C 1.13124 -1.95975 1.925<br>C 3.34524 -3.57275 1.845<br>H 4.02624 -2.21875 0.305<br>C 1.19524 -3.05175 2.782<br>C 2.31924 -3.87475 2.738<br>H 0.38124 -3.26075 3.476<br>C 4.59424 -4.41875 1.834<br>H 5.08524 -4.32375 0.865<br>H 5.26824 -4.07475 2.619 | Sum of electronic and thermal Free Energies= -8661.213201<br>Symbolic Z-matrix:<br>Charge = 0 Multiplicity = 2<br>Pd 1.75124 -0.02475 0.<br>Se 1.14424 1.97425 -1.241<br>O 3.55024 0.08725 -0.718<br>C -0.00876 0.10425 0.997<br>C -1.02676 1.04525 0.886<br>C -0.09276 -0.95975 1.896<br>C -2.15076 0.91125 1.696<br>H -0.94676 1.87025 0.178<br>C -1.21976 -1.08575 2.703<br>C -2.24576 -0.14675 2.599<br>H -2.96076 1.63825 1.624<br>H -1.29976 -1.91075 3.411<br>H -3.12976 -0.24175 3.229<br>C 3.23124 -2.46575 1.009<br>C 1.13124 -1.95975 1.925<br>C 3.34524 -3.57275 1.845<br>H 4.02624 -2.21875 0.305<br>C 1.19524 -3.05175 2.782<br>C 2.31924 -3.87475 2.738<br>H 0.38124 -3.26075 3.476<br>C 4.59424 -4.41875 1.834<br>H 5.08524 -4.32375 0.865 |

| | | | | | | | |
|---|---|---|---|---|---|---|---|
| C | 4.24124 | -5.88675 | 2.079 | H | 5.26824 | -4.07475 | 2.619 |
| H | 3.66624 | -6.26475 | 1.234 | C | 4.24124 | -5.88675 | 2.079 |
| H | 5.15824 | -6.46575 | 2.184 | H | 3.66624 | -6.26475 | 1.234 |
| C | 3.42124 | -6.02075 | 3.337 | H | 5.15824 | -6.46575 | 2.184 |
| C | 2.46624 | -5.05375 | 3.637 | C | 3.42124 | -6.02075 | 3.337 |
| C | 3.59624 | -7.12675 | 4.163 | C | 2.46624 | -5.05375 | 3.637 |
| C | 1.67024 | -5.20875 | 4.769 | C | 3.59624 | -7.12675 | 4.163 |
| C | 2.80824 | -7.27575 | 5.307 | C | 1.67024 | -5.20875 | 4.769 |
| H | 4.34824 | -7.87675 | 3.918 | C | 2.80824 | -7.27575 | 5.307 |
| C | 1.84624 | -6.31175 | 5.597 | H | 4.34824 | -7.87675 | 3.918 |
| H | 0.91024 | -4.46475 | 5.006 | C | 1.84624 | -6.31175 | 5.597 |
| H | 1.22224 | -6.42375 | 6.484 | H | 0.91024 | -4.46475 | 5.006 |
| N | 2.13924 | -1.71475 | 1.082 | H | 1.22224 | -6.42375 | 6.484 |
| C | 3.56024 | -0.47275 | -2.01 | N | 2.13924 | -1.71475 | 1.082 |
| O | 2.52724 | -1.02675 | -2.394 | C | 3.56024 | -0.47275 | -2.01 |
| C | 4.80224 | -0.42575 | -2.895 | O | 2.52724 | -1.02675 | -2.394 |
| C | 3.93424 | -9.35775 | 5.892 | C | 4.80224 | -0.42575 | -2.895 |
| H | 3.93024 | -10.14475 | 6.646 | C | 3.93424 | -9.35775 | 5.892 |
| H | 4.91324 | -8.87775 | 5.874 | H | 3.93024 | -10.14475 | 6.646 |
| H | 3.72524 | -9.79075 | 4.914 | H | 4.91324 | -8.87775 | 5.874 |
| O | 2.92424 | -8.37775 | 6.211 | H | 3.72524 | -9.79075 | 4.914 |
| H | 4.59024 | -0.91475 | -3.846 | O | 2.92424 | -8.37775 | 6.211 |
| H | 5.62324 | -0.94275 | -2.397 | H | 4.59024 | -0.91475 | -3.846 |
| H | 5.08324 | 0.61125 | -3.075 | H | 5.62324 | -0.94275 | -2.397 |
| C | 2.30424 | 3.40825 | -0.782 | H | 5.08324 | 0.61125 | -3.075 |
| C | 2.21124 | 4.61525 | -1.467 | C | 2.30424 | 3.40825 | -0.782 |
| C | 3.05724 | 5.66825 | -1.14 | C | 2.21124 | 4.61525 | -1.467 |

| | |
|---|---|
| C 3.99624 5.51625 -0.126<br>C 4.08924 4.31125 0.56<br>C 3.24424 3.25725 0.232<br>H 1.49424 4.73225 -2.241<br>H 2.98624 6.58825 -1.664<br>H 4.64224 6.32025 0.124<br>H 4.80724 4.19725 1.336<br>H 3.31524 2.33925 0.755 | C 3.05724 5.66825 -1.14<br>C 3.99624 5.51625 -0.126<br>C 4.08924 4.31125 0.56<br>C 3.24424 3.25725 0.232<br>H 1.49424 4.73225 -2.241<br>H 2.98624 6.58825 -1.664<br>H 4.64224 6.32025 0.124<br>H 4.80724 4.19725 1.336<br>H 3.31524 2.33925 0.755 |
| 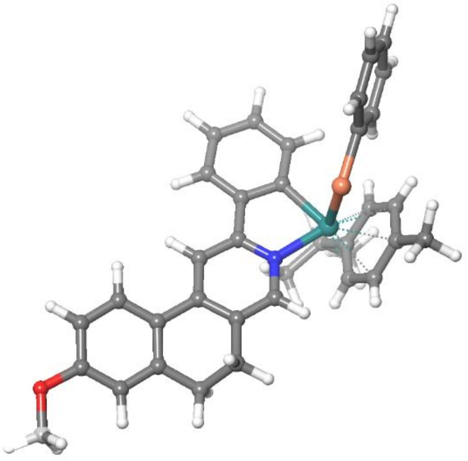<br>Sum of electronic and thermal Free Energies= -8327.027784<br>Symbolic Z-matrix:<br>Charge = 0 Multiplicity = 1<br>Ru 1.05817 1.13861 0.<br>C 0.18417 1.77461 1.786<br>C 0.98817 0.98061 -2.222<br>C 1.77017 -0.05539 -1.678<br>H 2.66117 -0.19339 -1.976 | 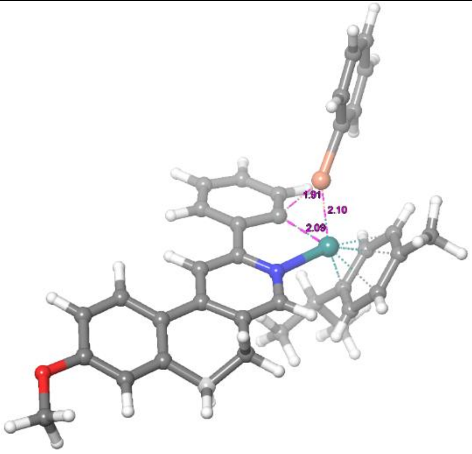<br>Sum of electronic and thermal Free Energies= -8326.831862<br>Symbolic Z-matrix:<br>Charge = 0 Multiplicity = 1<br>Ru 6.52847 -1.54703 0.<br>C 5.65447 -0.91103 1.786<br>C 6.45847 -1.70503 -2.222<br>C 7.24047 -2.74103 -1.678<br>H 8.13147 -2.87903 -1.976 |

| | | | | | | | |
|---|---|---|---|---|---|---|---|
| C | 1.20317 | -0.88939 | -0.674 | C | 6.67347 | -3.57503 | -0.674 |
| H | 1.71817 | -1.60739 | -0.324 | H | 7.18847 | -4.29303 | -0.324 |
| C | -0.08283 | -0.67939 | -0.192 | C | 5.38747 | -3.36503 | -0.192 |
| C | -0.85483 | 0.37561 | -0.804 | C | 4.61547 | -2.31003 | -0.804 |
| H | -1.74383 | 0.52761 | -0.508 | H | 3.72647 | -2.15803 | -0.508 |
| C | -0.33483 | 1.16861 | -1.804 | C | 5.13547 | -1.51703 | -1.804 |
| H | -0.87183 | 1.83961 | -2.209 | H | 4.59847 | -0.84603 | -2.209 |
| C | 1.54617 | 1.83261 | -3.309 | C | 7.01647 | -0.85303 | -3.309 |
| H | 1.46817 | 1.27761 | -4.255 | H | 6.93847 | -1.40803 | -4.255 |
| H | 0.97317 | 2.76761 | -3.396 | H | 6.44347 | 0.08197 | -3.396 |
| H | 2.61317 | 2.01761 | -3.121 | H | 8.08347 | -0.66803 | -3.121 |
| C | -0.75883 | -1.50139 | 0.883 | C | 4.71147 | -4.18703 | 0.883 |
| H | -1.42883 | -0.84939 | 1.463 | H | 4.04147 | -3.53503 | 1.463 |
| C | -1.59183 | -2.61039 | 0.233 | C | 3.87847 | -5.29603 | 0.233 |
| H | -2.09183 | -3.19539 | 1.016 | H | 3.37847 | -5.88103 | 1.016 |
| H | -2.34483 | -2.16439 | -0.431 | H | 3.12547 | -4.85003 | -0.431 |
| H | -0.92983 | -3.26539 | -0.35 | H | 4.54047 | -5.95103 | -0.35 |
| C | 0.27017 | -2.18139 | 1.818 | C | 5.74047 | -4.86703 | 1.818 |
| H | 0.89717 | -1.41539 | 2.294 | H | 6.36747 | -4.10103 | 2.294 |
| H | -0.26583 | -2.75039 | 2.593 | H | 5.20447 | -5.43603 | 2.593 |
| H | 0.90317 | -2.86939 | 1.239 | H | 6.37347 | -5.55503 | 1.239 |
| C | -1.11083 | 2.26461 | 1.946 | C | 4.27347 | -0.69403 | 2.088 |
| H | -1.75883 | 2.34761 | 1.118 | H | 3.54747 | -0.71403 | 1.309 |
| C | -1.56283 | 2.64761 | 3.206 | C | 3.88047 | -0.35903 | 3.381 |
| H | -2.55583 | 3.02761 | 3.326 | H | 2.85047 | -0.14603 | 3.586 |
| C | -0.72683 | 2.53761 | 4.313 | C | 4.81247 | -0.30303 | 4.414 |
| H | -1.08083 | 2.83361 | 5.281 | H | 4.49147 | -0.05303 | 5.409 |

| | | | | | | | |
|---|---|---|---|---|---|---|---|
| C | 0.56117 | 2.03361 | 4.161 | C | 6.14447 | -0.62703 | 4.17 |
| H | 1.19617 | 1.94061 | 5.007 | H | 6.82747 | -0.66103 | 4.982 |
| C | 1.00017 | 1.65361 | 2.894 | C | 6.55547 | -0.95703 | 2.88 |
| C | 2.30317 | 1.09861 | 2.613 | C | 7.82647 | -1.50703 | 2.594 |
| C | 3.25517 | 0.91161 | 3.611 | C | 8.80047 | -1.66803 | 3.577 |
| H | 3.04017 | 1.17361 | 4.62 | H | 8.61847 | -1.35303 | 4.576 |
| C | 4.48617 | 0.36961 | 3.27 | C | 10.00647 | -2.26203 | 3.237 |
| C | 4.72317 | 0.05861 | 1.937 | C | 10.19447 | -2.65303 | 1.918 |
| C | 3.72917 | 0.27461 | 0.986 | C | 9.17847 | -2.46003 | 0.986 |
| H | 3.92717 | 0.04361 | -0.031 | H | 9.34447 | -2.74903 | -0.022 |
| N | 2.53517 | 0.77461 | 1.337 | N | 8.00547 | -1.91103 | 1.337 |
| C | 6.08417 | -0.46639 | 1.533 | C | 11.52647 | -3.24103 | 1.507 |
| H | 6.04817 | -0.94739 | 0.542 | H | 11.44947 | -3.77103 | 0.545 |
| H | 6.77117 | 0.39261 | 1.487 | H | 12.23847 | -2.40703 | 1.4 |
| C | 6.62817 | -1.40539 | 2.613 | C | 12.06947 | -4.14003 | 2.623 |
| H | 7.64017 | -1.78039 | 2.374 | H | 13.06147 | -4.56103 | 2.378 |
| H | 5.93917 | -2.25939 | 2.675 | H | 11.35547 | -4.96603 | 2.747 |
| C | 6.61317 | -0.71739 | 3.955 | C | 12.11247 | -3.38303 | 3.926 |
| C | 5.55517 | 0.12761 | 4.281 | C | 11.09247 | -2.48503 | 4.232 |
| C | 7.63017 | -0.96739 | 4.871 | C | 13.14247 | -3.62203 | 4.83 |
| H | 8.41517 | -1.62139 | 4.592 | H | 13.89747 | -4.31703 | 4.568 |
| C | 7.59617 | -0.36839 | 6.133 | C | 13.16247 | -2.95703 | 6.059 |
| C | 6.53317 | 0.46961 | 6.457 | C | 12.13747 | -2.06503 | 6.362 |
| H | 6.49717 | 0.92661 | 7.417 | H | 12.14247 | -1.55703 | 7.298 |
| C | 5.51517 | 0.71661 | 5.541 | C | 11.10547 | -1.83003 | 5.46 |
| H | 4.71317 | 1.36061 | 5.811 | H | 10.33347 | -1.14503 | 5.714 |
| Se | 1.51017 | 3.43561 | -0.642 | Se | 5.73547 | 0.36097 | 0.359 |

| | | | | | | | |
|---|---|---|---|---|---|---|---|
| O | 8.59817 | -0.57239 | 7.133 | O | 14.18147 | -3.14503 | 7.045 |
| C | 9.68717 | -1.44839 | 6.772 | C | 15.22647 | -4.08103 | 6.707 |
| H | 10.38617 | -1.52739 | 7.617 | H | 15.94347 | -4.14203 | 7.538 |
| H | 10.21617 | -1.03939 | 5.899 | H | 15.74847 | -3.74103 | 5.8 |
| H | 9.29317 | -2.44639 | 6.53 | H | 14.78847 | -5.07503 | 6.53 |
| C | -0.15883 | 4.41761 | -0.752 | C | 5.00347 | 2.10197 | 0.703 |
| C | -1.46383 | 3.92161 | -0.77 | C | 3.62347 | 2.27597 | 0.662 |
| C | -2.55583 | 4.77861 | -0.862 | C | 3.06347 | 3.52397 | 0.909 |
| C | -2.36483 | 6.15161 | -0.94 | C | 3.88347 | 4.60597 | 1.2 |
| C | -1.07583 | 6.66061 | -0.925 | C | 5.26247 | 4.43697 | 1.245 |
| C | 0.01017 | 5.79861 | -0.833 | C | 5.82447 | 3.18897 | 0.998 |
| H | -1.64383 | 2.90261 | -0.713 | H | 2.99247 | 1.45197 | 0.439 |
| H | -3.54083 | 4.38161 | -0.873 | H | 2.00847 | 3.64897 | 0.874 |
| H | -3.19983 | 6.80761 | -1.011 | H | 3.45747 | 5.55897 | 1.388 |
| H | -0.91883 | 7.70961 | -0.985 | H | 5.88947 | 5.26397 | 1.466 |
| H | 0.99317 | 6.20261 | -0.824 | H | 6.87647 | 3.07497 | 1.033 |

**The limit of detection (LOD):**

The limit of detection (LOD) was determined by incrementally adding very low equivalents of picric acid (PA) to the sample. The LOD was calculated using the standard equation.

$$\text{LOD} = 3 \times \frac{\sigma}{S}$$

Where, σ represents the standard deviation of the analytical response and S denotes the slope of the calibration curve. The LOD value was subsequently obtained using this relationship.

Using the graph and equation LOD value is calculated and the LOD value is 0.29 µM.

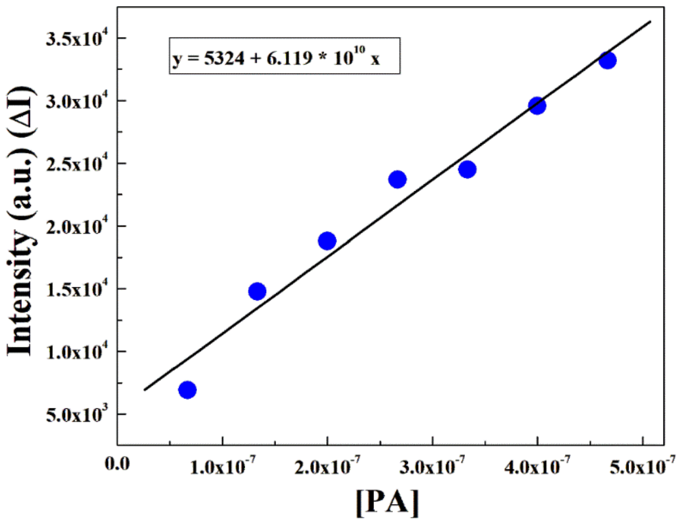


**Figure S1:** The limit of detection (LOD)

**Absorption spectra toward picric acid sensing:**

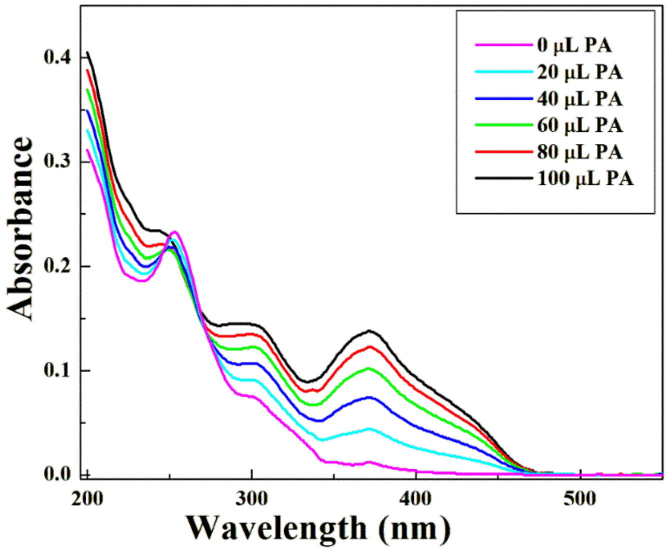


**Figure S2:** Absorption spectra toward picric acid sensing

The initial concentration of the sample was 8.33 µM. Upon incremental addition of picric acid, a new absorption band appeared at approximately 370 nm. The absorbance of this band (370nm) increased progressively with higher concentrations of picric acid. This emerging absorption feature can be attributed primarily to the formation of a picric acid–molecule complex.
